# Exploring the Microbial Reservoir of *Geodia cydonium* (Linnaeus, 1767): Insights into Site-Specific Diversity and Biotechnological Potential

**DOI:** 10.3390/md24010002

**Published:** 2025-12-19

**Authors:** Roberta Esposito, Roberta Trani, Marco Bertolino, Michele Sonnessa, Gaia Laurenzi, Valerio Zupo, Caterina Longo, Maria Costantini

**Affiliations:** 1 Department of Ecosustainable Marine Biotechnology, Stazione Zoologica Anton Dohrn, Villa Comunale, 80121 Naples, Italy; 2 Department of Bioscience, Biotechnologies and Environment, University of Bari Aldo Moro, 70121 Bari, Italy; roberta.trani@uniba.it (R.T.); caterina.longo@uniba.it (C.L.); 3 Department of Earth, Environmental and Life Sciences, University of Genoa, 16132 Genoa, Italy; 4 Bio-Fab Research Srl, 00161 Rome, Italy; 5 Independent Researcher, 00161 Rome, Italy; 6 Department of Ecosustainable Marine Biotechnology, Ischia Marine Centre, Stazione Zoologica Anton Dohrn, Via Francesco Buonocore 42, 80077 Ischia, Italy

**Keywords:** *Geodia cydonium*, metataxonomic, bacteria, marine biotechnology, sponges

## Abstract

Marine sponges are recognized as reservoirs of diverse microorganisms that produce bioactive natural compounds. In this study, we conducted a metataxonomic analysis of *Geodia cydonium* specimens collected from four sites in Italy: Secca delle Fumose (Gulf of Naples, Tyrrhenian Sea), Mar Piccolo of Taranto and an Integrated Multi-Trophic Aquaculture (IMTA) system in Mar Grande of Taranto (both located in the Ionian Sea), and Polignano a Mare (Adriatic Sea). Our results revealed a highly diverse microbial community within the sponges, encompassing 24 bacterial phyla. Among these, Chloroflexota was the most abundant phylum, accounting for an average of 30.2% of the total community across all samples. In addition, the majority of the microbiota was composed of Actinomycetota, Proteobacteria, Acidobacteriota, Poribacteriota, Gemmatimonadota, and Dadabacteria. The sponge sample from Polignano a Mare exhibited the richest and most diverse bacterial community. This observation was supported by phylogenetic analysis, which identified seven bacterial genera, *Albidovulum*, *Filomicrobium*, *Microtrix*, *Gaiellales*, *D90* (Gammaproteobacteria class), and *Blastopirellula*, exclusive to this site. Several of these taxa are known for their potential biotechnological applications, underlining the significance of site-specific microbial diversity in *G. cydonium*.

## 1. Introduction

Sponges are fundamental components of benthic communities in both freshwater and marine environments, where they play a pivotal role in nutrient cycling and water filtration. Their exceptional filter-feeding capacity enables them to process large volumes of water and efficiently retain suspended organic particles [1]. The complex aquiferous canal system, lined with specialized choanocyte cells, captures a wide range of particulate organic matter (POM), including bacteria [2,3], viruses [4,5], and other microplanktonic particles [6,7]. In addition to POM, many sponge species are also capable of incorporating dissolved organic carbon (DOC) from seawater, thereby utilizing both particulate and dissolved fractions of organic matter as nutritional resources. This dual capacity for POM and DOC uptake allows sponges to play a unique ecological role in linking the dissolved and particulate organic pools within marine food webs. Furthermore, due to their ability to accumulate and retain substances from the surrounding water, sponges can bioaccumulate various environmental pollutants such as surfactants [8], heavy metals [9,10,11,12,13], and polychlorinated biphenyls [14]. This capacity highlights their potential as bioindicators of environmental quality and as active agents in pollutant removal. The combined filtration of POM and DOC, along with the bioaccumulation of contaminants, underscores the ecological importance of sponges as mediators of biogeochemical fluxes and as regulators of water quality and productivity in benthic ecosystems. As regards inorganic nutrients, most sponge species incorporate inorganic silicon in the form of dissolved silicic acid to produce biogenic silica, the main component of their siliceous skeletons [15,16]. In addition, some sponges can assimilate dissolved inorganic carbon (C), nitrogen (N), and phosphorus (P); however, unlike silicon uptake, these processes are largely mediated by their symbiotic microbial communities. Such microbial consortia include photoautotrophs (e.g., cyanobacteria) and methanotrophic and sulfur-oxidizing bacteria, as well as nitrifying and other chemoautotrophic microbes which fix and transfer inorganic nutrients to the sponge host [17,18].

Sponges host diverse microbial communities, including bacteria, viruses, and archaea, which are able to produce secondary metabolites contributing to their defense against pathogens and predators [19]. Beyond their ecological importance, these symbiotic microorganisms have attracted significant attention due to their production of secondary metabolites with promising biotechnological applications [20]. Between 1985 and 2008 alone, over 3500 novel compounds were isolated from sponges, positioning them as one of the richest sources of structurally unique and biologically active natural products distinct from terrestrial organisms [21,22]. Such findings support the potential of marine sponges as reservoirs for novel therapeutic agents derived from their associated microorganisms [23].

The Mediterranean Sea, characterized by limited hydrological exchange and a prolonged water residence time, is warming at two to three times the global ocean rate, making it a hotspot for climate change impacts [24,25,26,27]. As a biodiversity hotspot, the Mediterranean hosts a significant proportion of European marine endemic species [28], placing it at significant risk of biodiversity loss with serious implications for ecosystem services such as water quality, fisheries, tourism, and coastal protection [29]. Environmental stressors including temperature fluctuations, ecological pressures, and other biotic and abiotic factors can alter sponge physiology and microbial community structure, sometimes leading to pathogen proliferation, impaired defense mechanisms, and also, sponge mortality [30].

To support sustainable sponge biomass production for the species interesting from the biotechnological point of view, improvements in aquaculture techniques are essential. Integrated Multi-Trophic Aquaculture (IMTA) offers an innovative approach by co-cultivating extractive species (e.g., algae and marine invertebrates) alongside fed species (e.g., fish), thereby mitigating environmental impacts such as nutrient loading and sedimentation typically associated with traditional aquaculture [31]. This method is also considered a promising strategy to reduce overexploitation of natural sponge populations [32].

In this study, we aimed to investigate the bacterial communities associated with *Geodia cydonium* (Linnaeus, 1767) specimens collected from different locations, in order to evaluate their potential for biotechnological exploitation or other possible applications. In particular, we present the first comprehensive metataxonomic analysis of bacterial communities associated with *G. cydonium* collected from four sites along southern Italian coasts: Secca delle Fumose (Gulf of Naples, Tyrrhenian Sea), Mar Piccolo of Taranto and an Integrated Multi-Trophic Aquaculture (IMTA) system in Mar Grande of Taranto (both located in the Ionian Sea), and Polignano a Mare (Adriatic Sea). Phylogenetic identification of over 1000 bacterial isolates was performed to investigate potential host- and location-specific microbial patterns. Additionally, Amplicon Sequence Variant (ASV) analysis was employed to assess the diversity and composition of sponge-associated microbiomes across different environments. Due to ethical, logistical, and conservation considerations, only one *G. cydonium* individual per site was collected. The species is slow-growing and forms part of long-term monitoring activities in the Mar Piccolo and IMTA areas, where collection is restricted to avoid disturbance. For this reason, the present study is framed as an exploratory, qualitative survey of the sponge-associated microbiota. The absence of biological replicates prevents statistical inference on population-level differences, and all inter-site comparisons should therefore be interpreted as descriptive trends requiring confirmation through future replicated studies.

*G. cydonium* is a long-lived, habitat-forming sponge that provides shelter and substrate for numerous benthic organisms. It exhibits an Atlantic–Mediterranean distribution, occurring on artificial and natural hard substrates such as rocky outcrops, coralligenous reefs, marine caves, and soft sediments. The species demonstrates remarkable ecological tolerance, thriving in semi-enclosed basins, areas with high sedimentation, nutrient enrichment, and low oxygen levels [33]. This resilience is exemplified by populations in Mar Piccolo of Taranto, Italy, which have persisted and flourished for over 45 years in a highly degraded environment, representing one of the richest and most stable *G. cydonium* populations in the Mediterranean Sea [33].

The cultivation of *G. cydonium* remains challenging due to its slow growth and complex symbiotic relationships [34]. Current research is evaluating its mariculture potential within Integrated Multi-Trophic Aquaculture (IMTA) systems in Mar Grande of Taranto near floating fish cages [35] and its bioremediation potential [36]. *G. cydonium* has been reported to produce bioactive compounds with anti-inflammatory properties [37]. Although several studies have investigated the microbial communities associated with Mediterranean sponges, research on *G. cydonium* has thus far been limited to single-site analyses, providing an incomplete picture of its microbiome structure and variability. This study offers the first multi-site, cross-basin metataxonomic characterization of *G. cydonium* collected from four ecologically distinct regions spanning the Tyrrhenian, Ionian, and Adriatic Seas. Importantly, it also provides the first characterization of the microbiota of *G. cydonium* cultivated in an IMTA system, allowing us to assess how mariculture conditions influence its symbiotic assemblages. Our analysis reveals previously unreported bacterial phyla and site-exclusive genera, including several taxa with known ecological relevance or biotechnological potential. By integrating ASV-based community profiling with extensive culturing and phylogenetic identification of over 1000 isolates, this study advances our understanding of species-specific versus environment-driven microbial patterns in *G. cydonium* and highlights its value as a reservoir of novel microbial diversity.

## 2. Results and Discussion

### 2.1. Taxonomic Identification of G. cydonium Associated Bacterial Community

Some studies have reported the high bioactivity and biodiversity of bacteria associated with marine sponges collected from the Mediterranean Sea, belonging to the class Demospongiae [38,39,40]. Several species within this class are categorized as high-microbial-abundance (HMA) organisms, corresponding to marine sponges characterized by a high density of microbial symbionts, typically on the order of 10^8^–10^10^ microbial cells per gram of sponge tissue, which can constitute up to 40% of the sponge’s total biomass [41,42,43]. This was the case for the sponges in [38], for example, which revealed a highly diverse bacterial community associated with the Mediterranean sponge *Erylus discophorus* and *G. cydonium* belonging to the order Tetractinellida [38,42,44].

In this exploratory study, we aimed to characterize the microbiota diversity of *G. cydonium*, collecting one specimen per site across four southern Italian coastal locations. We recognize that this design limits statistical inference and comparison among the sites; therefore, conclusions regarding site-specific differences are presented as preliminary observations. In addition, it should be noted that seawater and sediment controls were not included in this study. Therefore, the patterns observed here are preliminary and exploratory, and the microbial taxa identified cannot be conclusively attributed to stable host-specific associations.

A total of 24 bacterial phyla were identified across all samples. The highest phylum-level diversity was observed in the Mar Piccolo (MP) specimen, where 20 phyla were detected, followed by NAP with 18, IMTA with 17, and POL with 16 phyla. Despite this variability, the bacterial community was consistently dominated by Chloroflexota, Actinomycetota, Proteobacteria, and Acidobacteriota, although their relative abundances differed among sites. For instance, Proteobacteria were particularly abundant in the NAP specimen, whereas Chloroflexota and Actinomycetota predominated in the IMTA and POL samples. Less abundant phyla, such as Poribacteriota, Nitrospirota, Planctomycetota, and Myxococcota, were detected in all samples at lower relative frequencies. While Proteobacteria were particularly abundant in NAP, Chloroflexota and Actinomycetota predominated in IMTA and POL samples; these observations should be interpreted cautiously due to single-specimen sampling.

**Figure 1:**
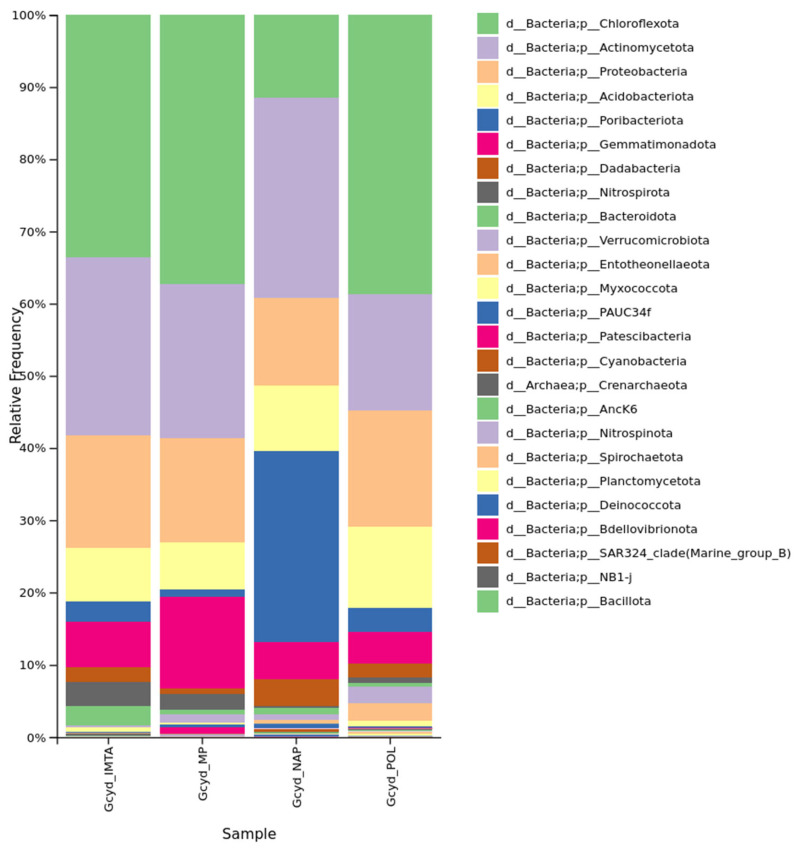
Taxonomy bar plot showing the relative abundance of the most prevalent bacterial taxa at the phylum level in the four samples of the sponge *G. cydonium* collected in the IMTA system, Mar Piccolo (MP), Secca delle Fumose (NAP), and Polignano a Mare (POL).

On the whole, the phylum Chloroflexota was the most common, making up an average of 30.2% of the microbial community in the *G. cydonium* samples collected from the four sampling sites. It was followed by Actinomycetota (22.4%), Proteobacteria (14.6%), Acidobacteriota (8.6%), Poribacteriota (8.3%), Gemmatimonadota (7.1%), and Dadabacteria (2.1%). The remaining phyla were less represented, each accounting for less than 2% of the total bacterial community, as shown in Figure 1. Among the 24 bacterial phyla identified, the 7 dominant ones (mentioned above) accounted for 93.3% of the total microbial community, while the remaining phyla collectively represented only 6.7% of the microbiota. These results suggest the presence of a preliminary core microbiota within *G. cydonium*, consistent across the four sampled sites. However, without replication, the extent of host specificity and environmental influence remains uncertain.

These seven phyla represented the core microbial community consistently associated with *G. cydonium* across all of the collection sites. In fact, they were present in all of the samples even if with a different relative frequency, which was very similar for the three samples collected in Puglia in comparison with the sample from the Gulf of Naples. Their relative abundances were remarkably similar among the three Apulian samples (Mar Piccolo, IMTA, and Polignano a Mare), despite the contrasting environmental settings, from the oligo-eutrophic, semi-enclosed basin of Mar Piccolo [45], to the IMTA site influenced by aquaculture-derived organic inputs [46], and the oligotrophic, well-oxygenated waters of the Adriatic karstic coast at Polignano a Mare [47]. This pattern was consistent with the presence of species-specific symbionts, tightly integrated with the sponge hosts, as widely documented in previous studies, although replication is necessary to confirm this. For instance, some authors showed that the Antarctic sponge *Mycale* (*Oxymycale*) *acerata* Kirkpatrick, 1907 had a very constant core microbiota across six sampling locations that were geographically separated [48]. The existence of preserved and host-specific microbial assemblages in sponges is further supported [49], showing that the prokaryotic communities linked to the sponge *Monanchora arbuscula* and *Xestospongia muta* remained constant even if collected in different environments. Although Chloroflexota represented the most abundant phylum within the microbiota of *G. cydonium* in the present study, it was not detected in other congeneric sponge species, such as *Geodia barretti* Bowerbank, 1858, collected in Norway [50]. This observation indicates the potential for species-specific or environmentally driven differences in microbial community composition within the *Geodia* genus. The ASV (Amplicon Sequence Variant) analysis revealed that all of the taxa were identified with an average confidence level around 94% (min 75%, max 100%). The full taxonomic breakdown of the microbial communities associated with sponge samples is provided in Tables S1–S4. Among the samples collected, the following was found:

i. The largest number of features (300) in *G. cydonium* collected in Polignano a Mare, especially Anaerolineae (5%), Dehalococcoidia (3%), Acidomicrobiia (3%), Gammaproteobacteria (3%), and Acidobacteriota (1%).
ii. 232 ASVs in *G. cydonium* sampled in Secca delle Fumose (Naples) with a greater abundance of Acidomicrobiia (6%), Poribacteriota (6%), Gammaproteobacteria (2%), Dehalococcoidia (1%), and Thermoanaerobaculia (1%).
iii. 148 ASVs in the sample of *G. cydonium* collected in the IMTA system, where Acidomicrobiia (7%), Anaerolineae (7%), Gammaprotebacteria (3%), Dehalococcoidia (2%), Alphaproteobacteria (2%), Acidobacteriota (2%), and BD2-11_terrestrial_group (Gemmatimonadota phylum) were highly represented.
iv. The sample of *G. cydonium* with the least number of features was sampled in Mar Piccolo and it revealed seven bacterial groups (Anaerolineae (7%), Acidomicrobiia (6%), BD2_11_terrestrial group (3%), Dehalococcoidia, Gammaproteobacteria, Alphaproteobacteria, and Acidobacteriota (2%, each)) (Figures S1 and S2).

Our findings showed differences between the sample of *G. cydonium* collected in Secca delle Fumose (Parco Sommerso di Baia, Naples) analyzed in our previous work [38] and the sample of *G. cydonium* collected in the same area and analyzed in this study, concerning the presence or absence of phyla and their abundances. In particular, the phyla Taumarchaeota and Acidobacteriota identified in the microbiota of *G. cydonium* [38] were not detected in the sample of *G. cydonium* under analysis in this study. On the contrary, eleven phyla (Crenarchaeota, AncK6, Spirochaetota, Deinococcota, Entotheonellaeota, Bdellovibrionota, Myxococcota, SAR324, NBI-J, Patescibacteria, and Bacillota) present in the sample of *G. cydonium* were not previously detected [38]. The main two differences concerned the genus Gemmatimonadates (part of the phyla Gemmatimonadota), which in the sample of *G. cydonium* analyzed in this study seemed to be more abundant respect to the previous analysis [38]. In contrast, the phylum Nitrospirota appeared to be less abundant in the sample explored in this study than that considered in our previous work [38]. These differences are likely attributable to methodological variations, including library preparation and bioinformatic pipelines.

### 2.2. Relationship Between Abundance and Distribution of the Bacterial Communities Among Sites

**Figure 2:**
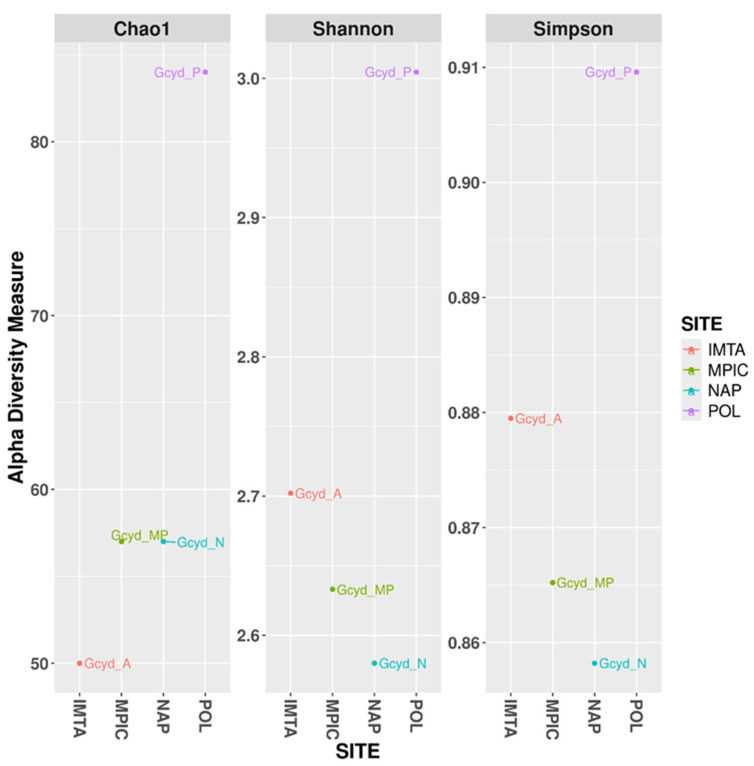
Alpha diversity plots illustrating differences among four samples of *Geodia cydonium* collected in the IMTA system (IMTA), Mar Piccolo (MPIC), Secca delle Fumose (NAP), and Polignano a Mare (POL).

Alpha diversity was investigated in order to assess the microbial community structure of the different *G. cydonium* samples, employing three complementary indices (Chao1, Shannon, and Simpson), which together provide insights into species richness, diversity, and evenness, as well as allowing the evaluation of sequencing depth adequacy. As reported in Figure 2, the Chao1 index for the specimen collected within the IMTA system was consistently lower than that observed in the other samples under analysis, suggesting a reduced richness of taxa.

**Figure 3:**
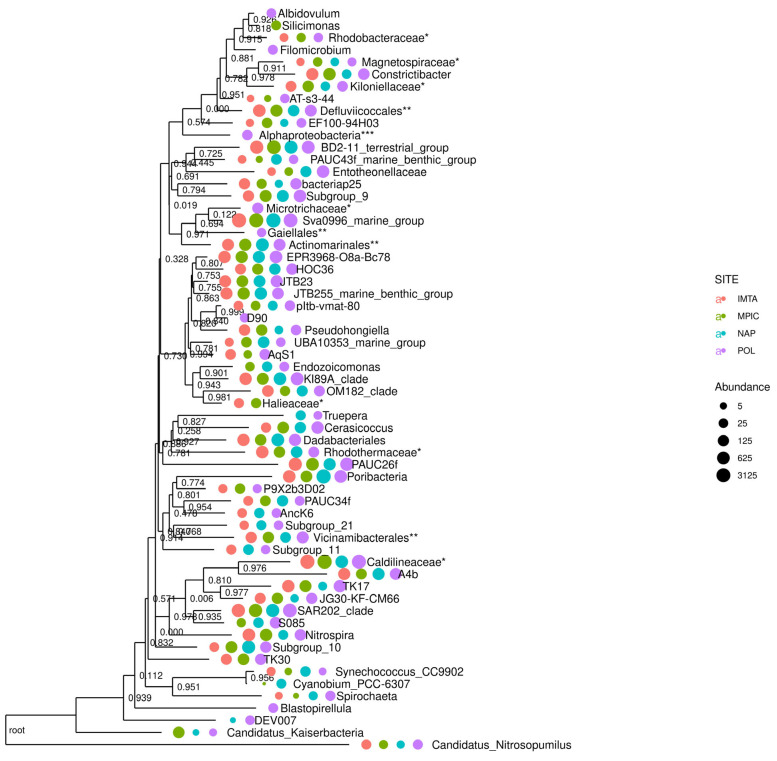
Phylogenetic tree of genera reporting the distance and their abundance in the sample of sponges collected in the IMTA system (IMTA), Mar Piccolo (MPIC), Secca delle Fumose (NAP), and Polignano a Mare (POL). Each asterisk refers to a taxonomic level prior to that of the genus. Abundance is measured as the number of ASVs.

**Figure 4:**
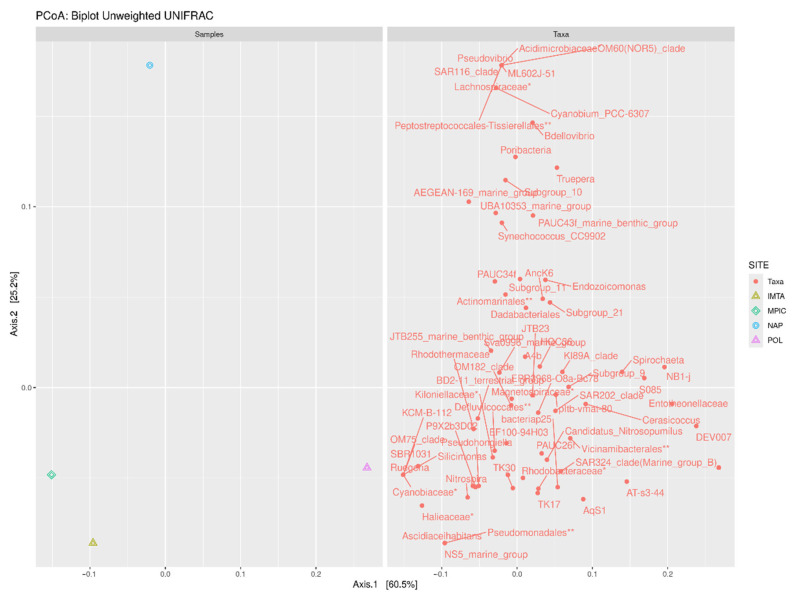
PCoA, combined plot with both taxa and samples by the phylogenetic distance metric Unweighted UniFrac using R v4.5.1 (accessed on 13 June 2025). NAP sample is at a greater distance than the other samples. Each asterisk refers to a taxonomic level prior to that of the genus.

Among the *G. cydonium* samples from the IMTA system, Mar Piccolo, and Secca delle Fumose, the Polignano a Mare specimen displayed a notably higher Chao1 value, reflecting greater community richness. These results are further supported by the fact that both the Shannon and Simpson diversity indices for the Polignano a Mare sample were higher than those of the other sites. This indicates not only a greater number of taxa, but also a more even distribution of species abundances within the community. Taken together, the findings from all three indices suggest that, among all the locations analyzed, the microbial assemblage associated with *G. cydonium* from Polignano a Mare exhibited the highest level of diversity. Given the single-sample-per-site design, these patterns are reported as exploratory observations rather than definitive site-specific conclusions. Furthermore, without environmental controls, it is not possible to separate genuine host-specific taxa from transient or waterborne bacteria. Therefore, while the data suggest potential site-associated differences, they cannot be considered conclusive evidence of host specificity. This trend is corroborated by the phylogenetic tree, which illustrates the relative abundances of the most prevalent genera across the four analyzed *G. cydonium* samples (Figure 3 and Figure 4; see also Figure S3).

In particular, seven genera and one class of bacteria were detected only in the sample of *G. cydonium* collected in Polignano a Mare—*Albidovulum*, *Filomicrobium*, *Microtrix*, *Gaiellales*, *D90*, *Blastopirellula,* and *Alphaproteobacteria*—with high biotechnological interest. These occurrences highlighted potential site- or habitat-specific taxa; however, additional specimens are required to confirm whether these differences reflect true environmental effects or individual variation, as well as studies including seawater and sediment controls to confirm host specificity. The genus *Albidovulum* was isolated from the marine hot spring at Ferraria on the island of Sao Miguel, the Azores [51]. Then, thermophilic bacteria belonging to this genus were isolated from various environments such as hot springs and hypersaline environments, but also in samples of sediments and sponges as in the case of *Xestospongia muta* and *Agelas sventres* Lehnert & van Soest, 1996 [52,53,54]. Moreover, the bacterial strain *Albidovulum* sp. SLM16 isolated from a mixed seawater–sand–sediment sample collected from a coastal fumarole located in Whalers Bay (Antarctica) showed amine-transaminase activity, which can be important for biotechnological applications to develop drugs, insecticides, and functional polymers [55]. Planctomycetes, including the genus *Blastopirellula*, traditionally recognized as environmental bacteria were detected in association with sediments of marine, sponges, corals, prawns [56,57,58], ascidians [59], desert soils [60,61], hypersaline environments [62,63], hydrocarbon-polluted environments [64], and in the rhizosphere of several plants [65,66,67]. These bacteria have recently been associated with human pathology as opportunistic pathogens, arousing great interest among clinical microbiologists [68]. However, their ability to adapt to very different environments also makes them versatile and suitable for the production of bioactive secondary metabolites such as terpenoids [69] and other compounds such as 3,5-dibromo-p-anisic acid and stieleriacines which have anti-cancerous properties [70,71,72]. Moreover, the genera *Synechococcus*, *Rhodopirellula*, *Blastopirellula*, and *Rubripirellula* associated with the marine sponges *Haliclona* sp. CH01, *Cladocroce* sp. CH02, *Sigmaxinella* sp. QZ01, and *Lissodendoryx* sp. MX01 can have an important ecological role sequestering phosphorus in the form of polyphosphate and helping sponges to thrive in environments with extremely low nutrients [73,74].

The strain *Filomicrobium fusiforme* gen. nov., sp. nov. belonging to *Filomicrobium* genus, was isolated for the first time from brackish water of the Baltic Sea [75]. Then, another two strains, *Filomicrobium insigne* sp. nov. and *Filomicrobium marinum,* were isolated from other sources: an oil-polluted saline soil and a marine water enrichment [76,77]. From a biotechnological perspective, this genus represents a promising candidate for the biodegradation of methane sulfonate, utilizing it as a source of both carbon and sulfur [76]. Furthermore, its potential for plastic degradation is supported by its detection on Mater-Bi following 94 days of incubation in a minimal medium, where the bioplastic served as the sole source of carbon and energy [78,79].

Alphaproteobacteria are a very abundant class of bacteria in marine sponges. In particular, several species belonging to this class isolated from various sponges, *Halichondria* (*Halichondria*) *panicea* (Pallas, 1766), *Sarcotragus fasciculatus* (Pallas, 1766), *Axinella polypoides* Schmidt, 1862, *Acanthella* sp. [80], *Suberea mollis* (Row, 1911) [81], and *Dysidea avara* (Schmidt, 1862), showed antimicrobial and anti-vascular activities [82]. In addition, the authors of [83] recently demonstrated that these bacteria are able to transform bioavailable nitrogen to gaseous nitrogen into marine oxygen-deficient zones. In contrast to the bacterial genera described above, little is known about the *Gaiellales* genus and *Microtrichaceae* family and no data are reported for the genus of marine bacteria D90. In particular, the species *Gaiella occulta* Albuquerque, França, Rainey, Schumann, Nobre, da Costa, 2011, is the only one to be part of the genus *Gaiellales* as proposed in Ref. [84]. These bacteria were found in different environments, including weathered serpentine rock [85], mangrove wetlands [86], saline–alkaline soil [87], marine ascidians [88], and wastewater treatment plants [89].

The *Microtrichaceae* family was found in invertebrates such as corals [90], sponges [91,92], and also in insects and mammals [93]. However, it was not clear the role of these bacteria within invertebrates.

*Silicimonas algicola* gen. nov. sp. nov., isolated from *Thalassiosira delicatula* Ostenfeld, 1908, was proposed by some authors [94,95]. This genus of bacteria has also been found associated with corals. In fact, certain bacterial genera such *as Ruegeria*, *Methyloceanibacter*, *Filomicrobium*, *Halioglobus*, *Rubripirellula*, *Rhodopirellula*, *Silicimonas*, *Blastopirellula*, Sva0996 marine group, *Woeseia*, and unclassified_c_*Gammaproteobacteria* are more abundant in bleached corals than in unbleached ones [96].

Although our observations suggest functional potential, further work with replicated specimens and controlled experiments is needed to validate these preliminary findings. In fact, a major limitation of this study is the use of a single specimen per site. While this approach is common in initial microbiome surveys of ecologically sensitive and slow-growing HMA sponges, it restricts our capacity to disentangle individual variability from true environmental or biogeographical patterns. Therefore, all observed differences in richness, taxonomic composition, and the presence of rare taxa among sites must be considered preliminary. Future studies integrating multiple biological replicates per location, temporal sampling, and functional metagenomics will be essential to validate and extend the patterns identified here. Previous work has also characterized the microbiomes of Mediterranean sponges using only a few individuals per site, laying the groundwork for more in-depth, replicated sampling [38]. Using metatranscriptomics in *G. barretti,* microbial functionality was revealed in a high-microbial-abundance sponge, illustrating that even single-individual studies can yield valuable functional and ecological insight [40]. It should be noted that the sampled *G. cydonium* specimens were collected at different depths, ranging from 2.5 to 3 m at the Apulian sites and the IMTA system to 20 m at Secca delle Fumose (Naples). Depth-related environmental factors, including light availability, temperature, dissolved oxygen, and nutrient concentrations, may influence microbial community composition. Therefore, some of the observed differences in microbial assemblages could be partially attributable to depth effects rather than geographic or host-specific factors (Figure 4).

To further clarify this point, we note that the Apulian specimens (Mar Piccolo, IMTA, and Polignano a Mare) were collected at shallow depths of 2.5–3 m, whereas the Secca delle Fumose (Naples) specimen was collected at 20 m. These depth differences correspond to fundamentally distinct environmental regimes. Shallow-water habitats typically experience higher and more variable light levels, stronger temperature fluctuations, greater hydrodynamic disturbance, and stronger terrestrial or coastal inputs. In contrast, the 20 m site at Secca delle Fumose is characterized by reduced irradiance, more stable thermal conditions, and locally modified geochemistry due to hydrothermal emissions. These physicochemical contrasts may influence both free-living microbial assemblages and the sponge holobiont, potentially contributing to the distinct microbial profile observed in the Naples specimen. Because only a single individual was sampled per site, we cannot separate depth-driven variation from individual-level variability, and we highlight that future replicated sampling across depth gradients, together with seawater and sediment controls, will be essential to disentangle depth, habitat, and host-specific effects on *G. cydonium* microbiomes.

Despite some limitations, our results provide several advancements beyond previous studies on *G. cydonium* and other *Geodia* spp. First, this research represents the first comprehensive metataxonomic comparison of *G. cydonium* from multiple Mediterranean sites, revealing a consistent core microbiota across habitats but also strong site-specific patterns in microbial diversity. Second, it delivers the first description of the microbiota of *G. cydonium* grown in an IMTA system, in which we observed markedly reduced microbial richness, offering new insights relevant for sponge mariculture and bioremediation. Third, we document eleven bacterial phyla not previously reported for *G. cydonium* as well as several genera found exclusively in the Polignano a Mare specimen, many of which possess recognized ecological roles or potential biotechnological applications. These novel findings expand the known microbial repertoire associated with *G. cydonium*, underscore the influence of habitat on its symbiotic community, and open new perspectives for the discovery of functionally important or bioactive microbial taxa.

## 3. Materials and Methods

### 3.1. Studied Species and Collection

The demosponge *Geodia cydonium* (order Tetractinellida, family Geodiidae) commonly exhibits a globular or cushion-shaped morphology, although its form is highly variable, ranging from irregularly massive, flattened, columnar, or encrusting shapes to more insinuating forms. Typical specimens measure 10–20 cm in diameter, although exceptionally large individuals have been recorded, including one from the Northern Adriatic Sea reaching 180 cm in diameter [97]. The surface is whitish-grey or yellowish, tough but brittle, contrasting with a cream-to-orange interior. Oscules and ostia are grouped in cribral areas, with oscules measuring 1.5–2 mm in diameter. The skeleton is composed of siliceous spicules arranged into a distinct ectosome and choanosome. The ectosome forms a 2 mm thick cortex composed of microscleres, with protruding macroscleres contributing to a hispid surface. Within the choanosome, oxeas and triaenes are radially arranged and interspersed with oxyasters. Megascleres include large fusiform oxeas (occasionally modified into styles or strongyles), orthotriaenes, protriaenes, anatriaenes, and mesotriaenes, spanning a broad size range. Microscleres consist of sterrasters, spherasters, oxyasters, and chiasters, enhancing structural complexity.

**Figure 5:**
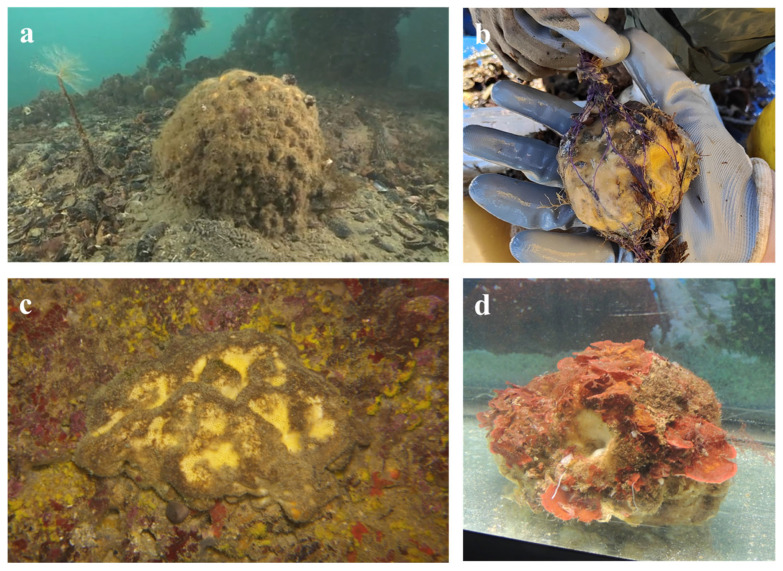
*Geodia cydonium* specimens collected at different sites: (**a**) Mar Piccolo of Taranto; (**b**) IMTA plant in Mar Grande of Taranto; (**c**) Polignano a Mare Colombi semi-submerged marine cave; (**d**) Secca delle Fumose (Gulf of Naples).

Samples of *G. cydonium* were collected from four sites in southern Italy: three along the Apulian coast and one along the Campanian coast (Figure 5).

**Figure 6:**
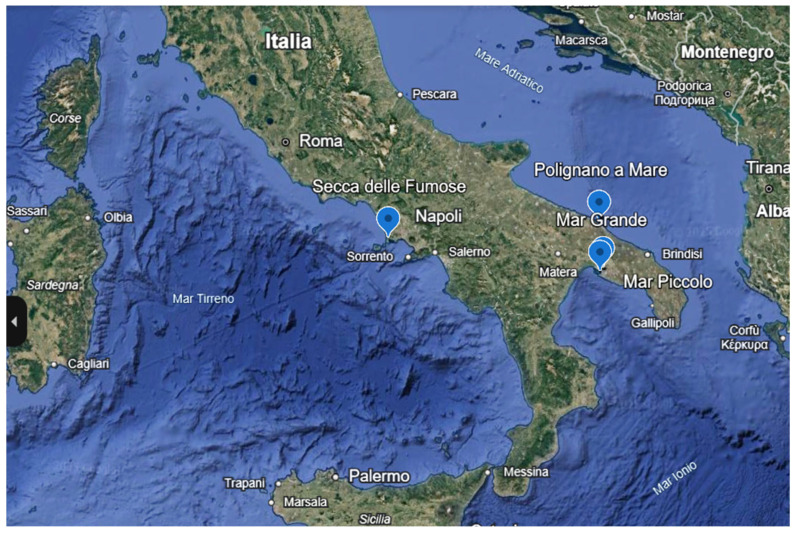
A map of the four sampling areas, according the coordinates reported in Table 1, constructed by Google Earth (https://www.google.it/intl/it/earth/index.html (accessed on 14 Novembre 2025)).

The Apulian samples were obtained from the Taranto Seas (Mar Piccolo and Mar Grande, Northern Ionian Sea) and from Polignano a Mare (Bari, Southern Adriatic Sea). The Campanian sample was collected from Secca delle Fumose, located within the Parco Sommerso di Baia (Naples, Southern Tyrrhenian Sea) (Table 1 and Figure 6).

*G. cydonium* specimens from the Taranto Seas (northern Ionian Sea) were collected from Mar Grande and Mar Piccolo. Mar Grande is an open, relatively deep bay that serves as the primary exchange zone with the Ionian Sea. It hosts significant maritime activities, including a commercial port, a naval base, and extensive mariculture facilities. Specimens of *G. cydonium* for this study were cultivated within the REMEDIA Life Integrated Multi-Trophic Aquaculture (IMTA) system at the “Maricoltura Mar Grande” facility. This system, located on the southern side of Mar Grande, consists of floating fish cages (7–12 m depth) used for farming European seabass (Dicentrarchus labrax) and gilthead sea bream (Sparus aurata). Sponge culture followed a low-impact methodology [32,34,35].

Mar Piccolo is a semi-enclosed, shallow coastal lagoon with a total surface area of 20.72 km^2^. It is divided by a promontory into two smaller inlets (First and Second Inlet), with maximum depths of 15 m and 9 m, respectively. This system is characterized by limited hydrodynamics, low seawater turnover, and significant freshwater input from both small streams and submarine springs (citri). These factors contribute to long water residence times, wide seasonal fluctuations in dissolved oxygen, and occasional hypoxic conditions, particularly in the deeper layers of the Second Inlet. Mar Piccolo acts as a sink for anthropogenic contaminants from industrial and urban sources. Despite this environmental degradation, it sustains unexpectedly rich and resilient benthic communities, most notably a stable and abundant population of the demosponge *G. cydonium* [33,98].

Samples from the Polignano a Mare site were collected in the Colombi Cave, situated on the Adriatic margin of the Apulian carbonate platform, which forms part of the exposed Apulian foreland. This coastal area is of particular geomorphological interest due to its karst features, including numerous coastal cavities and marine caves [99,100]. Among these, the Colombi Cave, located within the “Costa Ripagnola” Regional Natural Park, is a natural, semi-submerged marine cavity with a maximum depth of 7 m [101].

The Secca delle Fumose site is located within the Parco Sommerso di Baia (Southern Tyrrhenian Sea, Gulf of Naples) at a depth of approximately 20 m. The area lies within the volcanic complex of the Campi Flegrei caldera and is characterized by submarine hydrothermal activity, with fumaroles releasing CO_2_-rich gas and hot fluids that alter the local geochemistry and substrate composition. The seabed consists of archaeological structures (Roman pilae and masonry blocks) interspersed with coarse detrital sediments, providing abundant hard surfaces for epibenthic colonization. The site hosts diverse benthic assemblages, with macroalgae dominating the illuminated portions and filter-feeding invertebrates (sponges, bryozoans, polychaetes, ascidians, and cnidarians) prevailing in shaded areas. Despite the challenging environmental conditions associated with hydrothermal emissions, the community remains rich and structured, supporting species such as *G. cydonium* and other demosponges typical of coralligenous-like habitats [102].

Sponge specimens were collected by scuba divers and transported to the laboratory in dark, insulated containers under refrigerated conditions. Collected samples were immediately washed at least three times with filter-sterilized natural seawater. A fragment of each specimen was placed into individual sterile tubes and kept in RNAlater^©^ at −20 °C used for molecular analysis.

Due to ethical, logistical, and conservation considerations, only one *G. cydonium* individual per site was collected. The species is slow-growing and forms part of long-term monitoring activities in the Mar Piccolo and IMTA areas, where collection is restricted to avoid disturbance.

### 3.2. Metataxonomic DNA Extraction and Illumina MiSeq Sequencing

About 250 mg of tissue was weighted and used for DNA extraction by using the DNeasy^®^ PowerSoil^®^ Pro Kit (QIAGEN, Hilden, Germany), according to the manufacturer’s instructions [38]. The V3–V4 variable region (~460 bp) of 16S rRNA was amplified following the MiSeq rRNA Amplicon Sequencing protocol (Illumina, San Diego, CA, USA) with some modifications. PCR was performed in a final volume of 25 µL, which was set up with the following quantities: 5 µL of genomic DNA (10 ng/µL in H_2_O), 1× PCRBIO HiFi Buffer (PCR BIOSYSTEMS, Wayne, PA, USA) composed of 1 mM dNTPs and 3 mM MgCl_2_, 0.5 units of PCRBIO HiFi Polymerase (PCR BIOSYSTEMS, USA), and 0.2 μM of each primer. Cycling conditions followed an initial denaturation at 95 °C for 3 min, 25 cycles of 95 °C (30 s), 55 °C (30 s), 72 °C (30 s), final extension at 72 °C for 5 min, and hold at 4 °C. DNA amplicons were cleaned up by MagSi-NGSPREP Plus beads (Euroclone, Milan, Italy) and Illumina Nextera adaptor-primers were then used to barcode each sample through PCR as follows: 5 µL of cleaned up DNA amplicon, 1× PCRBIO HiFi Buffer (PCR BIOSYSTEMS, USA) composed of 1 mM dNTPs and 3 mM MgCl2, 0.5 units of PCRBIO HiFi Polymerase (PCR BIOSYSTEMS, USA), and 0.2 μM of each primer; cycling conditions followed an initial denaturation at 95 °C for 3 min, 8 cycles of 95 °C (30 s), 55 °C (30 s), 72 °C (30 s), final extension at 72 °C for 5 min, and hold at 4 °C. DNA amplicons were cleaned up by MagSi-NGSPREP Plus beads (Euroclone, Milan, Italy) and the final library was quantified by QuDye dsDNA HS Kit (Lumiprobe, Hannover, Germany). Samples were pooled and sequenced on an Illumina MiSeq™ platform according to the manufacturer’s specifications to generate paired-end reads of 2 × 300 nt.

The average number of raw reads obtained per sample was around 90k (min, 79k; max, 97k) paired-end (PE). After the trimming of low-quality reads by trimmomatic v0.40 (min length, 200 nt), the DADA2 algorithm was used with default parameters to process the prefiltered paired-end reads. Non-chimeric amplicons for each sample were used in downstream analysis to evaluate the bacterial diversity among the four samples, Gcyd Nap (17,427), Gcyd POL (16,373), Gcyd MP (20,438), and Gcyd IMTA (21,387).

The Naive Bayes classifier was trained on the V3-V4 region using the SILVA-138.1 16S database for the qiime2-2023.5 version [103]. Taxonomic assignment was performed applying this homemade pre-trained Naive Bayes classifier to the sequences obtained after the denoised filtering process. The metataxonomic analysis from raw DNA sequencing data was conducted on the Quantitative Insights Into Microbial Ecology (QIIME 2, Version: 2023.5) platform [104] by demultiplexing, quality filtering, chimera removal, taxonomic assignment, and both alpha and beta diversity analyses. With the use of R version 4.5.1 (Li, 2021) and Cairo graphics library [105], the taxonomic bar plot was generated. To evaluate the species diversity, three different indices were taken into account: the Chao 1 index [106] (Chao, 1984) is a qualitative species-based method (species richness in the sample is the number of ASVs) and the Shannon [107], Shannon and Weaver [108], and Simpson [109] indices estimated the quantitative species-based measures, which indicate the community diversity as species richness and evenness.

The full dataset of raw data was deposited in the SRA database (BioProject ID: PRJNA1370340; BioSample accessions: SAMN53439904 (GcydMP), SAMN53439905 (GcydIMTA), SAMN53439906 (GcydPOL), SAMN53439907) (GcydNAP).

## 4. Conclusions

The Mediterranean Sea is a renowned biodiversity hotspot, characterized by a high concentration of marine and endemic species within a relatively small area. Its exceptional richness is largely due to its unique geographical features and long history of isolation. It is also recognized as a rich yet relatively underexplored source of novel bioactive compounds derived from marine organisms, including sponges, algae, and microorganisms. These organisms produce unique secondary metabolites with promising applications in medicine and biotechnology. Despite its high biodiversity and extensive coastline, large portions of this marine environment remain understudied, offering significant opportunities for the discovery of new compounds with diverse pharmaceutical properties. The Mediterranean Sea represents a promising area for bioprospecting due also to its adaptation to unique environments. In fact, marine species in the Mediterranean have evolved specialized strategies to survive in their specific habitats, resulting in the production of unique chemical structures and bioactive secondary metabolites. The ecological success of *G. cydonium* is closely linked to its diverse internal microbial community. Classified as a high-microbial-abundance (HMA) sponge, it hosts a dense, specific, and complex consortium of microbes [110]. These microbial symbionts produce a range of bioactive metabolites that provide chemical defenses against disease, fouling, and predation [19]. Notably, cytotoxic compounds that disrupt the cellular cytoskeleton have been identified, highlighting potential applications in anti-cancer research [111,112]. The combination of ecological resilience and chemical diversity makes *G. cydonium* a prime candidate for bioprospecting and novel biotechnological applications. The results of this study confirm the role of the Mediterranean Sea as a biodiversity hotspot for sponge-associated microbial communities, highlighting the complexity and specificity of the microbiota associated with the sponge *G. cydonium*. In this exploratory study, we characterized the microbial communities of *G. cydonium* specimens collected from four southern Italian sites. Our analyses revealed a diverse assemblage dominated by seven major bacterial phyla, suggesting the presence of a preliminary core microbiota across sites. However, due to the single-specimen-per-site design, these findings should be interpreted cautiously and cannot be generalized as definitive site-specific patterns.

Metataxonomic analysis revealed a microbial composition dominated by seven major phyla, including Chloroflexota, Actinomycetota, and Proteobacteria, consistently present across all sampling sites, supporting the hypothesis of a species-specific core microbiota. However, quantitative variations among samples, along with differences in phylum presence or absence compared to previous studies, suggest that environmental, methodological, and biogeographical factors may significantly influence microbial community structure. The sample collected from Polignano a Mare appeared to exhibit the highest species richness and diversity, as indicated by the elevated Chao1, Shannon, and Simpson indices, along with the exclusive presence of seven bacterial genera and one class. Although these results suggested functional diversity and potential for bioprospecting, they are preliminary. Future studies with replicated sampling are needed to confirm whether the observed patterns reflect environmental influences, host specificity, or individual variability. However, it is important to emphasize that this study did not include seawater or sediment controls. Therefore, the microbial taxa identified cannot be conclusively considered stable symbionts of *G. cydonium*. Some observed taxa may represent transient environmental bacteria filtered from the surrounding seawater. Collection depths will also be taken into consideration to better distinguish host-specific microbial associations from environmental or depth-driven variability.

Many of these taxa are known for their ecological versatility and potential biotechnological applications, suggesting a complex and functionally diverse microbial ecosystem likely influenced by local environmental conditions [113,114]. Notably, any statements regarding the biotechnological or pharmacological potential of the microbial communities here identified are based on the literature reports of related taxa, rather than direct measurements from our samples; this will be the object of future studies integrating metagenomic, metatranscriptomic, or metabolomic analyses to validate the functional potential of *G. cydonium*-associated microbiota. In contrast, the sample from the IMTA system showed significantly lower diversity, indicating a potential impact of aquaculture conditions on the sponge microbial composition. The high microbial diversity and the presence of rare but potentially bioactive taxa underscore the potential of *G. cydonium* microbiota as a valuable resource for bioprospecting novel bioactive compounds. These findings further emphasize the importance of studying sponge-microbe interactions in the Mediterranean Sea, as well as the importance of habitat in shaping *G. cydonium* microbial communities and open promising avenues for future research aimed at understanding the functional interactions between sponges and their associated microbiota, with potential applications in marine biotechnology. Overall, this study provides an initial descriptive overview of *G. cydonium* microbiota in the Mediterranean and highlights the complexity and functional potential of sponge-associated microbial communities. Our results underscore the importance of conducting future research with multiple biological replicates to robustly assess the influence of habitat and environmental conditions on microbial community structure and function. Despite these limitations, our findings provide a useful baseline for generating hypotheses regarding sponge-associated microbial communities in the Mediterranean region.

## Figures and Tables

**Table 1:** Data on *Geodia cydonium* sampling sites.

Sites	Site ID	Coordinates	Depth (m)	Temperature (°C)	pH	Salinity (PSU)
**Mar Piccolo**	MP	40°28′ N 17°16′ E	2.5	25	8.3	39
**Mar Grande**	IMTA	40°26′ N 17°14′ E	3	23	8.1	38.5
**Polignano a Mare**	POL	40°59′ N 17°14′ E	2.5	24	8.2	38
**Secca delle Fumose**	NAP	40°49′ N 14°5′ E	20	23.9	8.3	38

## Data Availability

The full dataset of raw data was deposited in the SRA database (BioProject ID: PRJNA1370340).

## Supplementary Materials

The following supporting information can be downloaded at https://www.mdpi.com/article/10.3390/md24010002/s1, Figure S1: Krona plot representing the most abundant class for each sample of *G. cydonium* under analysis. Sample code: Gcyd_MP (Mar Piccolo), Gcyd_N (Gulf of Naples), Gcyd_P (Polignano a Mare), Gcyd_A (IMTA system); Figure S2: Phylogenetic tree illustrating the presence of bacteria and archaea in four samples of *G. cydonium* collected in the IMTA system, Mar Piccolo (MPIC), Secca delle Fumose (Naples, NAP), and Polignano a Mare (POL); Figure S3: Phylogenetic tree (reporting the distance between genera) illustrating the presence of bacteria and archaea in four samples of *G. cydonium* collected in IMTA system, Mar Piccolo (MPIC), Secca delle Fumose (Naples, NAP) and Polignano a Mare (POL); Table S1: ASVs (232) from *G. cydonium* collected in Secca delle Fumose (Gulf of Naples) with percentage of confidence ≥ 75%; Table S2:ASVs (300) from *G. cydonium* collected in Polignano a Mare with percentage of confidence ≥ 75%; Table S3: ASVs (136) from *G. cydonium* collected in Mar Piccolo with percentage of confidence ≥ 75%; Table S4: ASVs (148) from *G. cydonium* collected in Integrated Multi-Trophic Aquaculture (IMTA) system with percentage of confidence ≥ 75%.
