# Supplementary material for: Exploring the Microbial Reservoir of Geodia cydonium (Linnaeus, 1767): Insights into Site-Specific Diversity and Biotechnological Potential
Source: Mar Drugs. 2025 Dec 19;24(1):2. doi: 10.3390/md24010002 (PMC12842704; doi:10.3390/md24010002)

Table S1. ASVs (232) from *Geodia cydonium* collected in Secca delle Fumose (Gulf of Naples) with percentage of confidence ≥ 75%.

ASVs ID	1	Confidence	Domain	Phylum	Class	Order	Family	Genus	Species
c76990fc6d6e2a6e0a95e39f2b14a3bb	330	0.768	d__Bacteria	p__Actinobacteriota	c__Acidimicrobiia	o__Microtrichales	f__Microtrichaceae	g__Sva0996_marine_group	s__uncultured_bacterium
727ca8996ada5615b5a0c895ed6f8f7a	3	0.773	d__Bacteria	p__Verrucomicrobiota	c__Verrucomicrobiae	o__Verrucomicrobiales	f__DEV007	g__DEV007	s__uncultured_bacterium
3f7d3ee29d1237d3acbcf03f5faaf311	14	0.784	d__Bacteria	p__Proteobacteria	c__Gammaproteobacteria	o__JTB23	f__JTB23	g__JTB23	s__uncultured_bacterium
e380c0bc334f832a4dc4adbb782909d4	126	0.785	d__Bacteria	p__Poribacteria	c__Poribacteria	o__Poribacteria	f__Poribacteria	g__Poribacteria	s__uncultured_bacterium
8ace60eccca401ea254cf68ce9c6918b	3	0.785	d__Bacteria	p__Gemmatimonadota	c__BD2-11_terrestrial_group	o__BD2-11_terrestrial_group	f__BD2-11_terrestrial_group	g__BD2-11_terrestrial_group	s__uncultured_bacterium
6d9f4014486e8282646ad6b3c90ce2df	2	0.790	d__Bacteria	p__Firmicutes	c__Clostridia	o__Peptostreptococcales-Tissierellales			
3eb57d17be3537cbffb46a48d50db3d2	19	0.792	d__Bacteria	p__Actinobacteriota	c__Acidimicrobiia	o__Microtrichales	f__Microtrichaceae	g__Sva0996_marine_group	s__uncultured_bacterium
4d87d4a0fe3a217ca3647aea2187e8e6	29	0.795	d__Bacteria	p__Poribacteria	c__Poribacteria	o__Poribacteria	f__Poribacteria	g__Poribacteria	s__uncultured_bacterium
458b20ba69e5160ee718ba03913c6519	13	0.795	d__Bacteria	p__Gemmatimonadota	c__BD2-11_terrestrial_group	o__BD2-11_terrestrial_group	f__BD2-11_terrestrial_group	g__BD2-11_terrestrial_group	s__uncultured_bacterium
fde78c9b6d6c156ea5c8dc1bc9150adc	2	0.799	d__Bacteria	p__Poribacteria	c__Poribacteria	o__Poribacteria	f__Poribacteria	g__Poribacteria	s__uncultured_Poribacteria
6b1a3cf3e6cfce227010e7f34a9580a1	89	0.806	d__Bacteria	p__Actinobacteriota	c__Acidimicrobiia	o__Microtrichales	f__Microtrichaceae	g__Sva0996_marine_group	s__uncultured_bacterium
fdba3e43af94302a9b6a87163e792434	35	0.807	d__Bacteria	p__Poribacteria	c__Poribacteria	o__Poribacteria	f__Poribacteria	g__Poribacteria	s__Candidatus_Poribacteria
1d33e2f4ed86c33f30182612a07b085c	5	0.809	d__Bacteria	p__Proteobacteria	c__Gammaproteobacteria	o__JTB23	f__JTB23	g__JTB23	s__uncultured_proteobacterium

0a0b436ca2148b6db5f77637dc4622eb	2	0.811	d__Bacteria	p__Actinobacteriota	c__Acidimicrobiia	o__Microtrichales	f__Microtrichaceae	g__Sva0996_marine_group	
e4fdf0e7ebfaea088db23e6d94347ede	56	0.811	d__Bacteria	p__Proteobacteria	c__Gammaproteobacteria	o__Pseudomonadales	f__OM182_clade	g__OM182_clade	s__uncultured_gamma
9d8162a00edd64627d2894bd1d2ab1b9	2	0.813	d__Bacteria	p__Chloroflexi	c__Dehalococcoidia	o__SAR202_clade	f__SAR202_clade	g__SAR202_clade	
c0e06c13527a7df33b251bef74eb6413	44	0.816	d__Bacteria	p__Proteobacteria	c__Gammaproteobacteria	o__Pseudomonadales	f__Endozoicomonadaceae	g__Endozoicomonas	s__uncultured_Spongiobacter
de1394d56c658222057d2a040696a689	100	0.816	d__Bacteria	p__Poribacteria	c__Poribacteria	o__Poribacteria	f__Poribacteria	g__Poribacteria	s__Candidatus_Poribacteria
3ac5f9d7db1f63c09633b0f88301f68f	2	0.819	d__Bacteria	p__Myxococcota	c__bacteriap25	o__bacteriap25	f__bacteriap25	g__bacteriap25	s__uncultured_bacterium
063cce8658f9e598aa3a032f35ab3260	10	0.821	d__Bacteria	p__Chloroflexi	c__JG30-KF-CM66	o__JG30-KF-CM66	f__JG30-KF-CM66	g__JG30-KF-CM66	s__uncultured_bacterium
821d422ed366d6be46e0dd7e128db306	80	0.822	d__Bacteria	p__Actinobacteriota	c__Acidimicrobiia	o__Microtrichales	f__Microtrichaceae	g__Sva0996_marine_group	s__uncultured_bacterium
f3ff91dffb92b917adfa93bb232c802b	4	0.824	d__Bacteria	p__Proteobacteria	c__Gammaproteobacteria	o__Pseudomonadales	f__Halieaceae	g__OM60(NOR5)_clade	s__uncultured_bacterium
7ff06c1183f9d245523e7265be966be2	73	0.826	d__Bacteria	p__Chloroflexi	c__Dehalococcoidia	o__SAR202_clade	f__SAR202_clade	g__SAR202_clade	s__uncultured_Chloroflexi
0d234efa5aabd14c58a506f8748c6b02	81	0.828	d__Bacteria	p__Gemmatimonadota	c__BD2-11_terrestrial_group	o__BD2-11_terrestrial_group	f__BD2-11_terrestrial_group	g__BD2-11_terrestrial_group	s__uncultured_delta
8fa45acf2e34400e7436b7e195f126cb	101	0.829	d__Bacteria	p__Gemmatimonadota	c__BD2-11_terrestrial_group	o__BD2-11_terrestrial_group	f__BD2-11_terrestrial_group	g__BD2-11_terrestrial_group	s__uncultured_bacterium
c09dce33f9d1d385f5133a50994fdc44	3	0.832	d__Bacteria	p__Bdellovibrionota	c__Bdellovibrionia	o__Bdellovibrionales	f__Bdellovibrionaceae	g__Bdellovibrio	s__uncultured_bacterium
6c5d74165cd009d17fa96d1b8298b540	52	0.841	d__Bacteria	p__Bacteroidota	c__Rhodothermia	o__Rhodothermales	f__Rhodothermaceae	g__uncultured	s__uncultured_bacterium

d1b3ed6e18de40d28dcdcd4e739cfe4d	49	0.846	d__Bacteria	p__Actinobacteriota	c__Acidimicrobiia	o__Microtrichales	f__Microtrichaceae	g__Sva0996_marine_group	s__uncultured_actinobacterium
921dd46bd2764944eaccbbabaef7e319	38	0.847	d__Bacteria	p__Chloroflexi	c__Anaerolineae	o__SBR1031	f__A4b	g__A4b	s__uncultured_Chloroflexus
e1549dc223f478c1d8771b86f8ee8e6c	38	0.852	d__Bacteria	p__Poribacteria	c__Poribacteria	o__Poribacteria	f__Poribacteria	g__Poribacteria	s__Candidatus_Poribacteria
ae40de0c28ef2b2cdfe5c34e4eec3527	61	0.857	d__Bacteria	p__Chloroflexi	c__Dehalococcoidia	o__SAR202_clade	f__SAR202_clade	g__SAR202_clade	s__uncultured_Chloroflexi
afd2ae2940cff69ddd943d7efa4ace59	2	0.861	d__Bacteria	p__Actinobacteriota	c__Acidimicrobiia	o__Actinomarinales	f__uncultured	g__uncultured	
b504005fe3e7a782ec414f224b8ce9b6	6	0.861	d__Bacteria	p__Proteobacteria	c__Gammaproteobacteria	o__Pseudomonadales	f__KI89A_clade	g__KI89A_clade	s__uncultured_organism
effa34f80a6e5c8e6504ed2cf928ac3b	188	0.866	d__Bacteria	p__Actinobacteriota	c__Acidimicrobiia	o__Actinomarinales	f__uncultured	g__uncultured	s__uncultured_actinobacterium
3556f90a547bfed0cabe2d6eaf75691b	18	0.872	d__Bacteria	p__Acidobacteriota	c__Vicinamibacteria	o__Vicinamibacterales	f__uncultured	g__uncultured	s__uncultured_bacterium
9bbbe63f9b1101b0a25246dc85ed52cb	14	0.873	d__Bacteria	p__Cyanobacteria	c__Cyanobacteriia	o__Synechococcales	f__Cyanobiaceae	g__Synechococcus_CC9902	s__uncultured_bacterium
2e542a0ba846e7627d36bece76b9a0e7	9	0.878	d__Bacteria	p__Chloroflexi	c__Dehalococcoidia	o__SAR202_clade	f__SAR202_clade	g__SAR202_clade	s__uncultured_bacterium
73d5db3ecbd9cf6b965880af1c498e24	2	0.884	d__Bacteria	p__Poribacteria	c__Poribacteria	o__Poribacteria	f__Poribacteria	g__Poribacteria	s__Candidatus_Poribacteria
d37e374d3fe7396950f5f07b20362be6	4	0.885	d__Bacteria	p__Actinobacteriota	c__Acidimicrobiia	o__Microtrichales	f__Microtrichaceae	g__Sva0996_marine_group	
5ffe7e5cdb4e0ec3178eb51272b849a6	3	0.886	d__Bacteria	p__Poribacteria	c__Poribacteria	o__Poribacteria	f__Poribacteria	g__Poribacteria	
341e04c55cbdcb398cae9ec0e7f37629	56	0.887	d__Bacteria	p__Poribacteria	c__Poribacteria	o__Poribacteria	f__Poribacteria	g__Poribacteria	s__uncultured_Planctomycetales

f7bf4b1e175256ac49b4379afba5b8a5	37	0.887	d__Bacteria	p__Gemmatimonadota	c__BD2-11_terrestrial_group	o__BD2-11_terrestrial_group	f__BD2-11_terrestrial_group	g__BD2-11_terrestrial_group	s__uncultured_bacterium
97fb8ec1addf7f02c1b8ff7f49627563	131	0.888	d__Bacteria	p__Proteobacteria	c__Gammaproteobacteria	o__HOC36	f__HOC36	g__HOC36	s__uncultured_gamma
88b6d081caec18a35df2037295886130	23	0.891	d__Bacteria	p__Chloroflexi	c__Anaerolineae	o__SBR1031	f__A4b	g__A4b	s__uncultured_Chloroflexus
1941f130250e9a8de725e05bd43785b0	4	0.893	d__Bacteria	p__Chloroflexi	c__Dehalococcoidia	o__SAR202_clade	f__SAR202_clade	g__SAR202_clade	s__uncultured_bacterium
d705ae7eb53b75407900777b366c538e	3	0.895	d__Bacteria	p__Verrucomicrobiota	c__Verrucomicrobiae	o__Opitutales	f__Puniceicoccaceae	g__Cerasicoccus	s__uncultured_Verrucomicrobia
05570da352ff8bd242da1548e1db25fc	19	0.900	d__Bacteria	p__Chloroflexi	c__Dehalococcoidia	o__SAR202_clade	f__SAR202_clade	g__SAR202_clade	s__uncultured_bacterium
2ca9b028ca23b69271fbce9ecd8e6131	21	0.906	d__Bacteria	p__Chloroflexi	c__Dehalococcoidia	o__SAR202_clade	f__SAR202_clade	g__SAR202_clade	s__uncultured_SAR202
850d2ce3898718a7e42fbec1fd387689	32	0.907	d__Bacteria	p__Chloroflexi	c__Dehalococcoidia	o__SAR202_clade	f__SAR202_clade	g__SAR202_clade	s__Chloroflexi_bacterium
0afe9f04fb31196f59b6f0853098bcff	38	0.907	d__Bacteria	p__Actinobacteriota	c__Acidimicrobiia	o__Microtrichales	f__Microtrichaceae	g__Sva0996_marine_group	s__uncultured_bacterium
cf8c413052193af977287d9da18c4d39	109	0.909	d__Bacteria	p__Chloroflexi	c__Anaerolineae	o__SBR1031	f__A4b	g__A4b	s__uncultured_Chloroflexus
c52d3d9a6fd3cb21b0b9b6414ce26c25	2	0.913	d__Bacteria	p__Poribacteria	c__Poribacteria	o__Poribacteria	f__Poribacteria	g__Poribacteria	s__Candidatus_Poribacteria
e36dc2eb4a740c003e519e414c9ab09d	56	0.923	d__Bacteria	p__Proteobacteria	c__Alphaproteobacteria	o__Defluviicoccales	f__uncultured	g__uncultured	s__uncultured_bacterium
7002c643193e83563c91d1e6ffc44302	189	0.928	d__Bacteria	p__Gemmatimonadota	c__BD2-11_terrestrial_group	o__BD2-11_terrestrial_group	f__BD2-11_terrestrial_group	g__BD2-11_terrestrial_group	s__uncultured_bacterium
4d127c13f2ceb59102034367769b498d	4	0.932	d__Bacteria	p__Poribacteria	c__Poribacteria	o__Poribacteria	f__Poribacteria	g__Poribacteria	s__Candidatus_Poribacteria

e4fc3333123c849329d7675c66df815a	36	0.934	d__Bacteria	p__Proteobacteria	c__Alphaproteobacteria	o__Defluviicoccales	f__uncultured	g__uncultured	s__uncultured_bacterium
8db01b09fb18928f5694567c18dd8aa9	9	0.936	d__Bacteria	p__Proteobacteria	c__Gammaproteobacteria	o__UBA10353_marine_group	f__UBA10353_marine_group	g__UBA10353_marine_group	s__uncultured_bacterium
d20f87727008ff492ba0edea42e1591d	7	0.936	d__Bacteria	p__Chloroflexi	c__Dehalococcoidia	o__SAR202_clade	f__SAR202_clade	g__SAR202_clade	s__uncultured_SAR202
11fb7eeed4c7ca30e5792a6b9e9f08fc	30	0.937	d__Bacteria	p__Chloroflexi	c__Dehalococcoidia	o__SAR202_clade	f__SAR202_clade	g__SAR202_clade	s__uncultured_bacterium
a97eae53c423c75d5bf421e68fd34ccd	127	0.938	d__Bacteria	p__Chloroflexi	c__Dehalococcoidia	o__SAR202_clade	f__SAR202_clade	g__SAR202_clade	s__uncultured_SAR202
88bfc9a540c2d0a8250c4ce7c79b6981	3	0.938	d__Bacteria	p__Gemmatimonadota	c__BD2-11_terrestrial_group	o__BD2-11_terrestrial_group	f__BD2-11_terrestrial_group	g__BD2-11_terrestrial_group	s__uncultured_bacterium
a34e04ded1dfbe9bd0b011099ea96838	16	0.938	d__Bacteria	p__Proteobacteria	c__Gammaproteobacteria	o__UBA10353_marine_group	f__UBA10353_marine_group	g__UBA10353_marine_group	s__uncultured_bacterium
db978cb0c8d8a19da31fb5244c789b24	4	0.942	d__Bacteria	p__Patescibacteria	c__Parcubacteria	o__Candidatus_Kaiserbacteria	f__Candidatus_Kaiserbacteria	g__Candidatus_Kaiserbacteria	s__uncultured_bacterium
75640cc6c351913333abf8415a3af6e8	30	0.943	d__Bacteria	p__Poribacteria	c__Poribacteria	o__Poribacteria	f__Poribacteria	g__Poribacteria	s__Candidatus_Poribacteria
49a784104a72e1af36f7f4908333109a	19	0.951	d__Bacteria	p__Actinobacteriota	c__Acidimicrobiia	o__Microtrichales	f__Microtrichaceae	g__Sva0996_marine_group	s__uncultured_bacterium
bde571c1071072dab43ee92c07079ca7	25	0.955	d__Bacteria	p__Gemmatimonadota	c__BD2-11_terrestrial_group	o__BD2-11_terrestrial_group	f__BD2-11_terrestrial_group	g__BD2-11_terrestrial_group	s__uncultured_bacterium
f30e754cf92d3b27a58800ac56709758	13	0.956	d__Bacteria	p__Acidobacteriota	c__Vicinamibacteria	o__Vicinamibacterales	f__uncultured	g__uncultured	s__uncultured_bacterium
f9404661ca724985ec4de10ad921336f	26	0.959	d__Bacteria	p__Proteobacteria	c__Gammaproteobacteria	o__EPR3968-O8a-Bc78	f__EPR3968-O8a-Bc78	g__EPR3968-O8a-Bc78	s__uncultured_bacterium
23bc3fbd895cf4e58d0cfa0e5e2fd0db	70	0.960	d__Bacteria	p__Poribacteria	c__Poribacteria	o__Poribacteria	f__Poribacteria	g__Poribacteria	s__Candidatus_Poribacteria

cd7a605b7c7129c35c2b41a2c84441bb	34	0.962	d__Bacteria	p__Chloroflexi	c__Dehalococcoidia	o__S085	f__S085	g__S085	s__uncultured_bacterium
4f140d5050e43806116c905fad07ebc2	22	0.964	d__Bacteria	p__Gemmatimonadota	c__PAUC43f_marine_benthic_group	o__PAUC43f_marine_benthic_group	f__PAUC43f_marine_benthic_group	g__PAUC43f_marine_benthic_group	s__uncultured_bacterium
baba2af4aa14178d6590f6a27466f6b8	11	0.965	d__Bacteria	p__Chloroflexi	c__Dehalococcoidia	o__SAR202_clade	f__SAR202_clade	g__SAR202_clade	s__Chloroflexi_bacterium
ef16c0b009449f5620d9ed5fb3129d2d	5	0.967	d__Bacteria	p__Bdellovibrionota	c__Bdellovibrionia	o__Bdellovibrionales	f__Bdellovibrionaceae	g__Bdellovibrio	s__uncultured_Bdellovibrio
38f664340d867e0cc7f87dbbdc7dfcdb	1	0.968	d__Bacteria	p__Chloroflexi	c__Dehalococcoidia	o__SAR202_clade	f__SAR202_clade	g__SAR202_clade	
2c1cfcd31b173b430b31d311e9e66c72	18	0.969	d__Bacteria	p__Entotheonellaeota	c__Entotheonellia	o__Entotheonellales	f__Entotheonellaceae	g__Entotheonellaceae	s__uncultured_delta
afc4bc6ff20a7f81d11d4ab99b8b6bf9	36	0.969	d__Bacteria	p__Gemmatimonadota	c__BD2-11_terrestrial_group	o__BD2-11_terrestrial_group	f__BD2-11_terrestrial_group	g__BD2-11_terrestrial_group	s__uncultured_bacterium
5eb7df13fa54f17af51b009d1a747332	29	0.971	d__Bacteria	p__Chloroflexi	c__Dehalococcoidia	o__SAR202_clade	f__SAR202_clade	g__SAR202_clade	s__uncultured_bacterium
113197124816e7124d0a939782d2fd82	29	0.971	d__Bacteria	p__Chloroflexi	c__Dehalococcoidia	o__SAR202_clade	f__SAR202_clade	g__SAR202_clade	s__uncultured_bacterium
40c2b630e6829314f805e1d934951f02	116	0.972	d__Bacteria	p__Bacteroidota	c__Rhodothermia	o__Rhodothermales	f__Rhodothermaceae	g__uncultured	s__uncultured_bacterium
ab8ecc7d8382d653756f215910174e58	32	0.972	d__Bacteria	p__Chloroflexi	c__Dehalococcoidia	o__SAR202_clade	f__SAR202_clade	g__SAR202_clade	s__uncultured_bacterium
c1bc5c783371c11161d56cf7b40e3828	11	0.972	d__Bacteria	p__Proteobacteria	c__Gammaproteobacteria	o__Pseudomonadales	f__Pseudohongiellaceae	g__Pseudohongiella	s__uncultured_bacterium
83fdf376b04e0c73b219c821d5d1c1d5	14	0.975	d__Bacteria	p__Chloroflexi	c__Dehalococcoidia	o__S085	f__S085	g__S085	s__uncultured_bacterium
1fca9214ea64502cf023fa6372a8498d	22	0.976	d__Bacteria	p__Proteobacteria	c__Alphaproteobacteria	o__Kiloniellales	f__Kiloniellaceae	g__uncultured	s__uncultured_bacterium

bf7c48db5b7f62245f4e6507542ae90d	114	0.976	d__Bacteria	p__Actinobacteriota	c__Acidimicrobiia	o__Microtrichales	f__Microtrichaceae	g__Sva0996_marine_group	s__uncultured_bacterium
bc83829b014ac67af983e6922060089b	11	0.976	d__Bacteria	p__Proteobacteria	c__Gammaproteobacteria	o__Pseudomonadales	f__KI89A_clade	g__KI89A_clade	s__uncultured_bacterium
b559faa7f7ab3a1c1396b44b05fd57c9	2	0.977	d__Bacteria	p__Chloroflexi	c__Dehalococcoidia	o__SAR202_clade	f__SAR202_clade	g__SAR202_clade	
d4ae7081f0fb816b16a68049b1f5381d	42	0.978	d__Bacteria	p__Proteobacteria	c__Gammaproteobacteria	o__UBA10353_marine_group	f__UBA10353_marine_group	g__UBA10353_marine_group	s__uncultured_bacterium
318c3c6a14aed9d629284ae6c933bc18	1972	0.980	d__Bacteria	p__Actinobacteriota	c__Acidimicrobiia	o__Microtrichales	f__Microtrichaceae	g__Sva0996_marine_group	s__uncultured_bacterium
e68d370d47f1c36c771646ca1ad728ff	108	0.980	d__Bacteria	p__Actinobacteriota	c__Acidimicrobiia	o__Microtrichales	f__Microtrichaceae	g__Sva0996_marine_group	s__uncultured_actinobacterium
eaa31ab11fcae4e93a0ad0d9fb32bad0	2	0.981	d__Bacteria	p__Proteobacteria	c__Alphaproteobacteria	o__Rhizobiales	f__Stappiaceae	g__Pseudovibrio	
79ea44fbe6094230dcbb0e9f43858b6b	6	0.981	d__Bacteria	p__Chloroflexi	c__Dehalococcoidia	o__SAR202_clade	f__SAR202_clade	g__SAR202_clade	s__uncultured_Chloroflexus
503e05222b129911ae14bafeb5865b50	5	0.981	d__Bacteria	p__Acidobacteriota	c__Vicinamibacteria	o__Subgroup_9	f__Subgroup_9	g__Subgroup_9	s__uncultured_marine
51ea2cda562169e7b6ddc12ccf957ef3	227	0.984	d__Bacteria	p__Chloroflexi	c__Dehalococcoidia	o__SAR202_clade	f__SAR202_clade	g__SAR202_clade	s__uncultured_SAR202
5084222b7d2e21da3c64d11b63494bc4	24	0.984	d__Bacteria	p__Poribacteria	c__Poribacteria	o__Poribacteria	f__Poribacteria	g__Poribacteria	s__uncultured_bacterium
187bdf5cebe94820fee030f095b6e5a4	80	0.985	d__Bacteria	p__Chloroflexi	c__Anaerolineae	o__SBR1031	f__A4b	g__A4b	s__uncultured_Chloroflexi
ef2f6ba168e155cabcef8c6388aff9d3	10	0.987	d__Bacteria	p__Entotheonellaeota	c__Entotheonellia	o__Entotheonellales	f__Entotheonellaceae	g__Entotheonellaceae	s__uncultured_delta
208a5521acdba3f46f585a368a5dbf85	15	0.988	d__Bacteria	p__Poribacteria	c__Poribacteria	o__Poribacteria	f__Poribacteria	g__Poribacteria	s__Candidatus_Poribacteria

938eac81de414120530b016bbac17c2e	11	0.988	d__Bacteria	p__Entotheonellaeota	c__Entotheonellia	o__Entotheonellales	f__Entotheonellaceae	g__Entotheonellaceae	s__uncultured_delta
f25143a522db6e2446e4fae9dae92627	30	0.988	d__Bacteria	p__Acidobacteriota	c__Vicinamibacteria	o__Subgroup_9	f__Subgroup_9	g__Subgroup_9	s__uncultured_bacterium
a5f252c77ba9ec05e3210792c7ad8a69	262	0.989	d__Bacteria	p__Proteobacteria	c__Gammaproteobacteria	o__EPR3968-O8a-Bc78	f__EPR3968-O8a-Bc78	g__EPR3968-O8a-Bc78	s__uncultured_bacterium
3393c0a7c4f3203a44f1a8a6f85a845e	45	0.990	d__Bacteria	p__Chloroflexi	c__Anaerolineae	o__Caldilineales	f__Caldilineaceae	g__uncultured	s__uncultured_Caldilinea
546627ba3f2471e8f05ccfdb26c29d68	26	0.990	d__Bacteria	p__AncK6	c__AncK6	o__AncK6	f__AncK6	g__AncK6	s__uncultured_bacterium
34d6ace593c6ed2c515ceaf6be8dcf02	31	0.990	d__Bacteria	p__Poribacteria	c__Poribacteria	o__Poribacteria	f__Poribacteria	g__Poribacteria	s__Candidatus_Poribacteria
bd02a34a26b7aeb88f5f22841501a945	20	0.991	d__Bacteria	p__Chloroflexi	c__Dehalococcoidia	o__SAR202_clade	f__SAR202_clade	g__SAR202_clade	s__uncultured_bacterium
6641fd66d5fc27db144e7a93786136d4	8	0.992	d__Bacteria	p__Proteobacteria	c__Alphaproteobacteria	o__Puniceispirillales	f__EF100-94H03	g__EF100-94H03	s__uncultured_bacterium
34d9c97bf7481615ed6756eda28b8ff9	3	0.992	d__Bacteria	p__PAUC34f	c__PAUC34f	o__PAUC34f	f__PAUC34f	g__PAUC34f	s__uncultured_bacterium
1f2a17b74e6ce187cdf5d996d87c6314	38	0.993	d__Bacteria	p__Poribacteria	c__Poribacteria	o__Poribacteria	f__Poribacteria	g__Poribacteria	s__uncultured_Poribacteria
5a25f4609dbef9f0f9e30f0b3524fc53	4	0.993	d__Bacteria	p__Acidobacteriota	c__Thermoanaerobaculia	o__Thermoanaerobaculales	f__Thermoanaerobaculaceae	g__Subgroup_10	s__uncultured_bacterium
f8001f9bc1459e220983121817265230	52	0.993	d__Bacteria	p__Chloroflexi	c__Anaerolineae	o__Caldilineales	f__Caldilineaceae	g__uncultured	s__uncultured_Caldilinea
55cffc2e5fa49b3200e1e14b59a5b6ee	12	0.993	d__Bacteria	p__Chloroflexi	c__Dehalococcoidia	o__SAR202_clade	f__SAR202_clade	g__SAR202_clade	s__uncultured_bacterium
62b488835fc9b79e9788edc4e71b5330	30	0.993	d__Bacteria	p__Poribacteria	c__Poribacteria	o__Poribacteria	f__Poribacteria	g__Poribacteria	s__Candidatus_Poribacteria

b1794c0bbfa94ef953d263e969ffb7fd	25	0.993	d__Bacteria	p__Poribacteria	c__Poribacteria	o__Poribacteria	f__Poribacteria	g__Poribacteria	s__uncultured_bacterium
36d7c62a00f329e190ea639168d83c02	23	0.994	d__Bacteria	p__Actinobacteriota	c__Acidimicrobiia	o__Actinomarinales	f__uncultured	g__uncultured	
0bce9e193dd20323ff34751f8711d95e	1234	0.994	d__Bacteria	p__Poribacteria	c__Poribacteria	o__Poribacteria	f__Poribacteria	g__Poribacteria	s__uncultured_Poribacteria
b11e746055b3692569dac165ecf828e9	446	0.994	d__Bacteria	p__Actinobacteriota	c__Acidimicrobiia	o__Microtrichales	f__Microtrichaceae	g__Sva0996_marine_group	s__uncultured_bacterium
742ced2a280d3872e2b5442c2a777ca1	29	0.995	d__Bacteria	p__Cyanobacteria	c__Cyanobacteriia	o__Synechococcales	f__Cyanobiaceae	g__Cyanobium_PCC-6307	s__uncultured_bacterium
f74bf88be3c3fc3aa089270adfe9964d	21	0.995	d__Bacteria	p__Proteobacteria	c__Gammaproteobacteria	o__JTB23	f__JTB23	g__JTB23	s__uncultured_proteobacterium
daf6f27c6a3edd06d38f4f708479d161	2	0.996	d__Bacteria	p__Firmicutes	c__Clostridia	o__Lachnospirales	f__Lachnospiraceae		
674affa887eaac8b9213795e8a7df8ec	15	0.996	d__Bacteria	p__Acidobacteriota	c__Thermoanaerobaculia	o__Thermoanaerobaculales	f__Thermoanaerobaculaceae	g__Subgroup_10	s__uncultured_bacterium
3932d0fa1f1d94a503cc7e2650d396e7	165	0.996	d__Bacteria	p__Actinobacteriota	c__Acidimicrobiia	o__Actinomarinales	f__uncultured	g__uncultured	
e5ad1dabf32b619f429e1785386c5a2a	15	0.996	d__Bacteria	p__Chloroflexi	c__Anaerolineae	o__SBR1031	f__A4b	g__A4b	s__uncultured_Chloroflexus
b274a40882900f3fd9b7c15f6e8d8626	3	0.996	d__Bacteria	p__Chloroflexi	c__Dehalococcoidia	o__SAR202_clade	f__SAR202_clade	g__SAR202_clade	s__uncultured_bacterium
6b374149f88e0a57942b75e55edfb16f	10	0.996	d__Bacteria	p__Chloroflexi	c__Dehalococcoidia	o__SAR202_clade	f__SAR202_clade	g__SAR202_clade	s__uncultured_Chloroflexi
826f50c55c09c41981cdba8f2f2270dd	7	0.996	d__Bacteria	p__Poribacteria	c__Poribacteria	o__Poribacteria	f__Poribacteria	g__Poribacteria	s__uncultured_Planctomycetales
207f6a43addea0912666164a8ffae615	13	0.997	d__Bacteria	p__Proteobacteria	c__Gammaproteobacteria	o__Pseudomonadales	f__OM182_clade	g__OM182_clade	

f0f0515a647e20f3a63fa737a92c173f	28	0.997	d__Bacteria	p__Poribacteria	c__Poribacteria	o__Poribacteria	f__Poribacteria	g__Poribacteria	s__Candidatus_Poribacteria
f8c2578000e8d8783bdf16642fb4c705	35	0.997	d__Bacteria	p__Acidobacteriota	c__Subgroup_21	o__Subgroup_21	f__Subgroup_21	g__Subgroup_21	s__uncultured_bacterium
a9175c7f911cb499a3df1f8b39125204	28	0.998	d__Bacteria	p__Acidobacteriota	c__Vicinamibacteria	o__Subgroup_9	f__Subgroup_9	g__Subgroup_9	s__uncultured_bacterium
087910d4f98c7cb5874298c6adb2b462	48	0.998	d__Bacteria	p__Proteobacteria	c__Gammaproteobacteria	o__JTB23	f__JTB23	g__JTB23	s__uncultured_proteobacterium
20697a127d8ad80b778f8255215e80f9	49	0.998	d__Bacteria	p__Entotheonellaeota	c__Entotheonellia	o__Entotheonellales	f__Entotheonellaceae	g__Entotheonellaceae	s__uncultured_delta
5e81072a668add269663058523236f51	31	0.998	d__Bacteria	p__Actinobacteriota	c__Acidimicrobiia	o__Microtrichales	f__Microtrichaceae	g__Sva0996_marine_group	
9b82d97f794db33d8f2e90e32d9b384b	189	0.998	d__Bacteria	p__Actinobacteriota	c__Acidimicrobiia	o__Microtrichales	f__Microtrichaceae	g__Sva0996_marine_group	
6c809937e62b7b9e3d9680dde8514458	10	0.998	d__Bacteria	p__Proteobacteria	c__Gammaproteobacteria	o__pItb-vmat-80	f__pItb-vmat-80	g__pItb-vmat-80	s__uncultured_bacterium
3ec1e43e77fd669fccaa8c70ac9fdb22	18	0.998	d__Bacteria	p__Proteobacteria	c__Gammaproteobacteria	o__pItb-vmat-80	f__pItb-vmat-80	g__pItb-vmat-80	s__uncultured_bacterium
b3149a595fd8a22583e12cea6d9ac6bf	12	0.998	d__Bacteria	p__Actinobacteriota	c__Acidimicrobiia	o__Microtrichales	f__Microtrichaceae	g__Sva0996_marine_group	
052ace1004921da16db003cbafc2cfee	81	0.998	d__Bacteria	p__Proteobacteria	c__Gammaproteobacteria	o__UBA10353_marine_group	f__UBA10353_marine_group	g__UBA10353_marine_group	s__uncultured_marine
20c396f32ec75bfc16289328f8f837db	5	0.998	d__Bacteria	p__Gemmatimonadota	c__PAUC43f_marine_benthic_group	o__PAUC43f_marine_benthic_group	f__PAUC43f_marine_benthic_group	g__PAUC43f_marine_benthic_group	s__uncultured_bacterium
af10a20a19fb941c5021ee854d0b68e3	118	0.998	d__Bacteria	p__Proteobacteria	c__Gammaproteobacteria	o__JTB23	f__JTB23	g__JTB23	
fd76d5d72600eba4259576336555db00	2	0.998	d__Bacteria	p__Poribacteria	c__Poribacteria	o__Poribacteria	f__Poribacteria	g__Poribacteria	s__Candidatus_Poribacteria

39ad9d5e1d861b8925bffb1082e586f5	154	0.998	d__Bacteria	p__Acidobacteriota	c__Vicinamibacteria	o__Subgroup_9	f__Subgroup_9	g__Subgroup_9	s__uncultured_bacterium
0c4bf83ed62712a7120b251d7a19d450	15	0.999	d__Bacteria	p__Actinobacteriota	c__Acidimicrobiia	o__Actinomarinales	f__uncultured	g__uncultured	
106eddd65c6daf26e83648ebf0fe4d50	5	0.999	d__Bacteria	p__Chloroflexi	c__Dehalococcoidia	o__SAR202_clade	f__SAR202_clade	g__SAR202_clade	
014e17b29a1c01cae4ff773cca164a1c	168	0.999	d__Bacteria	p__Actinobacteriota	c__Acidimicrobiia	o__Microtrichales	f__Microtrichaceae	g__Sva0996_marine_group	
1f5b9d32f3040bcb7d88190d889f79de	36	0.999	d__Bacteria	p__Chloroflexi	c__Dehalococcoidia	o__SAR202_clade	f__SAR202_clade	g__SAR202_clade	s__uncultured_bacterium
eaab7be1e91b7a406d241bad2c8aacf5	98	0.999	d__Bacteria	p__Actinobacteriota	c__Acidimicrobiia	o__Actinomarinales	f__uncultured	g__uncultured	s__uncultured_actinobacterium
b5b0f076cba7d03c4f90d32eec106ab6	22	0.999	d__Bacteria	p__Gemmatimonadota	c__PAUC43f_marine_benthic_group	o__PAUC43f_marine_benthic_group	f__PAUC43f_marine_benthic_group	g__PAUC43f_marine_benthic_group	s__uncultured_bacterium
f72871b2770cbfacc1d095d45ff7b3e4	456	0.999	d__Bacteria	p__Actinobacteriota	c__Acidimicrobiia	o__Microtrichales	f__Microtrichaceae	g__Sva0996_marine_group	
09063d8e97ac7e2f59c58d9874c1d6a9	23	0.999	d__Bacteria	p__Poribacteria	c__Poribacteria	o__Poribacteria	f__Poribacteria	g__Poribacteria	s__uncultured_Planctomycetales
b936c98dae871c62c562ca5d09bb1809	11	0.999	d__Bacteria	p__Proteobacteria	c__Alphaproteobacteria	o__Rhodospirillales	f__Magnetospiraceae	g__uncultured	s__uncultured_bacterium
fb2927eba4dc540fe02433642782f9b6	26	0.999	d__Bacteria	p__Proteobacteria	c__Gammaproteobacteria	o__Pseudomonadales	f__KI89A_clade	g__KI89A_clade	s__uncultured_bacterium
d77f6bd395b7c11f9ba2aa8720131dc9	6	0.999	d__Bacteria	p__Myxococcota	c__bacteriap25	o__bacteriap25	f__bacteriap25	g__bacteriap25	s__uncultured_bacterium
85383c420d3feb0185f0a45a5bb830d2	1028	0.999	d__Bacteria	p__Poribacteria	c__Poribacteria	o__Poribacteria	f__Poribacteria	g__Poribacteria	s__Candidatus_Poribacteria
b1fdd817d2683c3dd46dca47df3ef74e	3	0.999	d__Bacteria	p__Poribacteria	c__Poribacteria	o__Poribacteria	f__Poribacteria	g__Poribacteria	s__Candidatus_Poribacteria

1951a8ea490c809627cea851ef09deec	26	0.999	d__Bacteria	p__Proteobacteria	c__Gammaproteobacteria	o__Pseudomonadales	f__KI89A_clade	g__KI89A_clade	s__uncultured_bacterium
56f3182a7fcad82a6903ef9d4da46423	17	0.999	d__Bacteria	p__Spirochaetota	c__Spirochaetia	o__Spirochaetales	f__Spirochaetaceae	g__Spirochaeta	s__uncultured_bacterium
c3a426b285ac1d04ef66f01d59891e1f	39	0.999	d__Bacteria	p__Acidobacteriota	c__Acidobacteriae	o__PAUC26f	f__PAUC26f	g__PAUC26f	s__uncultured_bacterium
4c3de93f163a4b49e00aec37b60294de	3	1.000	d__Bacteria	p__Poribacteria	c__Poribacteria	o__Poribacteria	f__Poribacteria	g__Poribacteria	s__Candidatus_Poribacteria
0c571b8b2e756a5e16ed6dd219d52969	15	1.000	d__Bacteria	p__Gemmatimonadota	c__PAUC43f_marine_benthic_group	o__PAUC43f_marine_benthic_group	f__PAUC43f_marine_benthic_group	g__PAUC43f_marine_benthic_group	s__uncultured_bacterium
a087e2859a5bca997c5d6595c0ac3280	35	1.000	d__Bacteria	p__Cyanobacteria	c__Cyanobacteriia	o__Synechococcales	f__Cyanobiaceae	g__Synechococcus_CC9902	
bd2eb86c8d95cb19ebb6932471b3a8f7	15	1.000	d__Bacteria	p__Poribacteria	c__Poribacteria	o__Poribacteria	f__Poribacteria	g__Poribacteria	s__uncultured_Poribacteria
efe90242338cd5d74ffbd877b4129b04	47	1.000	d__Bacteria	p__Proteobacteria	c__Gammaproteobacteria	o__Steroidobacterales	f__Woeseiaceae	g__JTB255_marine_benthic_group	s__uncultured_bacterium
9a3dee015b81ba479f9297af9a452c19	34	1.000	d__Bacteria	p__Chloroflexi	c__Dehalococcoidia	o__SAR202_clade	f__SAR202_clade	g__SAR202_clade	s__uncultured_deep-sea
b7e446291a0137f5d957dd7a8ed8c31d	9	1.000	d__Bacteria	p__Actinobacteriota	c__Actinobacteria	o__Micrococcales	f__Microbacteriaceae	g__ML602J-51	s__uncultured_organism
a7febd04d2c878e674539ac24d0c5c03	42	1.000	d__Bacteria	p__Chloroflexi	c__Dehalococcoidia	o__SAR202_clade	f__SAR202_clade	g__SAR202_clade	s__uncultured_deep-sea
e9137e128559135e1a3cf6b20f59059b	16	1.000	d__Bacteria	p__Proteobacteria	c__Gammaproteobacteria	o__Pseudomonadales	f__KI89A_clade	g__KI89A_clade	s__uncultured_bacterium
481ae635013df657661f0f25d0e5ee28	6	1.000	d__Bacteria	p__Proteobacteria	c__Gammaproteobacteria	o__UBA10353_marine_group	f__UBA10353_marine_group	g__UBA10353_marine_group	s__uncultured_marine
28b2c62b073a82585734473c306046c0	32	1.000	d__Bacteria	p__Poribacteria	c__Poribacteria	o__Poribacteria	f__Poribacteria	g__Poribacteria	s__Candidatus_Poribacteria

6119b027aa2f40502247c5a1a0363f02	41	1.000	d__Bacteria	p__Dadabacteria	c__Dadabacteriia	o__Dadabacteriales	f__Dadabacteriales	g__Dadabacteriales	s__uncultured_delta
ab6151de482049167acb71a2969446f5	59	1.000	d__Bacteria	p__Proteobacteria	c__Alphaproteobacteria	o__Defluviicoccales	f__uncultured	g__uncultured	s__uncultured_bacterium
ba2be41282c68bc0ca3cb31ba0f17fb1	191	1.000	d__Bacteria	p__Actinobacteriota	c__Acidimicrobiia	o__Actinomarinales	f__uncultured	g__uncultured	s__uncultured_actinobacterium
d5127d3631a165fa06b942e8f1726597	12	1.000	d__Bacteria	p__PAUC34f	c__PAUC34f	o__PAUC34f	f__PAUC34f	g__PAUC34f	s__uncultured_bacterium
308b149b607d495174541fb08ba2c10c	163	1.000	d__Bacteria	p__Proteobacteria	c__Gammaproteobacteria	o__Steroidobacterales	f__Woeseiaceae	g__JTB255_marine_benthic_group	s__uncultured_bacterium
e806ba571b3ee96d8244857bcab30ab6	187	1.000	d__Bacteria	p__Acidobacteriota	c__Thermoanaerobaculia	o__Thermoanaerobaculales	f__Thermoanaerobaculaceae	g__Subgroup_10	s__uncultured_bacterium
bdff7c37ad93e1a510c96f1e7e81e5f5	10	1.000	d__Bacteria	p__PAUC34f	c__PAUC34f	o__PAUC34f	f__PAUC34f	g__PAUC34f	s__uncultured_bacterium
67d32d9ad1b8d9919894a6b554d0742f	653	1.000	d__Bacteria	p__Acidobacteriota	c__Thermoanaerobaculia	o__Thermoanaerobaculales	f__Thermoanaerobaculaceae	g__Subgroup_10	s__uncultured_bacterium
2096ec2419cb280b4714757273cba800	33	1.000	d__Bacteria	p__PAUC34f	c__PAUC34f	o__PAUC34f	f__PAUC34f	g__PAUC34f	s__uncultured_bacterium
d7b0f8bc32312b880a77e736f398985a	2	1.000	d__Bacteria	p__Chloroflexi	c__Dehalococcoidia	o__SAR202_clade	f__SAR202_clade	g__SAR202_clade	
83b7ca6de6e993042cd6b5eb2a3cc3c8	222	1.000	d__Bacteria	p__Proteobacteria	c__Gammaproteobacteria	o__Pseudomonadales	f__KI89A_clade	g__KI89A_clade	s__uncultured_bacterium
7d9096f84f149aea452c5c50517384b7	14	1.000	d__Bacteria	p__Proteobacteria	c__Gammaproteobacteria	o__UBA10353_marine_group	f__UBA10353_marine_group	g__UBA10353_marine_group	
628b187675e0eadee63e464e413f574f	2	1.000	d__Bacteria	p__Proteobacteria	c__Alphaproteobacteria	o__Puniceispirillales	f__SAR116_clade	g__SAR116_clade	s__uncultured_bacterium
f829766bfb6ed4c8e50e44ad3fd7d070	26	1.000	d__Bacteria	p__Nitrospirota	c__Nitrospiria	o__Nitrospirales	f__Nitrospiraceae	g__Nitrospira	s__uncultured_bacterium

5a8c1c9db3c3545353c94d4f478ef15d	613	1.000	d__Bacteria	p__Dadabacteria	c__Dadabacteriia	o__Dadabacteriales	f__Dadabacteriales	g__Dadabacteriales	s__uncultured_delta
0d8f666c087c9f2d3f828cf2f2316539	24	1.000	d__Bacteria	p__Proteobacteria	c__Alphaproteobacteria	o__Puniceispirillales	f__Puniceispirillales_Incertae_Sedis	g__Constrictibacter	s__uncultured_bacterium
8b01ec84b4df797948c0fcdf079ebb32	5	1.000	d__Bacteria	p__Nitrospirota	c__Nitrospiria	o__Nitrospirales	f__Nitrospiraceae	g__Nitrospira	s__uncultured_bacterium
c3a8634a557d0c8a1f63fc6cc4574626	37	1.000	d__Bacteria	p__Acidobacteriota	c__Thermoanaerobaculia	o__Thermoanaerobaculales	f__Thermoanaerobaculaceae	g__Subgroup_10	s__uncultured_bacterium
045cab2b76f086437e3400b7cbb17c84	9	1.000	d__Bacteria	p__Proteobacteria	c__Gammaproteobacteria	o__Pseudomonadales	f__KI89A_clade	g__KI89A_clade	s__uncultured_bacterium
1ed19b4056ece44e5b92d5c90b7ef74	14	1.000	d__Bacteria	p__Chloroflexi	c__TK17	o__TK17	f__TK17	g__TK17	s__uncultured_Chloroflexi
aa0bb46f8879fdd1efd50ce3d7859c75	25	1.000	d__Bacteria	p__Proteobacteria	c__Gammaproteobacteria	o__Pseudomonadales	f__KI89A_clade	g__KI89A_clade	s__uncultured_bacterium
d6a79416c0b360cb1c7da2b29c779121	119	1.000	d__Bacteria	p__Verrucomicrobiota	c__Verrucomicrobiae	o__Opitutales	f__Puniceicoccaceae	g__Cerasicoccus	s__uncultured_Verrucomicrobia
e1731bd5d1e591d09317ee220ce0d3e7	127	1.000	d__Bacteria	p__Chloroflexi	c__Dehalococcoidia	o__SAR202_clade	f__SAR202_clade	g__SAR202_clade	
c64130d21eaf070c2b730623dd098bbf	31	1.000	d__Bacteria	p__PAUC34f	c__PAUC34f	o__PAUC34f	f__PAUC34f	g__PAUC34f	s__uncultured_bacterium
876e2f7e37b7db45be54a9befdb1a363	39	1.000	d__Bacteria	p__Poribacteria	c__Poribacteria	o__Poribacteria	f__Poribacteria	g__Poribacteria	s__uncultured_Poribacteria
09dfb5a421717b6ad0a71a4a5ca61392	3	1.000	d__Bacteria	p__Bdellovibrionota	c__Bdellovibrionia	o__Bdellovibrionales	f__Bdellovibrionaceae	g__Bdellovibrio	s__uncultured_delta
f5b28b630059b6f704b3926f9ac71356	195	1.000	d__Bacteria	p__Proteobacteria	c__Gammaproteobacteria	o__Steroidobacterales	f__Woeseiaceae	g__JTB255_marine_benthic_group	s__uncultured_bacterium
b85f1126d6929768b78d7f687e715ee8	17	1.000	d__Bacteria	p__AncK6	c__AncK6	o__AncK6	f__AncK6	g__AncK6	s__uncultured_bacterium

d7c5f473f6373dc98492829af4241f42	4	1.000	d__Bacteria	p__Chloroflexi	c__Dehalococcoidia	o__SAR202_clade	f__SAR202_clade	g__SAR202_clade	
315ca649e9266db51e286539b0cc4318	194	1.000	d__Bacteria	p__Chloroflexi	c__Anaerolineae	o__Caldilineales	f__Caldilineaceae	g__uncultured	s__uncultured_Chloroflexus
017ff74128eb105f52148b74a2a5c17d	141	1.000	d__Bacteria	p__Poribacteria	c__Poribacteria	o__Poribacteria	f__Poribacteria	g__Poribacteria	s__Candidatus_Poribacteria
8e2b88fa6680528734b9570ffd855be8	164	1.000	d__Bacteria	p__Chloroflexi	c__Anaerolineae	o__Caldilineales	f__Caldilineaceae	g__uncultured	s__uncultured_Chloroflexus
eb20dcaed0906e2f30f3c6ac5c0e27b2	8	1.000	d__Bacteria	p__Chloroflexi	c__Anaerolineae	o__SBR1031	f__A4b	g__A4b	s__uncultured_Thermomicrobium
712869e9bf8bcbe9e2e4d535a1e3db0b	9	1.000	d__Bacteria	p__Proteobacteria	c__Gammaproteobacteria	o__Pseudomonadales	f__KI89A_clade	g__KI89A_clade	
7acea8299e26f7d51b0953e29ed4a686	44	1.000	d__Bacteria	p__Chloroflexi	c__Anaerolineae	o__Caldilineales	f__Caldilineaceae	g__uncultured	s__uncultured_Chloroflexus
80214b75450e4d53893b0f57d371a6fc	13	1.000	d__Bacteria	p__Chloroflexi	c__Dehalococcoidia	o__SAR202_clade	f__SAR202_clade	g__SAR202_clade	
7be2632751b6fce52f846e031c9b99b4	161	1.000	d__Bacteria	p__Acidobacteriota	c__Acidobacteriae	o__PAUC26f	f__PAUC26f	g__PAUC26f	s__uncultured_bacterium
863382f82e439c69fba5f72df01794b8	35	1.000	d__Bacteria	p__Acidobacteriota	c__Vicinamibacteria	o__Vicinamibacterales	f__uncultured	g__uncultured	
89674a06a0c850641379a5bb3f7de0e9	20	1.000	d__Bacteria	p__Chloroflexi	c__Dehalococcoidia	o__SAR202_clade	f__SAR202_clade	g__SAR202_clade	
59f066cbee429608952707821137649e	4	1.000	d__Bacteria	p__Proteobacteria	c__Gammaproteobacteria	o__Pseudomonadales	f__KI89A_clade	g__KI89A_clade	
00862b496aebdf69e1096a24c0d16167	61	1.000	d__Bacteria	p__Poribacteria	c__Poribacteria	o__Poribacteria	f__Poribacteria	g__Poribacteria	s__Candidatus_Poribacteria
85f72f312b6ced96a68e9cce2fdaf2c9	101	1.000	d__Bacteria	p__Acidobacteriota	c__Acidobacteriae	o__PAUC26f	f__PAUC26f	g__PAUC26f	s__uncultured_bacterium

d9fba8f27971c65a9b820cf7ffa0c1af	24	1.000	d__Bacteria	p__Proteobacteria	c__Gammaproteobacteria	o__Pseudomonadales	f__KI89A_clade	g__KI89A_clade	
57f45003e7532e653dbc2fd5cf9144ef	11	1.000	d__Bacteria	p__Proteobacteria	c__Gammaproteobacteria	o__Pseudomonadales	f__KI89A_clade	g__KI89A_clade	
aa813950cf08f88f5c35ee7ea0cb46e7	7	1.000	d__Bacteria	p__PAUC34f	c__PAUC34f	o__PAUC34f	f__PAUC34f	g__PAUC34f	s__uncultured_bacterium
07a3c11e8b367b784f92d6870fc6e17f	152	1.000	d__Bacteria	p__Poribacteria	c__Poribacteria	o__Poribacteria	f__Poribacteria	g__Poribacteria	s__Candidatus_Poribacteria
36efecc5394efd07b3541546b4edd7f7	38	1.000	d__Bacteria	p__Deinococcota	c__Deinococci	o__Deinococcales	f__Trueperaceae	g__Truepera	s__uncultured_Truepera
fbd37fe5f06be8faa560ba409c36faee	8	1.000	d__Bacteria	p__Chloroflexi	c__Dehalococcoidia	o__SAR202_clade	f__SAR202_clade	g__SAR202_clade	
4984dda37b133126f0e2be49a4ab09c2	19	1.000	d__Bacteria	p__PAUC34f	c__PAUC34f	o__PAUC34f	f__PAUC34f	g__PAUC34f	s__uncultured_Deferribacteres
11fe107d7fb62f0947861e1dfb050164	3	1.000	d__Bacteria	p__NB1-j	c__NB1-j	o__NB1-j	f__NB1-j	g__NB1-j	
5926f5ae0f8a1862a4ffe06f48cbbb29	20	1.000	d__Bacteria	p__Proteobacteria	c__Gammaproteobacteria	o__Pseudomonadales	f__OM182_clade	g__OM182_clade	
04d07512c2ed4c1f87972caf6fbf17aa	6	1.000	d__Bacteria	p__Poribacteria	c__Poribacteria	o__Poribacteria	f__Poribacteria	g__Poribacteria	s__Candidatus_Poribacteria
f305db5fd41041afac34905c2eac67ad	6	1.000	d__Bacteria	p__Poribacteria	c__Poribacteria	o__Poribacteria	f__Poribacteria	g__Poribacteria	s__Candidatus_Poribacteria
707fe796f8fa5c4d105f98efb633be38	309	1.000	d__Bacteria	p__Poribacteria	c__Poribacteria	o__Poribacteria	f__Poribacteria	g__Poribacteria	s__Candidatus_Poribacteria
2eee99740ae0d74520aa4d63d886772b	15	1.000	d__Bacteria	p__Chloroflexi	c__Anaerolineae	o__SBR1031	f__A4b	g__A4b	
88aac3ca771c1fe8266666981475b080	4	1.000	d__Bacteria	p__Chloroflexi	c__Anaerolineae	o__SBR1031	f__A4b	g__A4b	

374255d4f186035aeeab8644d17324ac	352	1.000	d__Bacteria	p__Poribacteria	c__Poribacteria	o__Poribacteria	f__Poribacteria	g__Poribacteria	s__Candidatus_Poribacteria
2dd77cc0448701cd23bc91ae10efe769	66	1.000	d__Bacteria	p__Chloroflexi	c__Anaerolineae	o__Caldilineales	f__Caldilineaceae	g__uncultured	s__uncultured_Chloroflexus
5a2e587ac3fc8064fe70bb3592c72f69	64	1.000	d__Bacteria	p__Acidobacteriota	c__Subgroup_11	o__Subgroup_11	f__Subgroup_11	g__Subgroup_11	s__uncultured_bacterium
32b93a1cb4ccccd33770618aba683e99	2	1.000	d__Bacteria	p__Proteobacteria	c__Alphaproteobacteria	o__Rhodospirillales	f__AEGEAN-169_marine_group	g__AEGEAN-169_marine_group	
958ecd59951d0ee9fffe7e5f4705b9e7	42	1.000	d__Bacteria	p__Gemmatimonadota	c__BD2-11_terrestrial_group	o__BD2-11_terrestrial_group	f__BD2-11_terrestrial_group	g__BD2-11_terrestrial_group	
5af73ce6f1540dcb0ad2ca4b79de56e4	39	1.000	d__Bacteria	p__Gemmatimonadota	c__BD2-11_terrestrial_group	o__BD2-11_terrestrial_group	f__BD2-11_terrestrial_group	g__BD2-11_terrestrial_group	
581a66aaa68ddedda604b324cb2fb586	111	1.000	d__Bacteria	p__Gemmatimonadota	c__BD2-11_terrestrial_group	o__BD2-11_terrestrial_group	f__BD2-11_terrestrial_group	g__BD2-11_terrestrial_group	
8c8f443da7e316ee1dd74a542e350c08	8	1.000	d__Bacteria	p__Acidobacteriota	c__Vicinamibacteria	o__Subgroup_9	f__Subgroup_9	g__Subgroup_9	
204039ee539ee4803e113e869ad8d1ec	282	1.000	d__Bacteria	p__Poribacteria	c__Poribacteria	o__Poribacteria	f__Poribacteria	g__Poribacteria	

Table S2. ASVs (300) from *Geodia cydonium* collected in Polignano a Mare with percentage of confidence ≥ 75%.

ASVs ID	2	Confidence	Domain	Phylum	Class	Order	Family	Genus	Species
3cf57f1e30a8605ee44d4ebbb056ccc1	38	0.752	d__Bacteria	p__Chloroflexi	c__Dehalococcoidia	o__SAR202_clade	f__SAR202_clade	g__SAR202_clade	s__uncultured_bacterium
861629e9d1339c12ef23c208d2ec0df2	19	0.761	d__Bacteria	p__Proteobacteria	c__Gammaproteobacteria	o__D90	f__D90	g__D90	s__uncultured_gamma
f8d83d5055ed42b8e756f5654d2bcb14	11	0.765	d__Bacteria	p__Patescibacteria	c__Parcubacteria	o__Candidatus_Spechtbacteria	f__Candidatus_Spechtbacteria	g__Candidatus_Spechtbacteria	s__uncultured_bacterium
c76990fc6d6e2a6e0a95e39f2b14a3bb	344	0.768	d__Bacteria	p__Actinobacteriota	c__Acidimicrobiia	o__Microtrichales	f__Microtrichaceae	g__Sva0996_marine_group	s__uncultured_bacterium
42591e1f790e73e2d23bde76b4f2d5b9	4	0.772	d__Bacteria	p__Acidobacteriota	c__Acidobacteriae	o__PAUC26f	f__PAUC26f	g__PAUC26f	s__uncultured_bacterium
2845e3a98b1e8b0c4933259657c63aa2	10	0.774	d__Bacteria	p__Acidobacteriota	c__Vicinamibacteria	o__Vicinamibacterales	f__uncultured	g__uncultured	s__uncultured_bacterium
203c6df9b41c402cd1c0c8b96533b9b4	21	0.780	d__Bacteria	p__Proteobacteria	c__Alphaproteobacteria	o__AT-s3-44	f__AT-s3-44	g__AT-s3-44	s__uncultured_bacterium
f88c829d9fd92ff23377d1d9fbcdc2eb	6	0.783	d__Bacteria	p__Chloroflexi	c__Dehalococcoidia	o__SAR202_clade	f__SAR202_clade	g__SAR202_clade	s__uncultured_marine
3f7d3ee29d1237d3acbcf03f5faaf311	30	0.784	d__Bacteria	p__Proteobacteria	c__Gammaproteobacteria	o__JTB23	f__JTB23	g__JTB23	s__uncultured_bacterium
e380c0bc334f832a4dc4adbb782909d4	11	0.785	d__Bacteria	p__Poribacteria	c__Poribacteria	o__Poribacteria	f__Poribacteria	g__Poribacteria	s__uncultured_bacterium
017537ff13e06dbc75739e1946cc2dc4	47	0.794	d__Bacteria	p__Chloroflexi	c__Dehalococcoidia	o__SAR202_clade	f__SAR202_clade	g__SAR202_clade	s__uncultured_bacterium
7e5a1ec6e5ed92d3fe75454c21054e46	2	0.795	d__Bacteria	p__Chloroflexi	c__Dehalococcoidia	o__SAR202_clade	f__SAR202_clade	g__SAR202_clade	s__uncultured_bacterium
bccd7d15a90cb21614b22531ede041b3	6	0.798	d__Bacteria	p__Proteobacteria	c__Gammaproteobacteria	o__Pseudomonadales	f__KI89A_clade	g__KI89A_clade	s__uncultured_bacterium

c5d6943cc3e9953d5e97a43830b8f004	3	0.803	d__Bacteria	p__Gemmatimonadota	c__BD2-11_terrestrial_group	o__BD2-11_terrestrial_group	f__BD2-11_terrestrial_group	g__BD2-11_terrestrial_group	s__uncultured_bacterium
a7428f7c981c79d7e08152d946227625	15	0.803	d__Bacteria	p__Nitrospinota	c__P9X2b3D02	o__P9X2b3D02	f__P9X2b3D02	g__P9X2b3D02	s__uncultured_bacterium
6b1a3cf3e6cfce227010e7f34a9580a1	38	0.806	d__Bacteria	p__Actinobacteriota	c__Acidimicrobiia	o__Microtrichales	f__Microtrichaceae	g__Sva0996_marine_group	s__uncultured_bacterium
a83c77cc2d63ef49e17e2faa10fc8246	113	0.806	d__Bacteria	p__Chloroflexi	c__Dehalococcoidia	o__SAR202_clade	f__SAR202_clade	g__SAR202_clade	s__uncultured_bacterium
e4fdf0e7ebfaea088db23e6d94347ede	67	0.811	d__Bacteria	p__Proteobacteria	c__Gammaproteobacteria	o__Pseudomonadales	f__OM182_clade	g__OM182_clade	s__uncultured_gamma
b4f24b46ee91dd94772ff65a81c35968	13	0.812	d__Bacteria	p__Acidobacteriota	c__Acidobacteriae	o__PAUC26f	f__PAUC26f	g__PAUC26f	s__uncultured_bacterium
c0e06c13527a7df33b251bef74eb6413	27	0.816	d__Bacteria	p__Proteobacteria	c__Gammaproteobacteria	o__Pseudomonadales	f__Endozoicomonadaceae	g__Endozoicomonas	s__uncultured_Spongiobacter
2d6931c11e5d8a6e511b120b16b546cf	37	0.817	d__Bacteria	p__Actinobacteriota	c__Acidimicrobiia	o__Microtrichales	f__Microtrichaceae	g__Sva0996_marine_group	s__uncultured_actinobacterium
063cce8658f9e598aa3a032f35ab3260	35	0.821	d__Bacteria	p__Chloroflexi	c__JG30-KF-CM66	o__JG30-KF-CM66	f__JG30-KF-CM66	g__JG30-KF-CM66	s__uncultured_bacterium
821d422ed366d6be46e0dd7e128db306	37	0.822	d__Bacteria	p__Actinobacteriota	c__Acidimicrobiia	o__Microtrichales	f__Microtrichaceae	g__Sva0996_marine_group	s__uncultured_bacterium
52a424fce4aff4d6ec28362bcf661481	4	0.822	d__Bacteria	p__Myxococcota	c__Polyangia	o__UASB-TL25	f__UASB-TL25	g__UASB-TL25	s__uncultured_organism
8fa45acf2e34400e7436b7e195f126cb	32	0.829	d__Bacteria	p__Gemmatimonadota	c__BD2-11_terrestrial_group	o__BD2-11_terrestrial_group	f__BD2-11_terrestrial_group	g__BD2-11_terrestrial_group	s__uncultured_bacterium
7ea25d0a24df090e2f4af36ab993b68b	3	0.838	d__Bacteria	p__Acidobacteriota	c__Thermoanaerobaculia	o__Thermoanaerobaculales	f__Thermoanaerobaculaceae	g__Subgroup_10	s__uncultured_bacterium
6c5d74165cd009d17fa96d1b8298b540	5	0.841	d__Bacteria	p__Bacteroidota	c__Rhodothermia	o__Rhodothermales	f__Rhodothermaceae	g__uncultured	s__uncultured_bacterium

0a1eea63513f3603f9dea09ceb791adb	11	0.842	d__Bacteria	p__Acidobacteriota	c__Vicinamibacteria	o__Vicinamibacterales	f__uncultured	g__uncultured	
1336ac2dcb6c41da80850abbd5ff9717	5	0.843	d__Bacteria	p__Bacteroidota	c__Bacteroidia	o__Cytophagales	f__Cyclobacteriaceae	g__uncultured	s__uncultured_bacterium
d1b3ed6e18de40d28dcdcd4e739cfe4d	28	0.846	d__Bacteria	p__Actinobacteriota	c__Acidimicrobiia	o__Microtrichales	f__Microtrichaceae	g__Sva0996_marine_group	s__uncultured_actinobacterium
921dd46bd2764944eaccbbabaef7e319	39	0.847	d__Bacteria	p__Chloroflexi	c__Anaerolineae	o__SBR1031	f__A4b	g__A4b	s__uncultured_Chloroflexus
17697f9c114787c0080be460cdd42334	19	0.850	d__Bacteria	p__Acidobacteriota	c__Vicinamibacteria	o__Subgroup_9	f__Subgroup_9	g__Subgroup_9	
1d1041b03ece5361273fa8d621589bd8	14	0.850	d__Bacteria	p__Actinobacteriota	c__Acidimicrobiia	o__Microtrichales	f__Microtrichaceae	g__Sva0996_marine_group	s__uncultured_bacterium
fc34b966d1171ec2f8b4d22d800d3cf5	3	0.853	d__Bacteria	p__Proteobacteria	c__Alphaproteobacteria	o__Defluviicoccales	f__uncultured	g__uncultured	s__uncultured_bacterium
59d5d1cd97997b4892fccce17f150487	11	0.856	d__Bacteria	p__Planctomycetota	c__Planctomycetes	o__Pirellulales	f__Pirellulaceae	g__Blastopirellula	s__uncultured_bacterium
ae40de0c28ef2b2cdfe5c34e4eec3527	11	0.857	d__Bacteria	p__Chloroflexi	c__Dehalococcoidia	o__SAR202_clade	f__SAR202_clade	g__SAR202_clade	s__uncultured_Chloroflexi
9993e12043c4fb45d807c6c1efbfb5f2	6	0.858	d__Bacteria	p__Chloroflexi	c__Anaerolineae	o__SBR1031	f__A4b	g__A4b	s__uncultured_Chloroflexus
b504005fe3e7a782ec414f224b8ce9b6	48	0.861	d__Bacteria	p__Proteobacteria	c__Gammaproteobacteria	o__Pseudomonadales	f__KI89A_clade	g__KI89A_clade	s__uncultured_organism
a4a2b3ac9ec1333d2c8b5c90e9717ac6	103	0.864	d__Bacteria	p__Chloroflexi	c__Dehalococcoidia	o__SAR202_clade	f__SAR202_clade	g__SAR202_clade	s__uncultured_bacterium
effa34f80a6e5c8e6504ed2cf928ac3b	22	0.866	d__Bacteria	p__Actinobacteriota	c__Acidimicrobiia	o__Actinomarinales	f__uncultured	g__uncultured	s__uncultured_actinobacterium
0841456658ceb43c544abe94cc4ae7e3	4	0.867	d__Bacteria	p__Planctomycetota	c__Phycisphaerae	o__Phycisphaerales	f__Phycisphaeraceae	g__Urania-1B-19_marine_	s__uncultured_bacterium

								sediment_group	
e33f8f3738752389c80d6c3d34c74b15	3	0.867	d__Bacteria	p__Myxococcota	c__bacteriap25	o__bacteriap25	f__bacteriap25	g__bacteriap25	s__uncultured_bacterium
dc1a9947c5acc6a1376666fee2e16fdb	24	0.868	d__Bacteria	p__Chloroflexi	c__Dehalococcoidia	o__SAR202_clade	f__SAR202_clade	g__SAR202_clade	s__uncultured_bacterium
4fd5576a5baf4b98a6a318550e57e69c	4	0.869	d__Bacteria	p__NB1-j	c__NB1-j	o__NB1-j	f__NB1-j	g__NB1-j	s__marine_metagenome
5290a8adb37df9cda667a5191872b660	62	0.872	d__Bacteria	p__Proteobacteria	c__Alphaproteobacteria	o__Defluviicoccales	f__uncultured	g__uncultured	s__uncultured_bacterium
9bbbe63f9b1101b0a25246dc85ed52cb	6	0.873	d__Bacteria	p__Cyanobacteria	c__Cyanobacteriia	o__Synechococcales	f__Cyanobiaceae	g__Synechococcus_CC9902	s__uncultured_bacterium
a2bd0c7a17499882cf2b4002d4c302e6	19	0.874	d__Bacteria	p__Chloroflexi	c__JG30-KF-CM66	o__JG30-KF-CM66	f__JG30-KF-CM66	g__JG30-KF-CM66	s__uncultured_Chloroflexi
85d23045246eaa2920d7c94ddcf12aa8	3	0.875	d__Bacteria	p__Bacteroidota	c__Bacteroidia	o__Flavobacteriales	f__Flavobacteriaceae	g__uncultured	
ac1fb43026ebd49c88379cb0c0c2f61d	2	0.877	d__Bacteria	p__Myxococcota	c__bacteriap25	o__bacteriap25	f__bacteriap25	g__bacteriap25	s__uncultured_bacterium
2e542a0ba846e7627d36bece76b9a0e7	28	0.878	d__Bacteria	p__Chloroflexi	c__Dehalococcoidia	o__SAR202_clade	f__SAR202_clade	g__SAR202_clade	s__uncultured_bacterium
aca207208370b9dbea3fe05cfb879c4f	3	0.879	d__Bacteria	p__Acidobacteriota	c__Thermoanaerobaculia	o__Thermoanaerobaculales	f__Thermoanaerobaculaceae	g__Subgroup_10	s__uncultured_bacterium
6ef920ef7d377980129e77026a0b58db	10	0.881	d__Bacteria	p__Gemmatimonadota	c__BD2-11_terrestrial_group	o__BD2-11_terrestrial_group	f__BD2-11_terrestrial_group	g__BD2-11_terrestrial_group	s__uncultured_bacterium
7f899072063570156c7f99ab326194ed	2	0.885	d__Bacteria	p__Chloroflexi	c__Dehalococcoidia	o__SAR202_clade	f__SAR202_clade	g__SAR202_clade	s__uncultured_SAR202
341e04c55cbdcb398cae9ec0e7f37629	55	0.887	d__Bacteria	p__Poribacteria	c__Poribacteria	o__Poribacteria	f__Poribacteria	g__Poribacteria	s__uncultured_Planctomycetales

f7bf4b1e175256ac49b4379afba5b8a5	21	0.887	d__Bacteria	p__Gemmatimonadota	c__BD2-11_terrestrial_group	o__BD2-11_terrestrial_group	f__BD2-11_terrestrial_group	g__BD2-11_terrestrial_group	s__uncultured_bacterium
97fb8ec1addf7f02c1b8ff7f49627563	114	0.888	d__Bacteria	p__Proteobacteria	c__Gammaproteobacteria	o__HOC36	f__HOC36	g__HOC36	s__uncultured_gamma
04b360a26fed057128b32ccb4ebf9c0c	15	0.890	d__Bacteria	p__Proteobacteria	c__Alphaproteobacteria	o__Defluviicoccales	f__uncultured	g__uncultured	s__uncultured_bacterium
88b6d081caec18a35df2037295886130	15	0.891	d__Bacteria	p__Chloroflexi	c__Anaerolineae	o__SBR1031	f__A4b	g__A4b	s__uncultured_Chloroflexus
d0ac5be8625109ccddadbc20b7c0be0a	12	0.896	d__Bacteria	p__Chloroflexi	c__JG30-KF-CM66	o__JG30-KF-CM66	f__JG30-KF-CM66	g__JG30-KF-CM66	s__uncultured_Chloroflexi
1a10b4044c1454254394f8b7584a9bbe	7	0.898	d__Bacteria	p__Verrucomicrobiota	c__Verrucomicrobiae	o__Verrucomicrobiales	f__DEV007	g__DEV007	s__uncultured_bacterium
18c45cbfc0b04eff5105f43dda497324	10	0.899	d__Bacteria	p__Proteobacteria	c__Gammaproteobacteria	o__pItb-vmat-80	f__pItb-vmat-80	g__pItb-vmat-80	s__uncultured_bacterium
05570da352ff8bd242da1548e1db25fc	177	0.900	d__Bacteria	p__Chloroflexi	c__Dehalococcoidia	o__SAR202_clade	f__SAR202_clade	g__SAR202_clade	s__uncultured_bacterium
692d2d90f53fba93c1c6d559bd457db8	7	0.903	d__Bacteria	p__Patescibacteria	c__Parcubacteria	o__Candidatus_Kaiserbacteria	f__Candidatus_Kaiserbacteria	g__Candidatus_Kaiserbacteria	s__uncultured_bacterium
cf8c413052193af977287d9da18c4d39	94	0.909	d__Bacteria	p__Chloroflexi	c__Anaerolineae	o__SBR1031	f__A4b	g__A4b	s__uncultured_Chloroflexus
cb18e74941b79d6db132e732f427f189	5	0.910	d__Bacteria	p__Verrucomicrobiota	c__Verrucomicrobiae	o__Verrucomicrobiales	f__Rubritaleaceae	g__Roseibacillus	s__uncultured_bacterium
d2bb7b1db3ad4a010e025afd7eb403b6	13	0.911	d__Bacteria	p__Planctomycetota	c__Planctomycetes	o__Pirellulales	f__Pirellulaceae	g__Blastopirellula	s__uncultured_bacterium
42018da12a403e11b25a7b0c9cf0074a	24	0.915	d__Bacteria	p__Proteobacteria	c__Alphaproteobacteria	o__Defluviicoccales	f__uncultured	g__uncultured	s__uncultured_bacterium
a850be3eb1a02351c6f4eb993ff2279b	12	0.918	d__Bacteria	p__Chloroflexi	c__JG30-KF-CM66	o__JG30-KF-CM66	f__JG30-KF-CM66	g__JG30-KF-CM66	s__uncultured_Chloroflexi

ed467ac524267642a0763f8cd1fd6ff6	2	0.919	d__Bacteria	p__Proteobacteria	c__Gammaproteobacteria	o__HOC36	f__HOC36	g__HOC36	s__uncultured_bacterium
e4c488eaf2b97d8fe96c475aa1bb0e16	36	0.922	d__Bacteria	p__Gemmatimonadota	c__BD2-11_terrestrial_group	o__BD2-11_terrestrial_group	f__BD2-11_terrestrial_group	g__BD2-11_terrestrial_group	s__uncultured_delta
7002c643193e83563c91d1e6ffc44302	222	0.928	d__Bacteria	p__Gemmatimonadota	c__BD2-11_terrestrial_group	o__BD2-11_terrestrial_group	f__BD2-11_terrestrial_group	g__BD2-11_terrestrial_group	s__uncultured_bacterium
a7680451ecd5a8cc5c1a80cfeee4e3c1	2	0.929	d__Bacteria	p__Bacteroidota	c__Rhodothermia	o__Rhodothermales	f__Rhodothermaceae	g__uncultured	
aa3fa7c5956aa32d1db2df30c9a5c1a8	8	0.930	d__Bacteria	p__Bacteroidota	c__Rhodothermia	o__Rhodothermales	f__Rhodothermaceae	g__uncultured	s__uncultured_bacterium
9f5e333b294bfe0c909f8d539b34522b	22	0.930	d__Bacteria	p__Chloroflexi	c__JG30-KF-CM66	o__JG30-KF-CM66	f__JG30-KF-CM66	g__JG30-KF-CM66	s__uncultured_Chloroflexi
d737fcb1d338aadb5780a96821ac30b5	19	0.933	d__Bacteria	p__Proteobacteria	c__Alphaproteobacteria	o__Defluviicoccales	f__uncultured	g__uncultured	s__uncultured_bacterium
e4fc3333123c849329d7675c66df815a	43	0.934	d__Bacteria	p__Proteobacteria	c__Alphaproteobacteria	o__Defluviicoccales	f__uncultured	g__uncultured	s__uncultured_bacterium
3b071bdfd3abadc24a92b02d2c2f3e5d	18	0.934	d__Bacteria	p__Chloroflexi	c__Dehalococcoidia	o__SAR202_clade	f__SAR202_clade	g__SAR202_clade	s__uncultured_bacterium
d20f87727008ff492ba0edea42e1591d	23	0.936	d__Bacteria	p__Chloroflexi	c__Dehalococcoidia	o__SAR202_clade	f__SAR202_clade	g__SAR202_clade	s__uncultured_SAR202
11fb7eeed4c7ca30e5792a6b9e9f08fc	5	0.937	d__Bacteria	p__Chloroflexi	c__Dehalococcoidia	o__SAR202_clade	f__SAR202_clade	g__SAR202_clade	s__uncultured_bacterium
a97eae53c423c75d5bf421e68fd34ccd	144	0.938	d__Bacteria	p__Chloroflexi	c__Dehalococcoidia	o__SAR202_clade	f__SAR202_clade	g__SAR202_clade	s__uncultured_SAR202
08ceb49ab74ff5b81fd000a3f51823a0	5	0.940	d__Bacteria	p__Chloroflexi	c__Dehalococcoidia	o__SAR202_clade	f__SAR202_clade	g__SAR202_clade	s__uncultured_bacterium
9facd2806bfdaa6cbe1507595bf0024a	1	0.941	d__Bacteria	p__Planctomycetota	c__Planctomycetes	o__Pirellulales	f__Pirellulaceae	g__Blastopirellula	s__uncultured_bacterium

b886eca1b64d21bd4e06c024ad5ede9b	2	0.948	d__Bacteria	p__Acidobacteriota	c__Thermoanaerobaculia	o__Thermoanaerobaculales	f__Thermoanaerobaculaceae	g__Subgroup_10	s__uncultured_bacterium
557768fafcd2bbf326b75ad9efc67ed6	16	0.950	d__Bacteria	p__Acidobacteriota	c__Vicinamibacteria	o__Vicinamibacterales	f__uncultured	g__uncultured	s__uncultured_bacterium
49a784104a72e1af36f7f4908333109a	57	0.951	d__Bacteria	p__Actinobacteriota	c__Acidimicrobiia	o__Microtrichales	f__Microtrichaceae	g__Sva0996_marine_group	s__uncultured_bacterium
bde571c1071072dab43ee92c07079ca7	11	0.955	d__Bacteria	p__Gemmatimonadota	c__BD2-11_terrestrial_group	o__BD2-11_terrestrial_group	f__BD2-11_terrestrial_group	g__BD2-11_terrestrial_group	s__uncultured_bacterium
4c31d6515474234ca32b8c4ba923f87a	38	0.957	d__Bacteria	p__Acidobacteriota	c__Vicinamibacteria	o__Vicinamibacterales	f__uncultured	g__uncultured	s__uncultured_bacterium
fc999a4b895b891b646b081d51b595e0	33	0.958	d__Bacteria	p__Entotheonellaeota	c__Entotheonellia	o__Entotheonellales	f__Entotheonellaceae	g__Entotheonellaceae	s__uncultured_delta
f41e4481e87685e25bae1ee5bacf601c	2	0.958	d__Bacteria	p__SAR324_clade(Marine_group_B)	c__SAR324_clade(Marine_group_B)	o__SAR324_clade(Marine_group_B)	f__SAR324_clade(Marine_group_B)	g__SAR324_clade(Marine_group_B)	s__uncultured_bacterium
f9404661ca724985ec4de10ad921336f	63	0.959	d__Bacteria	p__Proteobacteria	c__Gammaproteobacteria	o__EPR3968-O8a-Bc78	f__EPR3968-O8a-Bc78	g__EPR3968-O8a-Bc78	s__uncultured_bacterium
cd7a605b7c7129c35c2b41a2c84441bb	139	0.962	d__Bacteria	p__Chloroflexi	c__Dehalococcoidia	o__S085	f__S085	g__S085	s__uncultured_bacterium
3ef4191284c4d7878a22a186119b974f	32	0.964	d__Bacteria	p__Chloroflexi	c__Dehalococcoidia	o__SAR202_clade	f__SAR202_clade	g__SAR202_clade	s__uncultured_SAR202
33be78ae997e790758ecac863b3e0e5f	3	0.964	d__Bacteria	p__Proteobacteria	c__Gammaproteobacteria	o__Nitrosococcales	f__Nitrosococcaceae	g__AqS1	s__uncultured_bacterium
7bc1a7ab05aaa1be8999f4f56fc0d5e9	91	0.965	d__Bacteria	p__Entotheonellaeota	c__Entotheonellia	o__Entotheonellales	f__Entotheonellaceae	g__Entotheonellaceae	s__uncultured_delta
8e7bdd816e733eed974bacce33d6f888	4	0.968	d__Bacteria	p__Chloroflexi	c__Anaerolineae	o__Caldilineales	f__Caldilineaceae	g__uncultured	s__uncultured_bacterium

0728934ec46cd599f6535e8634bea95b	18	0.968	d__Bacteria	p__Proteobacteria	c__Alphaproteobacteria	o__Rhodobacterales	f__Rhodobacteraceae	g__uncultured	
345da844448e3ae5d0ad0606a247fb71	2	0.968	d__Bacteria	p__Chloroflexi	c__JG30-KF-CM66	o__JG30-KF-CM66	f__JG30-KF-CM66	g__JG30-KF-CM66	s__uncultured_bacterium
99b248028a11c8d01a7abb844f4338be	2	0.968	d__Bacteria	p__Actinobacteriota	c__Acidimicrobiia	o__Actinomarinales	f__uncultured	g__uncultured	
2c1cfcd31b173b430b31d311e9e66c72	80	0.969	d__Bacteria	p__Entotheonellaeota	c__Entotheonellia	o__Entotheonellales	f__Entotheonellaceae	g__Entotheonellaceae	s__uncultured_delta
afc4bc6ff20a7f81d11d4ab99b8b6bf9	25	0.969	d__Bacteria	p__Gemmatimonadota	c__BD2-11_terrestrial_group	o__BD2-11_terrestrial_group	f__BD2-11_terrestrial_group	g__BD2-11_terrestrial_group	s__uncultured_bacterium
e12634649136c9b7c1e826a6811b0590	2	0.969	d__Bacteria	p__Planctomycetota	c__OM190	o__OM190	f__OM190	g__OM190	s__uncultured_bacterium
c2d6fdc5eb1836a9dda7a8cb75085efc	3	0.970	d__Bacteria	p__Gemmatimonadota	c__BD2-11_terrestrial_group	o__BD2-11_terrestrial_group	f__BD2-11_terrestrial_group	g__BD2-11_terrestrial_group	s__uncultured_delta
0540294cc2626f66cccd595861a5bed7	92	0.970	d__Bacteria	p__Actinobacteriota	c__Acidimicrobiia	o__Microtrichales	f__Microtrichaceae	g__Sva0996_marine_group	s__uncultured_bacterium
44f9206d69a28e8d3d02b7bd32df7829	24	0.971	d__Archaea	p__Crenarchaeota	c__Nitrososphaeria	o__Nitrosopumilales	f__Nitrosopumilaceae	g__Candidatus_Nitrosopumilus	s__uncultured_archaeon
f3b8314c5a282f5c1ef6e876053b4bdc	21	0.971	d__Bacteria	p__Acidobacteriota	c__Vicinamibacteria	o__Vicinamibacterales	f__uncultured	g__uncultured	s__uncultured_bacterium
113197124816e7124d0a939782d2fd82	19	0.971	d__Bacteria	p__Chloroflexi	c__Dehalococcoidia	o__SAR202_clade	f__SAR202_clade	g__SAR202_clade	s__uncultured_bacterium
40c2b630e6829314f805e1d934951f02	38	0.972	d__Bacteria	p__Bacteroidota	c__Rhodothermia	o__Rhodothermales	f__Rhodothermaceae	g__uncultured	s__uncultured_bacterium
ab8ecc7d8382d653756f215910174e58	52	0.972	d__Bacteria	p__Chloroflexi	c__Dehalococcoidia	o__SAR202_clade	f__SAR202_clade	g__SAR202_clade	s__uncultured_bacterium
bd0d0e0f25cea14bc026f096128a2ba7	3	0.972	d__Bacteria	p__Chloroflexi	c__Dehalococcoidia	o__SAR202_clade	f__SAR202_clade	g__SAR202_clade	

84f72901395642000ee1f5eb76b76157	17	0.972	d__Bacteria	p__Acidobacteriota	c__Vicinamibacteria	o__Vicinamibacterales	f__uncultured	g__uncultured	s__uncultured_bacterium
4948624a3b144b43755815d45421a21d	12	0.975	d__Bacteria	p__Actinobacteriota	c__Thermoleophilia	o__Gaiellales	f__uncultured	g__uncultured	s__uncultured_bacterium
121e54c4363c2e68615d3719ace1eb15	47	0.975	d__Bacteria	p__Proteobacteria	c__Gammaproteobacteria	o__Pseudomonadales	f__Pseudohongiellaceae	g__Pseudohongiella	s__uncultured_bacterium
1fca9214ea64502cf023fa6372a8498d	59	0.976	d__Bacteria	p__Proteobacteria	c__Alphaproteobacteria	o__Kiloniellales	f__Kiloniellaceae	g__uncultured	s__uncultured_bacterium
bf7c48db5b7f62245f4e6507542ae90d	48	0.976	d__Bacteria	p__Actinobacteriota	c__Acidimicrobiia	o__Microtrichales	f__Microtrichaceae	g__Sva0996_marine_group	s__uncultured_bacterium
bc83829b014ac67af983e6922060089b	104	0.976	d__Bacteria	p__Proteobacteria	c__Gammaproteobacteria	o__Pseudomonadales	f__KI89A_clade	g__KI89A_clade	s__uncultured_bacterium
e7adbfc5294f0c32a36807cb97d3fdf1	5	0.976	d__Bacteria	p__Actinobacteriota	c__Thermoleophilia	o__Gaiellales	f__uncultured	g__uncultured	s__uncultured_bacterium
ca2cb420736f30ff496de4e213a198b0	6	0.976	d__Bacteria	p__Chloroflexi	c__Anaerolineae	o__Caldilineales	f__Caldilineaceae	g__uncultured	s__uncultured_bacterium
2b0e2239e896ae2441c98e199feae7c1	1	0.977	d__Bacteria	p__Chloroflexi	c__Dehalococcoidia	o__SAR202_clade	f__SAR202_clade	g__SAR202_clade	
d4ae7081f0fb816b16a68049b1f5381d	13	0.978	d__Bacteria	p__Proteobacteria	c__Gammaproteobacteria	o__UBA10353_marine_group	f__UBA10353_marine_group	g__UBA10353_marine_group	s__uncultured_bacterium
092bc671bc1d2c9f2318e9f5f22a2ea1	16	0.979	d__Bacteria	p__Myxococcota	c__bacteriap25	o__bacteriap25	f__bacteriap25	g__bacteriap25	s__uncultured_delta
318c3c6a14aed9d629284ae6c933bc18	792	0.980	d__Bacteria	p__Actinobacteriota	c__Acidimicrobiia	o__Microtrichales	f__Microtrichaceae	g__Sva0996_marine_group	s__uncultured_bacterium
e68d370d47f1c36c771646ca1ad728ff	136	0.980	d__Bacteria	p__Actinobacteriota	c__Acidimicrobiia	o__Microtrichales	f__Microtrichaceae	g__Sva0996_marine_group	s__uncultured_actinobacterium
6e767ed893f8f03b30ea9078727bbd01	13	0.981	d__Bacteria	p__Proteobacteria	c__Gammaproteobacteria	o__HOC36	f__HOC36	g__HOC36	s__uncultured_gamma

79ea44fbe6094230dcbb0e9f43858b6b	15	0.981	d__Bacteria	p__Chloroflexi	c__Dehalococcoidia	o__SAR202_clade	f__SAR202_clade	g__SAR202_clade	s__uncultured_Chloroflexus
2de5b8a916bdc5434ed59590f62e4c98	4	0.981	d__Bacteria	p__Acidobacteriota	c__Vicinamibacteria	o__Vicinamibacterales	f__uncultured	g__uncultured	s__uncultured_bacterium
9be79729aed669c05a09e04ded902c0d	3	0.982	d__Bacteria	p__SAR324_clade(Marine_group_B)	c__SAR324_clade(Marine_group_B)	o__SAR324_clade(Marine_group_B)	f__SAR324_clade(Marine_group_B)	g__SAR324_clade(Marine_group_B)	s__uncultured_bacterium
77d8aa70a5e03dffc243d9bf728eb157	1	0.982	d__Bacteria	p__Proteobacteria	c__Gammaproteobacteria	o__EPR3968-O8a-Bc78	f__EPR3968-O8a-Bc78	g__EPR3968-O8a-Bc78	s__uncultured_bacterium
51ea2cda562169e7b6ddc12ccf957ef3	225	0.984	d__Bacteria	p__Chloroflexi	c__Dehalococcoidia	o__SAR202_clade	f__SAR202_clade	g__SAR202_clade	s__uncultured_SAR202
14e0a510c77d1c3f524a8e3ede9e15a2	2	0.984	d__Bacteria	p__Chloroflexi	c__Anaerolineae	o__Caldilineales	f__Caldilineaceae	g__uncultured	s__uncultured_Chloroflexus
52084569dfce217b56f280f56c046d43	7	0.984	d__Bacteria	p__Dadabacteria	c__Dadabacteriia	o__Dadabacteriales	f__Dadabacteriales	g__Dadabacteriales	s__uncultured_delta
a452e7d2687fe39c7fad9a736ee2aaeb	15	0.985	d__Bacteria	p__Actinobacteriota	c__Acidimicrobiia	o__Microtrichales	f__Microtrichaceae	g__Sva0996_marine_group	s__uncultured_bacterium
187bdf5cebe94820fee030f095b6e5a4	9	0.985	d__Bacteria	p__Chloroflexi	c__Anaerolineae	o__SBR1031	f__A4b	g__A4b	s__uncultured_Chloroflexi
5f0ae4a58138c6931ad2dc2859dc9617	35	0.985	d__Bacteria	p__Entotheonellaeota	c__Entotheonellia	o__Entotheonellales	f__Entotheonellaceae	g__Entotheonellaceae	s__uncultured_delta
49b2b7c34953ec000a91c59f544587d8	13	0.985	d__Bacteria	p__Actinobacteriota	c__Acidimicrobiia	o__Microtrichales	f__Microtrichaceae	g__uncultured	s__uncultured_bacterium
6ce359edd2145bb37b0fe5100c5682b3	174	0.986	d__Bacteria	p__Proteobacteria	c__Gammaproteobacteria	o__EPR3968-O8a-Bc78	f__EPR3968-O8a-Bc78	g__EPR3968-O8a-Bc78	s__uncultured_bacterium
7636100333049f69c6f1f351ccc1cb41	2	0.986	d__Bacteria	p__Planctomycetota	c__Planctomycetes	o__Planctomycetales	f__uncultured	g__uncultured	s__uncultured_bacterium

51ccc32e03c40a99153df9c03036e848	16	0.986	d__Bacteria	p__Gemmatimonadota	c__BD2-11_terrestrial_group	o__BD2-11_terrestrial_group	f__BD2-11_terrestrial_group	g__BD2-11_terrestrial_group	s__uncultured_delta
6f190ae19e29de9d3ac026f29743e42d	22	0.987	d__Bacteria	p__Proteobacteria	c__Alphaproteobacteria	o__Rhodobacterales	f__Rhodobacteraceae	g__Albidovulum	s__uncultured_Alphaproteobacteria
ef2f6ba168e155cabcef8c6388aff9d3	64	0.987	d__Bacteria	p__Entotheonellaeota	c__Entotheonellia	o__Entotheonellales	f__Entotheonellaceae	g__Entotheonellaceae	s__uncultured_delta
2a1d60675b24dd00980562b476989701	7	0.988	d__Bacteria	p__Chloroflexi	c__Dehalococcoidia	o__SAR202_clade	f__SAR202_clade	g__SAR202_clade	s__uncultured_bacterium
938eac81de414120530b016bbac17c2e	56	0.988	d__Bacteria	p__Entotheonellaeota	c__Entotheonellia	o__Entotheonellales	f__Entotheonellaceae	g__Entotheonellaceae	s__uncultured_delta
f25143a522db6e2446e4fae9dae92627	24	0.988	d__Bacteria	p__Acidobacteriota	c__Vicinamibacteria	o__Subgroup_9	f__Subgroup_9	g__Subgroup_9	s__uncultured_bacterium
93853cf2507be4d84e400787fa0c6c31	45	0.989	d__Bacteria	p__Entotheonellaeota	c__Entotheonellia	o__Entotheonellales	f__Entotheonellaceae	g__Entotheonellaceae	s__uncultured_delta
f0672c674b0f9c2731ce75190e47b864	5	0.989	d__Bacteria	p__Chloroflexi	c__Dehalococcoidia	o__SAR202_clade	f__SAR202_clade	g__SAR202_clade	s__uncultured_Chloroflexi
a5f252c77ba9ec05e3210792c7ad8a69	238	0.989	d__Bacteria	p__Proteobacteria	c__Gammaproteobacteria	o__EPR3968-O8a-Bc78	f__EPR3968-O8a-Bc78	g__EPR3968-O8a-Bc78	s__uncultured_bacterium
37cf56ef83ac5322df561dee057b932b	2	0.990	d__Bacteria	p__Actinobacteriota	c__Acidimicrobiia	o__Microtrichales	f__Microtrichaceae	g__uncultured	s__Callinectes_sapidus
4b0d254981111187cb3bff3a39d78b9a	3	0.990	d__Bacteria	p__Bacteroidota	c__Bacteroidia	o__Chitinophagales	f__uncultured	g__uncultured	s__uncultured_Bacteroidetes
546627ba3f2471e8f05ccfdb26c29d68	11	0.990	d__Bacteria	p__AncK6	c__AncK6	o__AncK6	f__AncK6	g__AncK6	s__uncultured_bacterium
34d6ace593c6ed2c515ceaf6be8dcf02	12	0.990	d__Bacteria	p__Poribacteria	c__Poribacteria	o__Poribacteria	f__Poribacteria	g__Poribacteria	s__Candidatus_Poribacteria
718cf739a6f41e63ee49757473427e64	22	0.991	d__Bacteria	p__Proteobacteria	c__Alphaproteobacteria	o__uncultured	f__uncultured	g__uncultured	s__uncultured_Alphaproteobacteria

5ebb0b540c6a0e4b70dc11207fddf8e2	14	0.992	d__Bacteria	p__Bacteroidota	c__Rhodothermia	o__Rhodothermales	f__Rhodothermaceae	g__uncultured	s__uncultured_Rhodothermaceae
6641fd66d5fc27db144e7a93786136d4	26	0.992	d__Bacteria	p__Proteobacteria	c__Alphaproteobacteria	o__Puniceispirillales	f__EF100-94H03	g__EF100-94H03	s__uncultured_bacterium
106c6b343425c2c883720f63cd5c8a1a	6	0.992	d__Bacteria	p__Acidobacteriota	c__Thermoanaerobaculia	o__Thermoanaerobaculales	f__Thermoanaerobaculaceae	g__Subgroup_10	s__uncultured_bacterium
f8001f9bc1459e220983121817265230	178	0.993	d__Bacteria	p__Chloroflexi	c__Anaerolineae	o__Caldilineales	f__Caldilineaceae	g__uncultured	s__uncultured_Caldilinea
62b488835fc9b79e9788edc4e71b5330	6	0.993	d__Bacteria	p__Poribacteria	c__Poribacteria	o__Poribacteria	f__Poribacteria	g__Poribacteria	s__Candidatus_Poribacteria
0d864eef2bc2c0e980088ad43d873c36	73	0.994	d__Bacteria	p__Actinobacteriota	c__Acidimicrobiia	o__Microtrichales	f__Microtrichaceae	g__Sva0996_marine_group	s__uncultured_actinobacterium
c383a114832dfa8795e810cd8a4fca81	2	0.994	d__Bacteria	p__Actinobacteriota	c__Acidimicrobiia	o__Microtrichales	f__Microtrichaceae	g__Sva0996_marine_group	
b6b7b9d8cf98add86b902b9d6b80840e	4	0.994	d__Bacteria	p__Acidobacteriota	c__Thermoanaerobaculia	o__Thermoanaerobaculales	f__Thermoanaerobaculaceae	g__Subgroup_10	s__uncultured_bacterium
0bce9e193dd20323ff34751f8711d95e	60	0.994	d__Bacteria	p__Poribacteria	c__Poribacteria	o__Poribacteria	f__Poribacteria	g__Poribacteria	s__uncultured_Poribacteria
1d59c38dea98282ee2916d2c7ca59ddf	10	0.994	d__Bacteria	p__Myxococcota	c__bacteriap25	o__bacteriap25	f__bacteriap25	g__bacteriap25	s__uncultured_delta
4e046c3bf5c99fb874bf0ae8763d0232	3	0.994	d__Bacteria	p__Entotheonellaeota	c__Entotheonellia	o__Entotheonellales	f__Entotheonellaceae	g__Entotheonellaceae	s__uncultured_delta
1c8e0d79f0994039ead2a4376f7f12f5	3	0.994	d__Bacteria	p__Acidobacteriota	c__Subgroup_5	o__Subgroup_5	f__Subgroup_5	g__Subgroup_5	s__uncultured_bacterium
b11e746055b3692569dac165ecf828e9	139	0.994	d__Bacteria	p__Actinobacteriota	c__Acidimicrobiia	o__Microtrichales	f__Microtrichaceae	g__Sva0996_marine_group	s__uncultured_bacterium
31e1f83d16331db46c6b60b39638d156	17	0.994	d__Bacteria	p__Chloroflexi	c__Dehalococcoidia	o__SAR202_clade	f__SAR202_clade	g__SAR202_clade	s__uncultured_bacterium

f74bf88be3c3fc3aa089270adfe9964d	49	0.995	d__Bacteria	p__Proteobacteria	c__Gammaproteobacteria	o__JTB23	f__JTB23	g__JTB23	s__uncultured_proteobacterium
e526c192d3aed024dd9ae34b2defbaae	14	0.995	d__Bacteria	p__Acidobacteriota	c__Thermoanaerobaculia	o__Thermoanaerobaculales	f__Thermoanaerobaculaceae	g__Subgroup_10	s__uncultured_bacterium
81249f516b87579c2b52f79dbe55ba27	2	0.996	d__Bacteria	p__Bdellovibrionota	c__Bdellovibrionia	o__Bdellovibrionales	f__Bdellovibrionaceae	g__Bdellovibrio	s__uncultured_bacterium
3932d0fa1f1d94a503cc7e2650d396e7	40	0.996	d__Bacteria	p__Actinobacteriota	c__Acidimicrobiia	o__Actinomarinales	f__uncultured	g__uncultured	
65255d19bddd799c294484638f98a1ef	4	0.996	d__Bacteria	p__Chloroflexi	c__TK17	o__TK17	f__TK17	g__TK17	s__uncultured_Chloroflexi
e5ad1dabf32b619f429e1785386c5a2a	14	0.996	d__Bacteria	p__Chloroflexi	c__Anaerolineae	o__SBR1031	f__A4b	g__A4b	s__uncultured_Chloroflexus
6cb98e126b917bd7fa82cfac38a6a1be	2	0.996	d__Bacteria	p__Proteobacteria	c__Gammaproteobacteria	o__JTB23	f__JTB23	g__JTB23	s__uncultured_proteobacterium
0492e7d534276b00f14cf30b1e9b3bc4	49	0.996	d__Bacteria	p__Proteobacteria	c__Gammaproteobacteria	o__JTB23	f__JTB23	g__JTB23	
d27c166c4cc4da452671ae88173e7be3	5	0.996	d__Bacteria	p__Proteobacteria	c__Alphaproteobacteria	o__Caulobacterales	f__Parvularculaceae	g__Marinicaulis	s__uncultured_bacterium
789a12a0673bd1b350e257d08787d1f1	4	0.997	d__Bacteria	p__Bacteroidota	c__Bacteroidia	o__Flavobacteriales	f__Flavobacteriaceae	g__Aquibacter	s__uncultured_bacterium
6c2c6037416c92b0d86014b0d3d4b525	2	0.997	d__Bacteria	p__Verrucomicrobiota	c__Verrucomicrobiae	o__Opitutales	f__Puniceicoccaceae	g__Cerasicoccus	s__uncultured_Verrucomicrobia
5a8faed815dd2447ad9a4c2984a0b083	14	0.997	d__Bacteria	p__Chloroflexi	c__Anaerolineae	o__Caldilineales	f__Caldilineaceae	g__uncultured	s__uncultured_Chloroflexus
db59ced496eb6efa933adeabb457059e	59	0.997	d__Bacteria	p__Actinobacteriota	c__Acidimicrobiia	o__Microtrichales	f__Microtrichaceae	g__uncultured	s__uncultured_bacterium
f0f0515a647e20f3a63fa737a92c173f	22	0.997	d__Bacteria	p__Poribacteria	c__Poribacteria	o__Poribacteria	f__Poribacteria	g__Poribacteria	s__Candidatus_Poribacteria

f8c2578000e8d8783bdf16642fb4c705	21	0.997	d__Bacteria	p__Acidobacteriota	c__Subgroup_21	o__Subgroup_21	f__Subgroup_21	g__Subgroup_21	s__uncultured_bacterium
a9175c7f911cb499a3df1f8b39125204	131	0.998	d__Bacteria	p__Acidobacteriota	c__Vicinamibacteria	o__Subgroup_9	f__Subgroup_9	g__Subgroup_9	s__uncultured_bacterium
f848267b7ee35a8168c5aca4c051dfc6	24	0.998	d__Bacteria	p__Chloroflexi	c__TK17	o__TK17	f__TK17	g__TK17	s__uncultured_Chloroflexi
087910d4f98c7cb5874298c6adb2b462	27	0.998	d__Bacteria	p__Proteobacteria	c__Gammaproteobacteria	o__JTB23	f__JTB23	g__JTB23	s__uncultured_proteobacterium
507f417118ba0296202f1b9bc9f35140	2	0.998	d__Bacteria	p__Chloroflexi	c__Anaerolineae	o__Caldilineales	f__Caldilineaceae	g__uncultured	s__uncultured_Chloroflexus
d33778ee477f301a6561189f608db5f1	4	0.998	d__Bacteria	p__Planctomycetota	c__Planctomycetes	o__Pirellulales	f__Pirellulaceae	g__Blastopirellula	
6c809937e62b7b9e3d9680dde8514458	8	0.998	d__Bacteria	p__Proteobacteria	c__Gammaproteobacteria	o__pItb-vmat-80	f__pItb-vmat-80	g__pItb-vmat-80	s__uncultured_bacterium
3ec1e43e77fd669fccaa8c70ac9fdb22	28	0.998	d__Bacteria	p__Proteobacteria	c__Gammaproteobacteria	o__pItb-vmat-80	f__pItb-vmat-80	g__pItb-vmat-80	s__uncultured_bacterium
af10a20a19fb941c5021ee854d0b68e3	95	0.998	d__Bacteria	p__Proteobacteria	c__Gammaproteobacteria	o__JTB23	f__JTB23	g__JTB23	
39ad9d5e1d861b8925bffb1082e586f5	241	0.998	d__Bacteria	p__Acidobacteriota	c__Vicinamibacteria	o__Subgroup_9	f__Subgroup_9	g__Subgroup_9	s__uncultured_bacterium
096925d781d8882a23672871e3054139	15	0.999	d__Bacteria	p__Proteobacteria	c__Alphaproteobacteria	o__uncultured	f__uncultured	g__uncultured	s__uncultured_Alphaproteobacteria
b6e03349e272734fbbb88342f7fafbd2	2	0.999	d__Bacteria	p__Chloroflexi	c__Anaerolineae	o__Caldilineales	f__Caldilineaceae	g__uncultured	s__uncultured_Chloroflexus
014e17b29a1c01cae4ff773cca164a1c	162	0.999	d__Bacteria	p__Actinobacteriota	c__Acidimicrobiia	o__Microtrichales	f__Microtrichaceae	g__Sva0996_marine_group	
5905024c401df868e1fd8523c4308168	3	0.999	d__Bacteria	p__Proteobacteria	c__Gammaproteobacteria	o__Burkholderiales	f__Nitrosomonadaceae		

197113967fc0 6328d4ab3b5 48355fc05	76	0.999	d__Bact eria	p__Chlorofl exi	c__Dehalococco idia	o__SAR202 _clade	f__SAR202 _clade	g__SAR202 _clade	
f72871b2770c bfacc1d095d4 5ff7b3e4	26 4	0.999	d__Bact eria	p__Actinob acteriota	c__Acidimicrob iia	o__Microtri chales	f__Microtri chaceae	g__Sva0996 _marine_gr oup	
b936c98dae87 1c62c562ca5d 09bb1809	13	0.999	d__Bact eria	p__Proteoba cteria	c__Alphaproteo bacteria	o__Rhodosp irillales	f__Magneto spiraceae	g__uncultur ed	s__uncultured _bacterium
fb2927eba4dc 540fe0243364 2782f9b6	93	0.999	d__Bact eria	p__Proteoba cteria	c__Gammaprote obacteria	o__Pseudo monadales	f__KI89A_c lade	g__KI89A_ clade	s__uncultured _bacterium
50b7c6d6c6c3 aaff46f4f1f30 4995193	11	0.999	d__Bact eria	p__Proteoba cteria	c__Gammaprote obacteria	o__Nitrosoc occales	f__Nitrosoc occaceae	g__AqS1	s__uncultured _bacterium
d77f6bd395b7 c11f9ba2aa87 20131dc9	77	0.999	d__Bact eria	p__Myxoco ccota	c__bacteriap25	o__bacteria p25	f__bacteriap 25	g__bacteria p25	s__uncultured _bacterium
046bd7b10b8 98a7f61a3155 16795093f	2	0.999	d__Bact eria	p__Cyanoba cteria	c__Vampirivibri onia	o__Caenarc aniphilales	f__Caenarca niphilales	g__Caenarc aniphilales	s__uncultured _bacterium
56f3182a7fca d82a6903ef9d 4da46423	32	0.999	d__Bact eria	p__Spirocha etota	c__Spirochaetia	o__Spirocha etales	f__Spirocha etaceae	g__Spirocha eta	s__uncultured _bacterium
98fcd625b8fd af9b647c7591 4e692861	31	0.999	d__Bact eria	p__Proteoba cteria	c__Gammaprote obacteria	o__Nitrosoc occales	f__Nitrosoc occaceae	g__AqS1	s__uncultured _bacterium
479332d756b 8cc79ab72b7e 8d816e686	2	0.999	d__Bact eria	p__Poribact eria	c__Poribacteria	o__Poribact eria	f__Poribact eria	g__Poribact eria	
c3a426b285ac 1d04ef66f01d 59891e1f	23	0.999	d__Bact eria	p__Acidoba cteriota	c__Acidobacteri ae	o__PAUC2 6f	f__PAUC26 f	g__PAUC2 6f	s__uncultured _bacterium
75caa98fc825 6bae87750f37 d9d978e4	3	0.999	d__Bact eria	p__Chlorofl exi	c__Anaerolinea e	o__Caldilin eales	f__Caldiline aceae	g__uncultur ed	s__uncultured _Chloroflexus
76116140b2a 5d2fed18cf7fe 0206f42e	1	1.000	d__Bact eria	p__Chlorofl exi	c__Dehalococco idia	o__SAR202 _clade	f__SAR202 _clade	g__SAR202 _clade	
0c33337d698 9167496e538 5e60b59e1f	3	1.000	d__Bact eria	p__Plancto mycetota	c__Planctomyce tes	o__Pirellula les	f__Pirellula ceae		

eddd9db37c6bbbd4576d42e363145c8d	23	1.000	d__Bacteria	p__Actinobacteriota	c__Acidimicrobiia	o__Microtrichales	f__Microtrichaceae	g__uncultured	s__uncultured_bacterium
aa3d9a6455de34012e8b0a8bd268b556	12	1.000	d__Bacteria	p__Proteobacteria	c__Alphaproteobacteria	o__Defluviicoccales	f__uncultured	g__uncultured	s__uncultured_bacterium
01cfcc41da2ec9782b785fcb539cb8d0	15	1.000	d__Bacteria	p__Proteobacteria	c__Alphaproteobacteria	o__Puniceispirillales	f__Puniceispirillales_Incertae_Sedis	g__Constrictibacter	s__uncultured_bacterium
a79dc64125765bd37b9cc1d984be460f	3	1.000	d__Bacteria	p__Chloroflexi	c__Anaerolineae	o__Caldilineales	f__Caldilineaceae	g__uncultured	s__uncultured_Chloroflexus
3eeb923e4f890585a6afe92b5df4facf	12	1.000	d__Bacteria	p__Chloroflexi	c__Anaerolineae	o__SBR1031	f__A4b	g__A4b	s__uncultured_Chloroflexus
5eb2f8c392bda4cc59826cef055198b4	3	1.000	d__Bacteria	p__Chloroflexi	c__Anaerolineae	o__Caldilineales	f__Caldilineaceae	g__uncultured	s__uncultured_Chloroflexus
efe90242338cd5d74ffbd877b4129b04	15	1.000	d__Bacteria	p__Proteobacteria	c__Gammaproteobacteria	o__Steroidobacterales	f__Woeseiaceae	g__JTB255_marine_benthic_group	s__uncultured_bacterium
4ba63cde9f462649c94fc3a1c3094ff8	2	1.000	d__Bacteria	p__Myxococcota	c__bacteriap25	o__bacteriap25	f__bacteriap25	g__bacteriap25	s__uncultured_bacterium
7e441884d9db8ac90f4531a71e544c6f	10	1.000	d__Bacteria	p__Proteobacteria	c__Alphaproteobacteria	o__uncultured	f__uncultured	g__uncultured	s__uncultured_Alphaproteobacteria
9a3dee015b81ba479f9297af9a452c19	109	1.000	d__Bacteria	p__Chloroflexi	c__Dehalococcoidia	o__SAR202_clade	f__SAR202_clade	g__SAR202_clade	s__uncultured_deep-sea
a7febd04d2c878e674539ac24d0c5c03	82	1.000	d__Bacteria	p__Chloroflexi	c__Dehalococcoidia	o__SAR202_clade	f__SAR202_clade	g__SAR202_clade	s__uncultured_deep-sea
9353d0ecc57543e70d84f7e37e716097	14	1.000	d__Bacteria	p__Proteobacteria	c__Alphaproteobacteria	o__Rhizobiales	f__Hyphomicrobiaceae	g__Filomicrobium	s__uncultured_bacterium
557c01a002652e4d427fd59cfed02c78	7	1.000	d__Bacteria	p__Chloroflexi	c__Anaerolineae	o__Caldilineales	f__Caldilineaceae	g__uncultured	s__uncultured_Chloroflexus
dcd2f551ef567c59a58ae6cdc8311607	3	1.000	d__Bacteria	p__Proteobacteria	c__Alphaproteobacteria	o__Rhizobiales	f__Rhizobiaceae		

d13ef7818c89584ead10559e232e2408	14	1.000	d__Bacteria	p__Proteobacteria	c__Alphaproteobacteria	o__Rhizobiales	f__Hyphomicrobiaceae	g__Filomicrobium	s__uncultured_bacterium
e9137e128559135e1a3cf6b20f59059b	24	1.000	d__Bacteria	p__Proteobacteria	c__Gammaproteobacteria	o__Pseudomonadales	f__KI89A_clade	g__KI89A_clade	s__uncultured_bacterium
388db4384d627bfcb967352e8d1eabe0	6	1.000	d__Bacteria	p__Acidobacteriota	c__Acidobacteriae	o__PAUC26f	f__PAUC26f	g__PAUC26f	s__uncultured_bacterium
ab6151de482049167acb71a2969446f5	39	1.000	d__Bacteria	p__Proteobacteria	c__Alphaproteobacteria	o__Defluviicoccales	f__uncultured	g__uncultured	s__uncultured_bacterium
17e9902770b3566c46767717386b0042	2	1.000	d__Bacteria	p__Verrucomicrobiota	c__Chlamydiae	o__Chlamydiales			
0c9e00c70f030158e9fd33a21fdd7e01	11	1.000	d__Bacteria	p__Gemmatimonadota	c__BD2-11_terrestrial_group	o__BD2-11_terrestrial_group	f__BD2-11_terrestrial_group	g__BD2-11_terrestrial_group	
ba2be41282c68bc0ca3cb31ba0f17fb1	154	1.000	d__Bacteria	p__Actinobacteriota	c__Acidimicrobiia	o__Actinomarinales	f__uncultured	g__uncultured	s__uncultured_actinobacterium
d5127d3631a165fa06b942e8f1726597	4	1.000	d__Bacteria	p__PAUC34f	c__PAUC34f	o__PAUC34f	f__PAUC34f	g__PAUC34f	s__uncultured_bacterium
0afae59ac7660ed8945f40486890a686	55	1.000	d__Bacteria	p__Proteobacteria	c__Gammaproteobacteria	o__Pseudomonadales	f__KI89A_clade	g__KI89A_clade	s__uncultured_bacterium
308b149b607d495174541fb08ba2c10c	23	1.000	d__Bacteria	p__Proteobacteria	c__Gammaproteobacteria	o__Steroidobacterales	f__Woeseiaceae	g__JTB255_marine_benthic_group	s__uncultured_bacterium
f54de3234d2ae114686b3ab4377ad320	4	1.000	d__Bacteria	p__Chloroflexi	c__Anaerolineae	o__Caldilineales	f__Caldilineaceae	g__uncultured	s__uncultured_bacterium
67d32d9ad1b8d9919894a6b554d0742f	81	1.000	d__Bacteria	p__Acidobacteriota	c__Thermoanaerobaculia	o__Thermoanaerobaculales	f__Thermoanaerobaculaceae	g__Subgroup_10	s__uncultured_bacterium
04ead0e7f6466e35395823079cef35f4	121	1.000	d__Bacteria	p__Proteobacteria	c__Gammaproteobacteria	o__Steroidobacterales	f__Woeseiaceae	g__JTB255_marine_benthic_group	s__uncultured_bacterium
7eb4eff196bb4dd7b89cbe33a9ce556b	15	1.000	d__Bacteria	p__Proteobacteria	c__Gammaproteobacteria	o__Pseudomonadales	f__KI89A_clade	g__KI89A_clade	s__uncultured_bacterium

f829766bfb6ed4c8e50e44ad3fd7d070	98	1.000	d__Bacteria	p__Nitrospirota	c__Nitrospiria	o__Nitrospirales	f__Nitrospiraceae	g__Nitrospira	s__uncultured_bacterium
5a8c1c9db3c3545353c94d4f478ef15d	295	1.000	d__Bacteria	p__Dadabacteria	c__Dadabacteriia	o__Dadabacteriales	f__Dadabacteriales	g__Dadabacteriales	s__uncultured_delta
0d8f666c087c9f2d3f828cf2f2316539	133	1.000	d__Bacteria	p__Proteobacteria	c__Alphaproteobacteria	o__Puniceispirillales	f__Puniceispirillales_Incertae_Sedis	g__Constrictibacter	s__uncultured_bacterium
b78593b1494dc854b6d5e1a0f7d9fee8	3	1.000	d__Bacteria	p__Poribacteria	c__Poribacteria	o__Poribacteria	f__Poribacteria	g__Poribacteria	s__Candidatus_Poribacteria
1ed19b4056ec7e44e5b92d5c90b7ef74	37	1.000	d__Bacteria	p__Chloroflexi	c__TK17	o__TK17	f__TK17	g__TK17	s__uncultured_Chloroflexi
aa0bb46f8879fdd1efd50ce3d7859c75	24	1.000	d__Bacteria	p__Proteobacteria	c__Gammaproteobacteria	o__Pseudomonadales	f__KI89A_clade	g__KI89A_clade	s__uncultured_bacterium
97b409024eee4b244d9476fd87b2554a	13	1.000	d__Bacteria	p__Acidobacteriota	c__Vicinamibacteria	o__Vicinamibacterales	f__uncultured	g__uncultured	
0e273566d4831ef207b700781fbe28f1	11	1.000	d__Bacteria	p__PAUC34f	c__PAUC34f	o__PAUC34f	f__PAUC34f	g__PAUC34f	s__uncultured_bacterium
af06527ef871bd454b13f89217c46b55	7	1.000	d__Bacteria	p__Proteobacteria	c__Gammaproteobacteria	o__EPR3968-O8a-Bc78	f__EPR3968-O8a-Bc78	g__EPR3968-O8a-Bc78	
1030da6abd9328dce23e008b72290f5c	24	1.000	d__Bacteria	p__Proteobacteria	c__Gammaproteobacteria	o__Nitrosococcales	f__Nitrosococcaceae	g__AqS1	s__uncultured_bacterium
b96b1e589e704b6a643f86890117cfa7	37	1.000	d__Bacteria	p__Nitrospirota	c__Nitrospiria	o__Nitrospirales	f__Nitrospiraceae	g__Nitrospira	s__uncultured_bacterium
b959053936fe72856bac9724c29645b3	628	1.000	d__Bacteria	p__Chloroflexi	c__Anaerolineae	o__Caldilineales	f__Caldilineaceae	g__uncultured	s__uncultured_Chloroflexus
d6a79416c0b360cb1c7da2b29c779121	340	1.000	d__Bacteria	p__Verrucomicrobiota	c__Verrucomicrobiae	o__Opitutales	f__Puniceicoccaceae	g__Cerasicoccus	s__uncultured_Verrucomicrobia
e1731bd5d1e591d09317ee220ce0d3e7	93	1.000	d__Bacteria	p__Chloroflexi	c__Dehalococcoidia	o__SAR202_clade	f__SAR202_clade	g__SAR202_clade	

2752fe0df9c81ebd07020c5ea74aecd4	12	1.000	d__Bacteria	p__PAUC34f	c__PAUC34f	o__PAUC34f	f__PAUC34f	g__PAUC34f	s__uncultured_bacterium
fc874f067345bcf51a64fcd59a91f979	4	1.000	d__Bacteria	p__Spirochaetota	c__Spirochaetia	o__Spirochaetales	f__Spirochaetaceae	g__Spirochaeta	s__uncultured_bacterium
181d1408aecc1a723e331d70f44d19b7	66	1.000	d__Bacteria	p__Proteobacteria	c__Gammaproteobacteria	o__Pseudomonadales	f__KI89A_clade	g__KI89A_clade	s__uncultured_bacterium
45d1ef31c578b23707b0774757a7be7a	18	1.000	d__Bacteria	p__Gemmatimonadota	c__PAUC43f_marine_benthic_group	o__PAUC43f_marine_benthic_group	f__PAUC43f_marine_benthic_group	g__PAUC43f_marine_benthic_group	s__uncultured_bacterium
06e8de7846e177c345d2be531fa65c9d	65	1.000	d__Bacteria	p__Proteobacteria	c__Gammaproteobacteria	o__Pseudomonadales	f__KI89A_clade	g__KI89A_clade	s__uncultured_bacterium
2b22019b177e749a27094b57a0af16f7	24	1.000	d__Bacteria	p__Acidobacteriota	c__Vicinamibacteria	o__Vicinamibacterales	f__uncultured	g__uncultured	
7ecd5863d83ddec7810019b10d22be2b	8	1.000	d__Bacteria	p__Proteobacteria	c__Gammaproteobacteria	o__UBA10353_marine_group	f__UBA10353_marine_group	g__UBA10353_marine_group	
b85f1126d6929768b78d7f687e715ee8	11	1.000	d__Bacteria	p__AncK6	c__AncK6	o__AncK6	f__AncK6	g__AncK6	s__uncultured_bacterium
d7c5f473f6373dc98492829af4241f42	24	1.000	d__Bacteria	p__Chloroflexi	c__Dehalococcoidia	o__SAR202_clade	f__SAR202_clade	g__SAR202_clade	
315ca649e9266db51e286539b0cc4318	1194	1.000	d__Bacteria	p__Chloroflexi	c__Anaerolineae	o__Caldilineales	f__Caldilineaceae	g__uncultured	s__uncultured_Chloroflexus
cc2d9d512e0c838daeada1dbb4c05718	5	1.000	d__Bacteria	p__Planctomycetota	c__Planctomycetes	o__Planctomycetales	f__Rubinisphaeraceae	g__Planctomicrobium	s__uncultured_bacterium
646f7c455e57aae62d08c37f43e1d009	5	1.000	d__Bacteria	p__Chloroflexi	c__Dehalococcoidia	o__SAR202_clade	f__SAR202_clade	g__SAR202_clade	
8e2b88fa6680528734b9570ffd855be8	873	1.000	d__Bacteria	p__Chloroflexi	c__Anaerolineae	o__Caldilineales	f__Caldilineaceae	g__uncultured	s__uncultured_Chloroflexus

bbb117a61fc7435472f2cd015fbcd7c0	2	1.000	d__Bacteria	p__Myxococcota	c__Myxococcia	o__Myxococcales	f__Myxococcaceae	g__P3OB-42	
f91497caeef1f551951e0b96baacf50a	2	1.000	d__Bacteria	p__Planctomycetota	c__OM190	o__OM190	f__OM190	g__OM190	
d396b0b3936c69e52cfa76fb6d8a0f83	12	1.000	d__Bacteria	p__Proteobacteria	c__Alphaproteobacteria	o__Rhizobiales	f__Rhizobiales_Incertae_Sedis	g__Anderseniella	s__uncultured_bacterium
712869e9bf8bcbe9e2e4d535a1e3db0b	49	1.000	d__Bacteria	p__Proteobacteria	c__Gammaproteobacteria	o__Pseudomonadales	f__KI89A_clade	g__KI89A_clade	
7acea8299e26f7d51b0953e29ed4a686	203	1.000	d__Bacteria	p__Chloroflexi	c__Anaerolineae	o__Caldilineales	f__Caldilineaceae	g__uncultured	s__uncultured_Chloroflexus
80214b75450e4d53893b0f57d371a6fc	158	1.000	d__Bacteria	p__Chloroflexi	c__Dehalococcoidia	o__SAR202_clade	f__SAR202_clade	g__SAR202_clade	
7be2632751b6fce52f846e031c9b99b4	582	1.000	d__Bacteria	p__Acidobacteriota	c__Acidobacteriae	o__PAUC26f	f__PAUC26f	g__PAUC26f	s__uncultured_bacterium
863382f82e439c69fba5f72df01794b8	72	1.000	d__Bacteria	p__Acidobacteriota	c__Vicinamibacteria	o__Vicinamibacterales	f__uncultured	g__uncultured	
5739abb396ff2b17a0d0973ad82ea585	64	1.000	d__Bacteria	p__Chloroflexi	c__Dehalococcoidia	o__SAR202_clade	f__SAR202_clade	g__SAR202_clade	
710b8460da5d5ba63fb583278ef8b4ff	14	1.000	d__Bacteria	p__Chloroflexi	c__Dehalococcoidia	o__SAR202_clade	f__SAR202_clade	g__SAR202_clade	
964bec32fd04991db6e033573c4667ac	266	1.000	d__Bacteria	p__Chloroflexi	c__TK17	o__TK17	f__TK17	g__TK17	s__uncultured_Chloroflexi
03979a791e670e16043dd761d33a39ac	17	1.000	d__Bacteria	p__Chloroflexi	c__TK17	o__TK17	f__TK17	g__TK17	s__uncultured_Chloroflexi
ab46399cc898e26437ea05f7fc39510f	9	1.000	d__Bacteria	p__Chloroflexi	c__TK17	o__TK17	f__TK17	g__TK17	s__uncultured_Chloroflexi
6b500b782f42c0d35daa56a0888d1c56	78	1.000	d__Bacteria	p__Acidobacteriota	c__Acidobacteriae	o__PAUC26f	f__PAUC26f	g__PAUC26f	s__uncultured_bacterium

d0eb0907c8577d89b61cbd56303e39cb	3	1.000	d__Bacteria	p__Poribacteria	c__Poribacteria	o__Poribacteria	f__Poribacteria	g__Poribacteria	
85f72f312b6ced96a68e9cce2fdaf2c9	288	1.000	d__Bacteria	p__Acidobacteriota	c__Acidobacteriae	o__PAUC26f	f__PAUC26f	g__PAUC26f	s__uncultured_bacterium
cbafa34b4b5463b2758ad307c53006e7	11	1.000	d__Bacteria	p__Chloroflexi	c__Dehalococcoidia	o__SAR202_clade	f__SAR202_clade	g__SAR202_clade	
51c7d72144a61a65ce82683be435951e	9	1.000	d__Bacteria	p__Chloroflexi	c__Dehalococcoidia	o__SAR202_clade	f__SAR202_clade	g__SAR202_clade	
d9fba8f27971c65a9b820cf7ffa0c1af	41	1.000	d__Bacteria	p__Proteobacteria	c__Gammaproteobacteria	o__Pseudomonadales	f__KI89A_clade	g__KI89A_clade	
f92ba592616a54b5cf690fef09b6c03f	20	1.000	d__Bacteria	p__Chloroflexi	c__TK30	o__TK30	f__TK30	g__TK30	s__uncultured_bacterium
d7b64272a88f8593e4747d58eb4b5c64	12	1.000	d__Bacteria	p__Chloroflexi	c__Dehalococcoidia	o__SAR202_clade	f__SAR202_clade	g__SAR202_clade	
36efecc5394efd07b3541546b4edd7f7	12	1.000	d__Bacteria	p__Deinococcota	c__Deinococci	o__Deinococcales	f__Trueperaceae	g__Truepera	s__uncultured_Truepera
f666a59c136d4c02014b23acca2cd2a7	88	1.000	d__Bacteria	p__Chloroflexi	c__TK30	o__TK30	f__TK30	g__TK30	s__uncultured_bacterium
2d77290bdffb556285d2fc8ee564ecb0	6	1.000	d__Bacteria	p__Proteobacteria	c__Gammaproteobacteria	o__Pseudomonadales	f__OM182_clade	g__OM182_clade	
11fe107d7fb62f0947861e1dfb050164	4	1.000	d__Bacteria	p__NB1-j	c__NB1-j	o__NB1-j	f__NB1-j	g__NB1-j	
e30d6ed8dad0da58161077d97b4b982d	10	1.000	d__Bacteria	p__Chloroflexi	c__TK17	o__TK17	f__TK17	g__TK17	
08cb050dd40bc03765795a14f62098b2	11	1.000	d__Bacteria	p__Verrucomicrobiota	c__Verrucomicrobiae	o__Verrucomicrobiales	f__DEV007	g__DEV007	
707fe796f8fa5c4d105f98efb633be38	93	1.000	d__Bacteria	p__Poribacteria	c__Poribacteria	o__Poribacteria	f__Poribacteria	g__Poribacteria	s__Candidatus_Poribacteria

1c9c1ed19c6d51c34fb0ca057f53c4c6	266	1.000	d__Bacteria	p__Chloroflexi	c__Dehalococcoidia	o__SAR202_clade	f__SAR202_clade	g__SAR202_clade	
d82afeb1f269e2e6a6a26f949c334ed0	68	1.000	d__Bacteria	p__Chloroflexi	c__Dehalococcoidia	o__SAR202_clade	f__SAR202_clade	g__SAR202_clade	
24c5fdfe34d67667a4135852e7001503	4	1.000	d__Bacteria	p__Verrucomicrobiota	c__Verrucomicrobiae	o__Verrucomicrobiales	f__DEV007	g__DEV007	
374255d4f186035aeeab8644d17324ac	130	1.000	d__Bacteria	p__Poribacteria	c__Poribacteria	o__Poribacteria	f__Poribacteria	g__Poribacteria	s__Candidatus_Poribacteria
2dd77cc0448701cd23bc91ae10efe769	104	1.000	d__Bacteria	p__Chloroflexi	c__Anaerolineae	o__Caldilineales	f__Caldilineaceae	g__uncultured	s__uncultured_Chloroflexus
32d98b8671b53afa27ac858bb09354b0	3	1.000	d__Bacteria	p__Chloroflexi	c__Anaerolineae	o__Ardenticatenales	f__Ardenticatenaceae	g__uncultured	s__uncultured_Chloroflexi
13a7e2656777d022399c4a3a717f4f6b	33	1.000	d__Bacteria	p__Gemmatimonadota	c__BD2-11_terrestrial_group	o__BD2-11_terrestrial_group	f__BD2-11_terrestrial_group	g__BD2-11_terrestrial_group	
5a2e587ac3fc8064fe70bb3592c72f69	19	1.000	d__Bacteria	p__Acidobacteriota	c__Subgroup_11	o__Subgroup_11	f__Subgroup_11	g__Subgroup_11	s__uncultured_bacterium
958ecd59951d0ee9fffe7e5f4705b9e7	51	1.000	d__Bacteria	p__Gemmatimonadota	c__BD2-11_terrestrial_group	o__BD2-11_terrestrial_group	f__BD2-11_terrestrial_group	g__BD2-11_terrestrial_group	
581a66aaa68ddedda604b324cb2fb586	13	1.000	d__Bacteria	p__Gemmatimonadota	c__BD2-11_terrestrial_group	o__BD2-11_terrestrial_group	f__BD2-11_terrestrial_group	g__BD2-11_terrestrial_group	
204039ee539ee4803e113e869ad8d1ec	30	1.000	d__Bacteria	p__Poribacteria	c__Poribacteria	o__Poribacteria	f__Poribacteria	g__Poribacteria	

Table S3. ASVs (136) from *Geodia cydonium* collected in Mar Piccolo with percentage of confidence ≥ 75%.

ASVs ID	3	Confidence	Domain	Phylum	Class	Order	Family	Genus	Species
9da8745df8f0bb2123fdd2e3b01a1a12	14	0.750	d__Bacteria	p__Chloroflexi	c__Dehalococcoidia	o__SAR202_clade	f__SAR202_clade	g__SAR202_clade	s__uncultured_marine
ac08176087f3f95982f3512fdeb4434c	3	0.765	d__Bacteria	p__SAR324_clade(Marine_group_B)	c__SAR324_clade(Marine_group_B)	o__SAR324_clade(Marine_group_B)	f__SAR324_clade(Marine_group_B)	g__SAR324_clade(Marine_group_B)	s__uncultured_bacterium
587240fbd10a47e8b2e9740c3dd19984	3	0.770	d__Bacteria	p__Chloroflexi	c__Anaerolineae	o__SBR1031	f__SBR1031	g__SBR1031	
8d40d3ef21b9f19aefb3bb62a2a1c500	3	0.774	d__Bacteria	p__SAR324_clade(Marine_group_B)	c__SAR324_clade(Marine_group_B)	o__SAR324_clade(Marine_group_B)	f__SAR324_clade(Marine_group_B)	g__SAR324_clade(Marine_group_B)	s__uncultured_bacterium
8db96cf9b4b1d51c531b6585c07e341f	3	0.787	d__Bacteria	p__Acidobacteriota	c__Vicinamibacteria	o__Subgroup_9	f__Subgroup_9	g__Subgroup_9	s__uncultured_marine
6b1a3cf3e6cfce227010e7f34a9580a1	183	0.806	d__Bacteria	p__Actinobacteriota	c__Acidimicrobiia	o__Microtrichales	f__Microtrichaceae	g__Sva0996_marine_group	s__uncultured_bacterium
e4fdf0e7ebfaea088db23e6d94347ede	49	0.811	d__Bacteria	p__Proteobacteria	c__Gammaproteobacteria	o__Pseudomonadales	f__OM182_clade	g__OM182_clade	s__uncultured_gamma
798986fed73ab850f3cb13e8182c884c	85	0.814	d__Bacteria	p__Chloroflexi	c__Dehalococcoidia	o__SAR202_clade	f__SAR202_clade	g__SAR202_clade	s__uncultured_bacterium
c0e06c13527a7df33b251bef74eb6413	25	0.816	d__Bacteria	p__Proteobacteria	c__Gammaproteobacteria	o__Pseudomonadales	f__Endozoicomonadaceae	g__Endozoicomonas	s__uncultured_Spongiobacter
0d37ba9129d041723a27210ef1ee6440	2	0.818	d__Bacteria	p__Acidobacteriota	c__Thermoanaerobaculia	o__Thermoanaerobaculales	f__Thermoanaerobaculaceae	g__Subgroup_10	s__uncultured_bacterium
063cce8658f9e598aa3a032f35ab3260	29	0.821	d__Bacteria	p__Chloroflexi	c__JG30-KF-CM66	o__JG30-KF-CM66	f__JG30-KF-CM66	g__JG30-KF-CM66	s__uncultured_bacterium
a9fa50ca9c3c109ddad3d3246312065e	4	0.825	d__Bacteria	p__Proteobacteria	c__Gammaproteobacteria	o__HOC36	f__HOC36	g__HOC36	s__uncultured_bacterium
5cc6c96f1cd29bcf037b578b9d8f53e0	2	0.827	d__Bacteria	p__Proteobacteria	c__Gammaproteobacteria	o__Acidithiobacillales	f__Acidithiobacillaceae	g__KCM-B-112	s__uncultured_gamma
0d234efa5aabd14c58a506f8748c6b02	1919	0.828	d__Bacteria	p__Gemmatimonadota	c__BD2-11_terrestrial_group	o__BD2-11_terrestrial_group	f__BD2-11_terrestrial_group	g__BD2-11_terrestrial_group	s__uncultured_delta

8fa45acf2e34400e7436b7e195f126cb	41	0.829	d__Bacteria	p__Gemmatimonadota	c__BD2-11_terrestrial_group	o__BD2-11_terrestrial_group	f__BD2-11_terrestrial_group	g__BD2-11_terrestrial_group	s__uncultured_bacterium
6c5d74165cd009d17fa96d1b8298b540	41	0.841	d__Bacteria	p__Bacteroidota	c__Rhodothermia	o__Rhodothermales	f__Rhodothermaceae	g__uncultured	s__uncultured_bacterium
d1b3ed6e18de40d28dcdcd4e739cfe4d	195	0.846	d__Bacteria	p__Actinobacteriota	c__Acidimicrobiia	o__Microtrichales	f__Microtrichaceae	g__Sva0996_marine_group	s__uncultured_actinobacterium
2bfd7ec7f83eb3eaf972c29e82af0ab4	14	0.853	d__Bacteria	p__Proteobacteria	c__Alphaproteobacteria	o__Rhodobacterales	f__Rhodobacteraceae	g__Ruegeria	s__Ruegeria_sp.
a4a2b3ac9ec1333d2c8b5c90e9717ac6	21	0.864	d__Bacteria	p__Chloroflexi	c__Dehalococcoidia	o__SAR202_clade	f__SAR202_clade	g__SAR202_clade	s__uncultured_bacterium
75bc272b5d1abeecfbcbc496c184e0b9	2	0.868	d__Bacteria	p__Proteobacteria	c__Alphaproteobacteria	o__Defluviicoccales	f__uncultured	g__uncultured	s__uncultured_bacterium
fa8214e24a8e3f1b899dbc35a250182a	5	0.877	d__Bacteria	p__Actinobacteriota	c__Acidimicrobiia	o__Microtrichales	f__Microtrichaceae	g__Sva0996_marine_group	s__uncultured_bacterium
fd17b3b166715bdb837bcbaaeefcd2c3	84	0.884	d__Bacteria	p__Chloroflexi	c__JG30-KF-CM66	o__JG30-KF-CM66	f__JG30-KF-CM66	g__JG30-KF-CM66	s__uncultured_Chloroflexi
9d8d12932083466e65c9214e93a11e74	20	0.887	d__Bacteria	p__Proteobacteria	c__Alphaproteobacteria	o__Rhodobacterales	f__Rhodobacteraceae	g__uncultured	
97fb8ec1addf7f02c1b8ff7f49627563	106	0.888	d__Bacteria	p__Proteobacteria	c__Gammaproteobacteria	o__HOC36	f__HOC36	g__HOC36	s__uncultured_gamma
99367a8d61b5ce0c6b6d58109d5936f4	1	0.888	d__Bacteria	p__Nitrospinota	c__P9X2b3D02	o__P9X2b3D02	f__P9X2b3D02	g__P9X2b3D02	
04b360a26fed057128b32ccb4ebf9c0c	120	0.890	d__Bacteria	p__Proteobacteria	c__Alphaproteobacteria	o__Defluviicoccales	f__uncultured	g__uncultured	s__uncultured_bacterium
88b6d081caec18a35df2037295886130	22	0.891	d__Bacteria	p__Chloroflexi	c__Anaerolineae	o__SBR1031	f__A4b	g__A4b	s__uncultured_Chloroflexus
d0ac5be8625109ccddadbc20b7c0be0a	26	0.896	d__Bacteria	p__Chloroflexi	c__JG30-KF-CM66	o__JG30-KF-CM66	f__JG30-KF-CM66	g__JG30-KF-CM66	s__uncultured_Chloroflexi
05570da352ff8bd242da1548e1db25fc	114	0.900	d__Bacteria	p__Chloroflexi	c__Dehalococcoidia	o__SAR202_clade	f__SAR202_clade	g__SAR202_clade	s__uncultured_bacterium
6643b4422f07bc96e140c6cc5bec7bc3	77	0.901	d__Bacteria	p__Poribacteria	c__Poribacteria	o__Poribacteria	f__Poribacteria	g__Poribacteria	s__Candidatus_Poribacteria
0afe9f04fb31196f59b6f0853098bcff	203	0.907	d__Bacteria	p__Actinobacteriota	c__Acidimicrobiia	o__Microtrichales	f__Microtrichaceae	g__Sva0996_marine_group	s__uncultured_bacterium
cf8c413052193af977287d9da18c4d39	51	0.909	d__Bacteria	p__Chloroflexi	c__Anaerolineae	o__SBR1031	f__A4b	g__A4b	s__uncultured_Chloroflexus
c4f5adcf981a60fe5fd13da1f679b923	38	0.928	d__Bacteria	p__Acidobacteriota	c__Vicinamibacteria	o__Vicinamibacterales	f__uncultured	g__uncultured	s__uncultured_bacterium

7002c643193e83563c91d1e6ffc44302	544	0.928	d__Bacteria	p__Gemmatimonadota	c__BD2-11_terrestrial_group	o__BD2-11_terrestrial_group	f__BD2-11_terrestrial_group	g__BD2-11_terrestrial_group	s__uncultured_bacterium
e4fc3333123c849329d7675c66df815a	80	0.934	d__Bacteria	p__Proteobacteria	c__Alphaproteobacteria	o__Defluviicoccales	f__uncultured	g__uncultured	s__uncultured_bacterium
a97eae53c423c75d5bf421e68fd34ccd	97	0.938	d__Bacteria	p__Chloroflexi	c__Dehalococcoidia	o__SAR202_clade	f__SAR202_clade	g__SAR202_clade	s__uncultured_SAR202
49ff42c5604b287043f0b0f96f1edaf0	4	0.945	d__Bacteria	p__Proteobacteria	c__Gammaproteobacteria	o__EPR3968-O8a-Bc78	f__EPR3968-O8a-Bc78	g__EPR3968-O8a-Bc78	s__uncultured_bacterium
ad046607ad83e9d65bce50bd1f0c4646	21	0.950	d__Bacteria	p__Actinobacteriota	c__Acidimicrobiia	o__Microtrichales	f__Microtrichaceae	g__Sva0996_marine_group	s__uncultured_bacterium
93f5f5ab29e68f8b03e12317721caa7a	13	0.951	d__Bacteria	p__Entotheonellaeota	c__Entotheonellia	o__Entotheonellales	f__Entotheonellaceae	g__Entotheonellaceae	s__uncultured_delta
b298f798b8be15a6f9b2218399a28542	3	0.956	d__Bacteria	p__Proteobacteria	c__Gammaproteobacteria	o__HOC36	f__HOC36	g__HOC36	
4c31d6515474234ca32b8c4ba923f87a	86	0.957	d__Bacteria	p__Acidobacteriota	c__Vicinamibacteria	o__Vicinamibacterales	f__uncultured	g__uncultured	s__uncultured_bacterium
f9404661ca724985ec4de10ad921336f	198	0.959	d__Bacteria	p__Proteobacteria	c__Gammaproteobacteria	o__EPR3968-O8a-Bc78	f__EPR3968-O8a-Bc78	g__EPR3968-O8a-Bc78	s__uncultured_bacterium
57f503246c1f0a094b5042b7ac19ba17	83	0.960	d__Bacteria	p__Actinobacteriota	c__Acidimicrobiia	o__Microtrichales	f__Microtrichaceae	g__Sva0996_marine_group	s__uncultured_bacterium
46e3f7707998a111594175bfb2597382	2	0.960	d__Bacteria	p__Gemmatimonadota	c__BD2-11_terrestrial_group	o__BD2-11_terrestrial_group	f__BD2-11_terrestrial_group	g__BD2-11_terrestrial_group	s__uncultured_delta
26ca81e16c83364941449007f0abf2c9	3	0.961	d__Bacteria	p__Chloroflexi	c__TK30	o__TK30	f__TK30	g__TK30	s__uncultured_bacterium
1f4fe56a542b451272bfffbfc4447b93	29	0.961	d__Bacteria	p__Nitrospinota	c__P9X2b3D02	o__P9X2b3D02	f__P9X2b3D02	g__P9X2b3D02	s__uncultured_bacterium
eb8e136acd9343a1e960022c06dfb5e4	50	0.962	d__Bacteria	p__Proteobacteria	c__Gammaproteobacteria	o__Pseudomonadales	f__Halieaceae		
cd7a605b7c7129c35c2b41a2c84441bb	20	0.962	d__Bacteria	p__Chloroflexi	c__Dehalococcoidia	o__S085	f__S085	g__S085	s__uncultured_bacterium
6d8946962640d80fe1ce1bb60f069b6e	2	0.966	d__Bacteria	p__Chloroflexi	c__Dehalococcoidia	o__SAR202_clade	f__SAR202_clade	g__SAR202_clade	s__uncultured_bacterium
e8747b6d96d329b5dceb0028f993cae8	7	0.969	d__Bacteria	p__Nitrospinota	c__P9X2b3D02	o__P9X2b3D02	f__P9X2b3D02	g__P9X2b3D02	s__uncultured_bacterium

8c5943fd1fd05d69baecaa0f89c1c263	3	0.970	d__Bacteria	p__Proteobacteria	c__Gammaproteobacteria	o__Pseudomonadales	f__KI89A_clade	g__KI89A_clade	s__uncultured_bacterium
6264b0987edc751d298c956711bc97ce	1	0.970	d__Bacteria	p__Proteobacteria	c__Alphaproteobacteria	o__Rhodospirillales	f__AEGEAN-169_marine_group	g__AEGEAN-169_marine_group	s__metagenome
40c2b630e6829314f805e1d934951f02	108	0.972	d__Bacteria	p__Bacteroidota	c__Rhodothermia	o__Rhodothermales	f__Rhodothermaceae	g__uncultured	s__uncultured_bacterium
ae62a75860daa684faffc303ea84af53	53	0.972	d__Bacteria	p__Gemmatimonadota	c__BD2-11_terrestrial_group	o__BD2-11_terrestrial_group	f__BD2-11_terrestrial_group	g__BD2-11_terrestrial_group	s__uncultured_bacterium
a77d4a4673f37a324bff74e694e56e4f	2	0.972	d__Bacteria	p__PAUC34f	c__PAUC34f	o__PAUC34f	f__PAUC34f	g__PAUC34f	
c1bc5c783371c11161d56cf7b40e3828	130	0.972	d__Bacteria	p__Proteobacteria	c__Gammaproteobacteria	o__Pseudomonadales	f__Pseudohongiellaceae	g__Pseudohongiella	s__uncultured_bacterium
1fca9214ea64502cf023fa6372a8498d	148	0.976	d__Bacteria	p__Proteobacteria	c__Alphaproteobacteria	o__Kiloniellales	f__Kiloniellaceae	g__uncultured	s__uncultured_bacterium
bf7c48db5b7f62245f4e6507542ae90d	37	0.976	d__Bacteria	p__Actinobacteriota	c__Acidimicrobiia	o__Microtrichales	f__Microtrichaceae	g__Sva0996_marine_group	s__uncultured_bacterium
d4ae7081f0fb816b16a68049b1f5381d	8	0.978	d__Bacteria	p__Proteobacteria	c__Gammaproteobacteria	o__UBA10353_marine_group	f__UBA10353_marine_group	g__UBA10353_marine_group	s__uncultured_bacterium
318c3c6a14aed9d629284ae6c933bc18	529	0.980	d__Bacteria	p__Actinobacteriota	c__Acidimicrobiia	o__Microtrichales	f__Microtrichaceae	g__Sva0996_marine_group	s__uncultured_bacterium
e68d370d47f1c36c771646ca1ad728ff	56	0.980	d__Bacteria	p__Actinobacteriota	c__Acidimicrobiia	o__Microtrichales	f__Microtrichaceae	g__Sva0996_marine_group	s__uncultured_actinobacterium
51ea2cda562169e7b6ddc12ccf957ef3	141	0.984	d__Bacteria	p__Chloroflexi	c__Dehalococcoidia	o__SAR202_clade	f__SAR202_clade	g__SAR202_clade	s__uncultured_SAR202
187bdf5cebe94820fee030f095b6e5a4	4	0.985	d__Bacteria	p__Chloroflexi	c__Anaerolineae	o__SBR1031	f__A4b	g__A4b	s__uncultured_Chloroflexi
0b634770abf69b42997028d717039878	5	0.986	d__Bacteria	p__Chloroflexi	c__Dehalococcoidia	o__SAR202_clade	f__SAR202_clade	g__SAR202_clade	s__uncultured_bacterium
6ce359edd2145bb37b0fe5100c5682b3	18	0.986	d__Bacteria	p__Proteobacteria	c__Gammaproteobacteria	o__EPR3968-O8a-Bc78	f__EPR3968-O8a-Bc78	g__EPR3968-O8a-Bc78	s__uncultured_bacterium
a5f252c77ba9ec05e3210792c7ad8a69	186	0.989	d__Bacteria	p__Proteobacteria	c__Gammaproteobacteria	o__EPR3968-O8a-Bc78	f__EPR3968-O8a-Bc78	g__EPR3968-O8a-Bc78	s__uncultured_bacterium

47d9f4875cab3926fa511019c3c38e9c	12	0.990	d__Bacteria	p__Proteobacteria	c__Gammaproteobacteria	o__Pseudomonadales	f__KI89A_clade	g__KI89A_clade	s__uncultured_organism
896038f30a5ec46eabb565fd88b17228	17	0.991	d__Bacteria	p__Nitrospinota	c__P9X2b3D02	o__P9X2b3D02	f__P9X2b3D02	g__P9X2b3D02	s__uncultured_bacterium
f8001f9bc1459e220983121817265230	313	0.993	d__Bacteria	p__Chloroflexi	c__Anaerolineae	o__Caldilineales	f__Caldilineaceae	g__uncultured	s__uncultured_Caldilinea
0d864eef2bc2c0e980088ad43d873c36	82	0.994	d__Bacteria	p__Actinobacteriota	c__Acidimicrobiia	o__Microtrichales	f__Microtrichaceae	g__Sva0996_marine_group	s__uncultured_actinobacterium
a70458ef51bee113c903db03b8cd2651	5	0.994	d__Bacteria	p__Proteobacteria	c__Alphaproteobacteria	o__AT-s3-44	f__AT-s3-44	g__AT-s3-44	s__uncultured_bacterium
b335c839d0eeb10db2b0c527a8015a4d	14	0.994	d__Bacteria	p__Proteobacteria	c__Gammaproteobacteria	o__pItb-vmat-80	f__pItb-vmat-80	g__pItb-vmat-80	s__uncultured_bacterium
bc910e00e7a316d2ca226147f62a2f3d	5	0.994	d__Bacteria	p__Acidobacteriota	c__Thermoanaerobaculia	o__Thermoanaerobaculales	f__Thermoanaerobaculaceae	g__Subgroup_10	s__uncultured_bacterium
742ced2a280d3872e2b5442c2a777ca1	2	0.995	d__Bacteria	p__Cyanobacteria	c__Cyanobacteriia	o__Synechococcales	f__Cyanobiaceae	g__Cyanobium_PCC-6307	s__uncultured_bacterium
5a8202ac1cdf3719a1bebb1d7f3a03c6	5	0.995	d__Bacteria	p__Gemmatimonadota	c__BD2-11_terrestrial_group	o__BD2-11_terrestrial_group	f__BD2-11_terrestrial_group	g__BD2-11_terrestrial_group	s__uncultured_delta
c6237bc0c7a60bd8fc149c5c7fecba7f	321	0.995	d__Bacteria	p__Actinobacteriota	c__Acidimicrobiia	o__Actinomarinales	f__uncultured	g__uncultured	s__uncultured_actinobacterium
f74bf88be3c3fc3aa089270adfe9964d	76	0.995	d__Bacteria	p__Proteobacteria	c__Gammaproteobacteria	o__JTB23	f__JTB23	g__JTB23	s__uncultured_proteobacterium
dd4ec1bdb423917dc44d526d75dc08ed	64	0.995	d__Bacteria	p__Chloroflexi	c__Dehalococcoidia	o__SAR202_clade	f__SAR202_clade	g__SAR202_clade	s__uncultured_bacterium
3932d0fa1f1d94a503cc7e2650d396e7	36	0.996	d__Bacteria	p__Actinobacteriota	c__Acidimicrobiia	o__Actinomarinales	f__uncultured	g__uncultured	
0492e7d534276b00f14cf30b1e9b3bc4	36	0.996	d__Bacteria	p__Proteobacteria	c__Gammaproteobacteria	o__JTB23	f__JTB23	g__JTB23	
30683b155e582c29678eb269dd6d4157	2	0.997	d__Bacteria	p__Cyanobacteria	c__Cyanobacteriia	o__Synechococcales	f__Cyanobiaceae		
dd11719a30fbe604b9b732d2b86f0699	12	0.997	d__Bacteria	p__Proteobacteria	c__Gammaproteobacteria	o__Nitrosococcales	f__Nitrosococcaceae	g__AqS1	s__uncultured_bacterium

f04e69777be21fded a7609b2336ffdf0	5	0.997	d__Bacteria	p__Acidobacteriota	c__Vicinamibacteria	o__Subgroup_9	f__Subgroup_9	g__Subgroup_9	
837089e5175b2b18 1227d6fac488da3d	60	0.998	d__Bacteria	p__Proteobacteria	c__Alphaproteobacteria	o__Puniceispirillales	f__EF100-94H03	g__EF100-94H03	s__uncultured_bacterium
6c809937e62b7b9e 3d9680dde8514458	15	0.998	d__Bacteria	p__Proteobacteria	c__Gammaproteobacteria	o__pItb-vmat-80	f__pItb-vmat-80	g__pItb-vmat-80	s__uncultured_bacterium
d1aed632bbc15502 75ad75f481fc5205	15	0.998	d__Bacteria	p__Actinobacteriota	c__Acidimicrobiia	o__Actinomarinales	f__uncultured	g__uncultured	s__uncultured_actinobacterium
052ace1004921da1 6db003cbafc2cfee	58	0.998	d__Bacteria	p__Proteobacteria	c__Gammaproteobacteria	o__UBA10353_marine_group	f__UBA10353_marine_group	g__UBA10353_marine_group	s__uncultured_marine
af10a20a19fb941c5 021ee854d0b68e3	42	0.998	d__Bacteria	p__Proteobacteria	c__Gammaproteobacteria	o__JTB23	f__JTB23	g__JTB23	
5b182864d2954f96 269ba4a6af1de6c1	36	0.998	d__Bacteria	p__Proteobacteria	c__Alphaproteobacteria	o__Rhodobacterales	f__Rhodobacteraceae	g__Silicimonas	s__uncultured_bacterium
39ad9d5e1d861b89 25bffb1082e586f5	267	0.998	d__Bacteria	p__Acidobacteriota	c__Vicinamibacteria	o__Subgroup_9	f__Subgroup_9	g__Subgroup_9	s__uncultured_bacterium
dcc2f5773af0b7d35 62e8d5a24d6265c	6	0.999	d__Bacteria	p__Myxococcota	c__bacteriap25	o__bacteriap25	f__bacteriap25	g__bacteriap25	s__uncultured_delta
cf3b42c97010fcf89 52173c83b20f8c4	58	0.999	d__Bacteria	p__Proteobacteria	c__Gammaproteobacteria	o__JTB23	f__JTB23	g__JTB23	
014e17b29a1c01cae 4ff773cca164a1c	102	0.999	d__Bacteria	p__Actinobacteriota	c__Acidimicrobiia	o__Microtrichales	f__Microtrichaceae	g__Sva0996_marine_group	
b5b0f076cba7d03c4 f90d32eec106ab6	5	0.999	d__Bacteria	p__Gemmatimonadota	c__PAUC43f_marine_benthic_group	o__PAUC43f_marine_benthic_group	f__PAUC43f_marine_benthic_group	g__PAUC43f_marine_benthic_group	s__uncultured_bacterium
f72871b2770cbfacc 1d095d45ff7b3e4	2484	0.999	d__Bacteria	p__Actinobacteriota	c__Acidimicrobiia	o__Microtrichales	f__Microtrichaceae	g__Sva0996_marine_group	
b936c98dae871c62c 562ca5d09bb1809	24	0.999	d__Bacteria	p__Proteobacteria	c__Alphaproteobacteria	o__Rhodospirillales	f__Magnetospiraceae	g__uncultured	s__uncultured_bacterium
d77f6bd395b7c11f9 ba2aa8720131dc9	38	0.999	d__Bacteria	p__Myxococcota	c__bacteriap25	o__bacteriap25	f__bacteriap25	g__bacteriap25	s__uncultured_bacterium
85383c420d3feb018 5f0a45a5bb830d2	133	0.999	d__Bacteria	p__Poribacteria	c__Poribacteria	o__Poribacteria	f__Poribacteria	g__Poribacteria	s__Candidatus_Poribacteria
2fb370908d414cb2 4f17d51fe45aa3ac	8	0.999	d__Bacteria	p__Chloroflexi	c__Anaerolineae	o__Caldilineales	f__Caldilineaceae	g__uncultured	s__uncultured_Chloroflexus

1951a8ea490c809627cea851ef09deec	75	0.999	d__Bacteria	p__Proteobacteria	c__Gammaproteobacteria	o__Pseudomonadales	f__KI89A_clade	g__KI89A_clade	s__uncultured_bacterium
3b665e9788c5a4f03d657e7be6038833	4	0.999	d__Bacteria	p__Nitrospirota	c__Nitrospiria	o__Nitrospirales	f__Nitrospiraceae	g__Nitrospira	s__uncultured bacterium
bc6c3367d907fd689b82909aa7190484	1	1.000	d__Bacteria	p__Verrucomicrobiota	c__Verrucomicrobiae	o__Opitutales	f__Puniceicoccaceae	g__Cerasicoccus	s__uncultured_Verrucomicrobia
a087e2859a5bca997c5d6595c0ac3280	7	1.000	d__Bacteria	p__Cyanobacteria	c__Cyanobacteriia	o__Synechococcales	f__Cyanobiaceae	g__Synechococcus_CC9902	
5eb2f8c392bda4cc59826cef055198b4	4	1.000	d__Bacteria	p__Chloroflexi	c__Anaerolineae	o__Caldilineales	f__Caldilineaceae	g__uncultured	s__uncultured_Chloroflexus
9a3dee015b81ba479f9297af9a452c19	281	1.000	d__Bacteria	p__Chloroflexi	c__Dehalococcoidia	o__SAR202_clade	f__SAR202_clade	g__SAR202_clade	s__uncultured_deep-sea
a7febd04d2c878e674539ac24d0c5c03	22	1.000	d__Bacteria	p__Chloroflexi	c__Dehalococcoidia	o__SAR202_clade	f__SAR202_clade	g__SAR202_clade	s__uncultured_deep-sea
c58a7f85bef1ba5a10fa8392d82dfbd2	6	1.000	d__Bacteria	p__Chloroflexi	c__TK30	o__TK30	f__TK30	g__TK30	s__uncultured_bacterium
2b3c910608cd5689b70603acf62b59c4	3	1.000	d__Bacteria	p__Chloroflexi	c__Dehalococcoidia	o__SAR202_clade	f__SAR202_clade	g__SAR202_clade	
e9137e128559135e1a3cf6b20f59059b	26	1.000	d__Bacteria	p__Proteobacteria	c__Gammaproteobacteria	o__Pseudomonadales	f__KI89A_clade	g__KI89A_clade	s__uncultured_bacterium
ab6151de482049167acb71a2969446f5	153	1.000	d__Bacteria	p__Proteobacteria	c__Alphaproteobacteria	o__Defluviicoccales	f__uncultured	g__uncultured	s__uncultured_bacterium
90ea6258ef90afe11ec8675f4356296d	205	1.000	d__Bacteria	p__Proteobacteria	c__Gammaproteobacteria	o__Steroidobacterales	f__Woeseiaceae	g__JTB255_marine_benthic_group	s__uncultured_bacterium
67d32d9ad1b8d9919894a6b554d0742f	230	1.000	d__Bacteria	p__Acidobacteriota	c__Thermoanaerobaculia	o__Thermoanaerobaculales	f__Thermoanaerobaculaceae	g__Subgroup_10	s__uncultured_bacterium
04ead0e7f6466e35395823079cef35f4	72	1.000	d__Bacteria	p__Proteobacteria	c__Gammaproteobacteria	o__Steroidobacterales	f__Woeseiaceae	g__JTB255_marine_benthic_group	s__uncultured_bacterium
7eb4eff196bb4dd7b89cbe33a9ce556b	9	1.000	d__Bacteria	p__Proteobacteria	c__Gammaproteobacteria	o__Pseudomonadales	f__KI89A_clade	g__KI89A_clade	s__uncultured_bacterium
f829766bfb6ed4c8e50e44ad3fd7d070	372	1.000	d__Bacteria	p__Nitrospirota	c__Nitrospiria	o__Nitrospirales	f__Nitrospiraceae	g__Nitrospira	s__uncultured bacterium
5a8c1c9db3c3545353c94d4f478ef15d	166	1.000	d__Bacteria	p__Dadabacteria	c__Dadabacteriia	o__Dadabacteriales	f__Dadabacteriales	g__Dadabacteriales	s__uncultured_delta

0d8f666c087c9f2d3f828cf2f2316539	520	1.000	d__Bacteria	p__Proteobacteria	c__Alphaproteobacteria	o__Puniceispirillales	f__Puniceispirillales_Incertae_Sedis	g__Constrictibacter	s__uncultured_bacterium
b96b1e589e704b6a643f86890117cfa7	64	1.000	d__Bacteria	p__Nitrospirota	c__Nitrospiria	o__Nitrospirales	f__Nitrospiraceae	g__Nitrospira	s__uncultured_bacterium
35b4dd5114b178277529a8aab246c67a	58	1.000	d__Bacteria	p__PAUC34f	c__PAUC34f	o__PAUC34f	f__PAUC34f	g__PAUC34f	s__uncultured_bacterium
b959053936fe72856bac9724c29645b3	5076	1.000	d__Bacteria	p__Chloroflexi	c__Anaerolineae	o__Caldilineales	f__Caldilineaceae	g__uncultured	s__uncultured_Chloroflexus
d6a79416c0b360cb1c7da2b29c779121	210	1.000	d__Bacteria	p__Verrucomicrobiota	c__Verrucomicrobiae	o__Opitutales	f__Puniceicoccaceae	g__Cerasicoccus	s__uncultured_Verrucomicrobia
fc874f067345bcf51a64fcd59a91f979	3	1.000	d__Bacteria	p__Spirochaetota	c__Spirochaetia	o__Spirochaetales	f__Spirochaetaceae	g__Spirochaeta	s__uncultured_bacterium
c64130d21eaf070c2b730623dd098bbf	13	1.000	d__Bacteria	p__PAUC34f	c__PAUC34f	o__PAUC34f	f__PAUC34f	g__PAUC34f	s__uncultured_bacterium
9b6d3363709badc2cdc004b929d6eea3	11	1.000	d__Bacteria	p__Proteobacteria	c__Alphaproteobacteria	o__Thalassobaculales	f__Nisaeaceae	g__OM75_clade	s__uncultured_Alphaproteobacteria
f5b28b630059b6f704b3926f9ac71356	130	1.000	d__Bacteria	p__Proteobacteria	c__Gammaproteobacteria	o__Steroidobacterales	f__Woeseiaceae	g__JTB255_marine_benthic_group	s__uncultured_bacterium
315ca649e9266db51e286539b0cc4318	34	1.000	d__Bacteria	p__Chloroflexi	c__Anaerolineae	o__Caldilineales	f__Caldilineaceae	g__uncultured	s__uncultured_Chloroflexus
712869e9bf8bcbe9e2e4d535a1e3db0b	29	1.000	d__Bacteria	p__Proteobacteria	c__Gammaproteobacteria	o__Pseudomonadales	f__KI89A_clade	g__KI89A_clade	
7be2632751b6fce52f846e031c9b99b4	105	1.000	d__Bacteria	p__Acidobacteriota	c__Acidobacteriae	o__PAUC26f	f__PAUC26f	g__PAUC26f	s__uncultured_bacterium
863382f82e439c69fba5f72df01794b8	25	1.000	d__Bacteria	p__Acidobacteriota	c__Vicinamibacteria	o__Vicinamibacterales	f__uncultured	g__uncultured	
964bec32fd04991db6e033573c4667ac	36	1.000	d__Bacteria	p__Chloroflexi	c__TK17	o__TK17	f__TK17	g__TK17	s__uncultured_Chloroflexi
ab46399cc898e26437ea05f7fc39510f	248	1.000	d__Bacteria	p__Chloroflexi	c__TK17	o__TK17	f__TK17	g__TK17	s__uncultured_Chloroflexi
6b500b782f42c0d35daa56a0888d1c56	35	1.000	d__Bacteria	p__Acidobacteriota	c__Acidobacteriae	o__PAUC26f	f__PAUC26f	g__PAUC26f	s__uncultured_bacterium
85f72f312b6ced96a68e9cce2fdaf2c9	529	1.000	d__Bacteria	p__Acidobacteriota	c__Acidobacteriae	o__PAUC26f	f__PAUC26f	g__PAUC26f	s__uncultured_bacterium
f92ba592616a54b5cf690fef09b6c03f	184	1.000	d__Bacteria	p__Chloroflexi	c__TK30	o__TK30	f__TK30	g__TK30	s__uncultured_bacterium

1c9c1ed19c6d51c34 fb0ca057f53c4c6	31	1.000	d__Bacter ia	p__Chlorofl exi	c__Dehaloc occoidia	o__SAR202_ clade	f__SAR202 _clade	g__SAR202_cla de	
2eee99740ae0d7452 0aa4d63d886772b	9	1.000	d__Bacter ia	p__Chlorofl exi	c__Anaeroli neae	o__SBR1031	f__A4b	g__A4b	

Table S4. ASVs (148) from *Geodia cydonium* collected in Integrated Multi-Trophic Aquaculture (IMTA) system with percentage of confidence ≥ 75%.

ASVs ID	4	Confidence	Domain	Phylum	Class	Order	Family	Genus	Species
7bcb843aafd987aa71db4fea161b59c8	14	0.753	d__Bacteria	p__Chloroflexi	c__Dehalococcoidia	o__SAR202_clade	f__SAR202_clade	g__SAR202_clade	s__uncultured_bacterium
171683e9ab657be53420f66e81e776fb	2	0.761	d__Bacteria	p__Proteobacteria	c__Gammaproteobacteria	o__Pseudomonadales			
976054831b53b6e9fb17e154788a0294	1	0.768	d__Bacteria	p__Proteobacteria	c__Gammaproteobacteria	o__HOC36	f__HOC36	g__HOC36	
bb577d4d578ae8453d78433395a10a26	2	0.772	d__Bacteria	p__Bacteroidota	c__Rhodothermia	o__Rhodothermales	f__Rhodothermaceae	g__uncultured	s__uncultured_Bacteroidetes
203c6df9b41c402cd1c0c8b96533b9b4	7	0.780	d__Bacteria	p__Proteobacteria	c__Alphaproteobacteria	o__AT-s3-44	f__AT-s3-44	g__AT-s3-44	s__uncultured_bacterium
d4a6a0bdcd665720aaadddb1854c5a56	14	0.781	d__Bacteria	p__Poribacteria	c__Poribacteria	o__Poribacteria	f__Poribacteria	g__Poribacteria	s__Candidatus_Poribacteria
adc171c838c7078dff2da54b6540aa87	2	0.786	d__Bacteria	p__Chloroflexi	c__Dehalococcoidia	o__SAR202_clade	f__SAR202_clade	g__SAR202_clade	s__uncultured_bacterium
458b20ba69e5160ee718ba03913c6519	79	0.795	d__Bacteria	p__Gemmatimonadota	c__BD2-11_terrestrial_group	o__BD2-11_terrestrial_group	f__BD2-11_terrestrial_group	g__BD2-11_terrestrial_group	s__uncultured_bacterium
fdba3e43af94302a9b6a87163e792434	14	0.807	d__Bacteria	p__Poribacteria	c__Poribacteria	o__Poribacteria	f__Poribacteria	g__Poribacteria	s__Candidatus_Poribacteria
56ace4628efe08c52be2bc3b33b5dfde	367	0.814	d__Bacteria	p__Gemmatimonadota	c__BD2-11_terrestrial_group	o__BD2-11_terrestrial_group	f__BD2-11_terrestrial_group	g__BD2-11_terrestrial_group	s__uncultured_bacterium
798986fed73ab850f3cb13e8182c884c	117	0.814	d__Bacteria	p__Chloroflexi	c__Dehalococcoidia	o__SAR202_clade	f__SAR202_clade	g__SAR202_clade	s__uncultured_bacterium
063cce8658f9e598aa3a032f35ab3260	24	0.821	d__Bacteria	p__Chloroflexi	c__JG30-KF-CM66	o__JG30-KF-CM66	f__JG30-KF-CM66	g__JG30-KF-CM66	s__uncultured_bacterium

821d422ed366d6be46e0dd7e128db306	126	0.822	d__Bacteria	p__Actinobacteriota	c__Acidimicrobiia	o__Microtrichales	f__Microtrichaceae	g__Sva0996_marine_group	s__uncultured_bacterium
625d9075b54136eb6d844fb396c6f07b	3	0.822	d__Bacteria	p__Bacteroidota	c__Bacteroidia	o__Flavobacteriales	f__Flavobacteriaceae	g__NS5_marine_group	s__uncultured_Flavobacterium
0d234efa5aabd14c58a506f8748c6b02	18	0.828	d__Bacteria	p__Gemmatimonadota	c__BD2-11_terrestrial_group	o__BD2-11_terrestrial_group	f__BD2-11_terrestrial_group	g__BD2-11_terrestrial_group	s__uncultured_delta
cc5d3b00b10d0a4ac2fffbd36ec549bd	2	0.849	d__Bacteria	p__Proteobacteria	c__Alphaproteobacteria	o__Defluviicoccales	f__uncultured	g__uncultured	s__uncultured_bacterium
e5feb5f19a7440606cf8a57bf0d77f19	5	0.852	d__Bacteria	p__Chloroflexi	c__Anaerolineae	o__Caldilineales	f__Caldilineaceae	g__uncultured	s__uncultured_Caldilinea
d372497992884eb99e5ddd090a5aa470	4	0.854	d__Bacteria	p__Proteobacteria	c__Gammaproteobacteria	o__JTB23	f__JTB23	g__JTB23	s__uncultured_proteobacterium
90b1bd01a549457d8cbbf557b46cf32e	1	0.859	d__Bacteria	p__Myxococcota	c__bacteriap25	o__bacteriap25	f__bacteriap25	g__bacteriap25	s__uncultured_bacterium
a4a2b3ac9ec1333d2c8b5c90e9717ac6	21	0.864	d__Bacteria	p__Chloroflexi	c__Dehalococcoidia	o__SAR202_clade	f__SAR202_clade	g__SAR202_clade	s__uncultured_bacterium
effa34f80a6e5c8e6504ed2cf928ac3b	98	0.866	d__Bacteria	p__Actinobacteriota	c__Acidimicrobiia	o__Actinomarinales	f__uncultured	g__uncultured	s__uncultured_actinobacterium
ebeb45524dd399fc558fcb9d836e176f	4	0.866	d__Bacteria	p__Poribacteria	c__Poribacteria	o__Poribacteria	f__Poribacteria	g__Poribacteria	s__uncultured_Poribacteria
3556f90a547bfed0cabe2d6eaf75691b	5	0.872	d__Bacteria	p__Acidobacteriota	c__Vicinamibacteria	o__Vicinamibacterales	f__uncultured	g__uncultured	s__uncultured_bacterium
a2bd0c7a17499882cf2b4002d4c302e6	48	0.874	d__Bacteria	p__Chloroflexi	c__JG30-KF-CM66	o__JG30-KF-CM66	f__JG30-KF-CM66	g__JG30-KF-CM66	s__uncultured_Chloroflexi
fd17b3b166715bdb837bcbaaeefcd2c3	35	0.884	d__Bacteria	p__Chloroflexi	c__JG30-KF-CM66	o__JG30-KF-CM66	f__JG30-KF-CM66	g__JG30-KF-CM66	s__uncultured_Chloroflexi
9d8d12932083466e65c9214e93a11e74	15	0.887	d__Bacteria	p__Proteobacteria	c__Alphaproteobacteria	o__Rhodobacterales	f__Rhodobacteraceae	g__uncultured	

97fb8ec1addf7f02c1b8ff7f49627563	100	0.888	d__Bacteria	p__Proteobacteria	c__Gammaproteobacteria	o__HOC36	f__HOC36	g__HOC36	s__uncultured_gamma
88b6d081caec18a35df2037295886130	51	0.891	d__Bacteria	p__Chloroflexi	c__Anaerolineae	o__SBR1031	f__A4b	g__A4b	s__uncultured_Chloroflexus
8615e35486878ed31ea0bad428435fbd	81	0.899	d__Bacteria	p__Chloroflexi	c__Anaerolineae	o__SBR1031	f__A4b	g__A4b	s__uncultured_Chloroflexus
05570da352ff8bd242da1548e1db25fc	21	0.900	d__Bacteria	p__Chloroflexi	c__Dehalococcoidia	o__SAR202_clade	f__SAR202_clade	g__SAR202_clade	s__uncultured_bacterium
0afe9f04fb31196f59b6f0853098bcff	91	0.907	d__Bacteria	p__Actinobacteriota	c__Acidimicrobiia	o__Microtrichales	f__Microtrichaceae	g__Sva0996_marine_group	s__uncultured_bacterium
cf8c413052193af977287d9da18c4d39	92	0.909	d__Bacteria	p__Chloroflexi	c__Anaerolineae	o__SBR1031	f__A4b	g__A4b	s__uncultured_Chloroflexus
c4f5adcf981a60fe5fd13da1f679b923	31	0.928	d__Bacteria	p__Acidobacteriota	c__Vicinamibacteria	o__Vicinamibacterales	f__uncultured	g__uncultured	s__uncultured_bacterium
7002c643193e83563c91d1e6ffc44302	72	0.928	d__Bacteria	p__Gemmatimonadota	c__BD2-11_terrestrial_group	o__BD2-11_terrestrial_group	f__BD2-11_terrestrial_group	g__BD2-11_terrestrial_group	s__uncultured_bacterium
cba9d660a3b3f51516ac7553dfee4294	8	0.931	d__Bacteria	p__Gemmatimonadota	c__BD2-11_terrestrial_group	o__BD2-11_terrestrial_group	f__BD2-11_terrestrial_group	g__BD2-11_terrestrial_group	s__uncultured_bacterium
d737fcb1d338aadb5780a96821ac30b5	52	0.933	d__Bacteria	p__Proteobacteria	c__Alphaproteobacteria	o__Defluviicoccales	f__uncultured	g__uncultured	s__uncultured_bacterium
e4fc3333123c849329d7675c66df815a	59	0.934	d__Bacteria	p__Proteobacteria	c__Alphaproteobacteria	o__Defluviicoccales	f__uncultured	g__uncultured	s__uncultured_bacterium
a97eae53c423c75d5bf421e68fd34ccd	132	0.938	d__Bacteria	p__Chloroflexi	c__Dehalococcoidia	o__SAR202_clade	f__SAR202_clade	g__SAR202_clade	s__uncultured_SAR202
89dbf04c406407551ef8892caa15a009	37	0.942	d__Bacteria	p__Proteobacteria	c__Alphaproteobacteria	o__Kiloniellales	f__Kiloniellaceae	g__uncultured	s__uncultured_bacterium
329ae64e4773b4b09ea1512613fce42d	4	0.946	d__Bacteria	p__Chloroflexi	c__Dehalococcoidia	o__SAR202_clade	f__SAR202_clade	g__SAR202_clade	

a7a13906d768ed91d2fdbd5b7e4a6b5a	3	0.954	d__Bacteria	p__Proteobacteria	c__Gammaproteobacteria	o__Pseudomonadales	f__KI89A_clade	g__KI89A_clade	s__uncultured_organism
c22e791a1ed82997aa6d9435da8fc4c8	8	0.954	d__Archaea	p__Crenarchaeota	c__Nitrososphaeria	o__Nitrosopumilales	f__Nitrosopumilaceae	g__Candidatus_Nitrosopumilus	s__uncultured_archaeon
bde571c1071072dab43ee92c07079ca7	5	0.955	d__Bacteria	p__Gemmatimonadota	c__BD2-11_terrestrial_group	o__BD2-11_terrestrial_group	f__BD2-11_terrestrial_group	g__BD2-11_terrestrial_group	s__uncultured_bacterium
0a5711cb90b810d51cf6a5167df29c40	11	0.956	d__Bacteria	p__Proteobacteria	c__Gammaproteobacteria	o__UBA10353_marine_group	f__UBA10353_marine_group	g__UBA10353_marine_group	s__uncultured_bacterium
4c31d6515474234ca32b8c4ba923f87a	23	0.957	d__Bacteria	p__Acidobacteriota	c__Vicinamibacteria	o__Vicinamibacterales	f__uncultured	g__uncultured	s__uncultured_bacterium
f9404661ca724985ec4de10ad921336f	122	0.959	d__Bacteria	p__Proteobacteria	c__Gammaproteobacteria	o__EPR3968-O8a-Bc78	f__EPR3968-O8a-Bc78	g__EPR3968-O8a-Bc78	s__uncultured_bacterium
57f503246c1f0a094b5042b7ac19ba17	81	0.960	d__Bacteria	p__Actinobacteriota	c__Acidimicrobiia	o__Microtrichales	f__Microtrichaceae	g__Sva0996_marine_group	s__uncultured_bacterium
1c94a21bcf883f1ebbc7a7898707aa18	2	0.961	d__Bacteria	p__Dadabacteria	c__Dadabacteriia	o__Dadabacteriales	f__Dadabacteriales	g__Dadabacteriales	s__uncultured_delta
1f4fe56a542b451272bfffbfc4447b93	19	0.961	d__Bacteria	p__Nitrospinota	c__P9X2b3D02	o__P9X2b3D02	f__P9X2b3D02	g__P9X2b3D02	s__uncultured_bacterium
eb8e136acd9343a1e960022c06dfb5e4	43	0.962	d__Bacteria	p__Proteobacteria	c__Gammaproteobacteria	o__Pseudomonadales	f__Halieaceae		
292296a6d4dd77a844c8d1dbd5955141	2	0.964	d__Bacteria	p__Gemmatimonadota	c__BD2-11_terrestrial_group	o__BD2-11_terrestrial_group	f__BD2-11_terrestrial_group	g__BD2-11_terrestrial_group	s__uncultured_bacterium
7bc1a7ab05aaa1be8999f4f56fc0d5e9	12	0.965	d__Bacteria	p__Entotheonellaeota	c__Entotheonellia	o__Entotheonellales	f__Entotheonellaceae	g__Entotheonellaceae	s__uncultured_delta
40c2b630e6829314f805e1d934951f02	566	0.972	d__Bacteria	p__Bacteroidota	c__Rhodothermia	o__Rhodothermales	f__Rhodothermaceae	g__uncultured	s__uncultured_bacterium
aaaafcfa8ae466a0ee7f15836747b06e	4	0.972	d__Bacteria	p__Proteobacteria	c__Gammaproteobacteria	o__EPR3968-O8a-Bc78	f__EPR3968-O8a-Bc78	g__EPR3968-O8a-Bc78	s__uncultured_bacterium

c1bc5c783371c11161d56cf7b40e3828	147	0.972	d__Bacteria	p__Proteobacteria	c__Gammaproteobacteria	o__Pseudomonadales	f__Pseudohongiellaceae	g__Pseudohongiella	s__uncultured_bacterium
1fca9214ea64502cf023fa6372a8498d	47	0.976	d__Bacteria	p__Proteobacteria	c__Alphaproteobacteria	o__Kiloniellales	f__Kiloniellaceae	g__uncultured	s__uncultured_bacterium
bf7c48db5b7f62245f4e6507542ae90d	80	0.976	d__Bacteria	p__Actinobacteriota	c__Acidimicrobiia	o__Microtrichales	f__Microtrichaceae	g__Sva0996_marine_group	s__uncultured_bacterium
5ae1f84dfcc9b445055b21815e530c4b	2	0.977	d__Bacteria	p__Bacteroidota	c__Bacteroidia	o__Flavobacteriales	f__Flavobacteriaceae	g__NS5_marine_group	
80447419c3a77efab5e7e756afc64bf0	2	0.978	d__Bacteria	p__Chloroflexi	c__Dehalococcoidia	o__SAR202_clade	f__SAR202_clade	g__SAR202_clade	s__uncultured_bacterium
d2cfdab5b2e6abb83dfe0df87e7d5606	38	0.979	d__Bacteria	p__Chloroflexi	c__Anaerolineae	o__SBR1031	f__A4b	g__A4b	s__uncultured_Chloroflexi
9ac1d376f84dff0b4a0b11b609620bfa	6	0.979	d__Bacteria	p__Proteobacteria	c__Alphaproteobacteria	o__Rhodobacterales	f__Rhodobacteraceae	g__Ascidiaceihabitans	s__uncultured_bacterium
092bc671bc1d2c9f2318e9f5f22a2ea1	21	0.979	d__Bacteria	p__Myxococcota	c__bacteriap25	o__bacteriap25	f__bacteriap25	g__bacteriap25	s__uncultured_delta
318c3c6a14aed9d629284ae6c933bc18	744	0.980	d__Bacteria	p__Actinobacteriota	c__Acidimicrobiia	o__Microtrichales	f__Microtrichaceae	g__Sva0996_marine_group	s__uncultured_bacterium
e68d370d47f1c36c771646ca1ad728ff	59	0.980	d__Bacteria	p__Actinobacteriota	c__Acidimicrobiia	o__Microtrichales	f__Microtrichaceae	g__Sva0996_marine_group	s__uncultured_actinobacterium
97fe1f4321eafb369840e509d6e7a85a	196	0.983	d__Bacteria	p__Actinobacteriota	c__Acidimicrobiia	o__Actinomarinales	f__uncultured	g__uncultured	s__uncultured_actinobacterium
1348e101213478d6255d9ac1ff209150	2	0.985	d__Bacteria	p__Myxococcota	c__bacteriap25	o__bacteriap25	f__bacteriap25	g__bacteriap25	s__uncultured_bacterium
25089632a779c4849f36e514b6f74631	40	0.986	d__Bacteria	p__Chloroflexi	c__Dehalococcoidia	o__SAR202_clade	f__SAR202_clade	g__SAR202_clade	s__uncultured_bacterium
0b634770abf69b42997028d717039878	2	0.986	d__Bacteria	p__Chloroflexi	c__Dehalococcoidia	o__SAR202_clade	f__SAR202_clade	g__SAR202_clade	s__uncultured_bacterium

6ce359edd2145bb37b0fe5100c5682b3	312	0.986	d__Bacteria	p__Proteobacteria	c__Gammaproteobacteria	o__EPR3968-O8a-Bc78	f__EPR3968-O8a-Bc78	g__EPR3968-O8a-Bc78	s__uncultured_bacterium
a5f252c77ba9ec05e3210792c7ad8a69	36	0.989	d__Bacteria	p__Proteobacteria	c__Gammaproteobacteria	o__EPR3968-O8a-Bc78	f__EPR3968-O8a-Bc78	g__EPR3968-O8a-Bc78	s__uncultured_bacterium
07ba1daf9dabbb42b72f88e08c6ae33c	14	0.989	d__Bacteria	p__Myxococcota	c__bacteriap25	o__bacteriap25	f__bacteriap25	g__bacteriap25	s__uncultured_delta
546627ba3f2471e8f05ccfdb26c29d68	30	0.990	d__Bacteria	p__AncK6	c__AncK6	o__AncK6	f__AncK6	g__AncK6	s__uncultured_bacterium
bd02a34a26b7aeb88f5f22841501a945	56	0.991	d__Bacteria	p__Chloroflexi	c__Dehalococcoidia	o__SAR202_clade	f__SAR202_clade	g__SAR202_clade	s__uncultured_bacterium
955fea05d55918f3682e897ba8137e23	247	0.993	d__Bacteria	p__Chloroflexi	c__Anaerolineae	o__Caldilineales	f__Caldilineaceae	g__uncultured	s__uncultured_Caldilinea
0d864eef2bc2c0e980088ad43d873c36	161	0.994	d__Bacteria	p__Actinobacteriota	c__Acidimicrobiia	o__Microtrichales	f__Microtrichaceae	g__Sva0996_marine_group	s__uncultured_actinobacterium
0bce9e193dd20323ff34751f8711d95e	136	0.994	d__Bacteria	p__Poribacteria	c__Poribacteria	o__Poribacteria	f__Poribacteria	g__Poribacteria	s__uncultured_Poribacteria
b335c839d0eeb10db2b0c527a8015a4d	23	0.994	d__Bacteria	p__Proteobacteria	c__Gammaproteobacteria	o__pItb-vmat-80	f__pItb-vmat-80	g__pItb-vmat-80	s__uncultured_bacterium
b11e746055b3692569dac165ecf828e9	305	0.994	d__Bacteria	p__Actinobacteriota	c__Acidimicrobiia	o__Microtrichales	f__Microtrichaceae	g__Sva0996_marine_group	s__uncultured_bacterium
f74bf88be3c3fc3aa089270adfe9964d	83	0.995	d__Bacteria	p__Proteobacteria	c__Gammaproteobacteria	o__JTB23	f__JTB23	g__JTB23	s__uncultured_proteobacterium
dd4ec1bdb423917dc44d526d75dc08ed	46	0.995	d__Bacteria	p__Chloroflexi	c__Dehalococcoidia	o__SAR202_clade	f__SAR202_clade	g__SAR202_clade	s__uncultured_bacterium
f8c2578000e8d8783bdf16642fb4c705	24	0.997	d__Bacteria	p__Acidobacteriota	c__Subgroup_21	o__Subgroup_21	f__Subgroup_21	g__Subgroup_21	s__uncultured_bacterium
837089e5175b2b181227d6fac488da3d	10	0.998	d__Bacteria	p__Proteobacteria	c__Alphaproteobacteria	o__Puniceispirillales	f__EF100-94H03	g__EF100-94H03	s__uncultured_bacterium

6c809937e62b7b9e3d9680dde8514458	6	0.998	d__Bacteria	p__Proteobacteria	c__Gammaproteobacteria	o__pItb-vmat-80	f__pItb-vmat-80	g__pItb-vmat-80	s__uncultured_bacterium
052ace1004921da16db003cbafc2cfee	6	0.998	d__Bacteria	p__Proteobacteria	c__Gammaproteobacteria	o__UBA10353_marine_group	f__UBA10353_marine_group	g__UBA10353_marine_group	s__uncultured_marine
af10a20a19fb941c5021ee854d0b68e3	169	0.998	d__Bacteria	p__Proteobacteria	c__Gammaproteobacteria	o__JTB23	f__JTB23	g__JTB23	
39ad9d5e1d861b8925bffb1082e586f5	100	0.998	d__Bacteria	p__Acidobacteriota	c__Vicinamibacteria	o__Subgroup_9	f__Subgroup_9	g__Subgroup_9	s__uncultured_bacterium
cf3b42c97010fcf8952173c83b20f8c4	28	0.999	d__Bacteria	p__Proteobacteria	c__Gammaproteobacteria	o__JTB23	f__JTB23	g__JTB23	
014e17b29a1c01cae4ff773cca164a1c	297	0.999	d__Bacteria	p__Actinobacteriota	c__Acidimicrobiia	o__Microtrichales	f__Microtrichaceae	g__Sva0996_marine_group	
f5d701080efc2363c77a0993a8177ae2	2	0.999	d__Bacteria	p__Chloroflexi	c__TK17	o__TK17	f__TK17	g__TK17	s__uncultured_Chloroflexi
1f5b9d32f3040bcb7d88190d889f79de	15	0.999	d__Bacteria	p__Chloroflexi	c__Dehalococcoidia	o__SAR202_clade	f__SAR202_clade	g__SAR202_clade	s__uncultured_bacterium
236c68919f45214405ad01681fd3f6bb	53	0.999	d__Bacteria	p__Proteobacteria	c__Gammaproteobacteria	o__Nitrosococcales	f__Nitrosococcaceae	g__AqS1	s__uncultured_bacterium
b5b0f076cba7d03c4f90d32eec106ab6	14	0.999	d__Bacteria	p__Gemmatimonadota	c__PAUC43f_marine_benthic_group	o__PAUC43f_marine_benthic_group	f__PAUC43f_marine_benthic_group	g__PAUC43f_marine_benthic_group	s__uncultured_bacterium
197113967fc06328d4ab3b548355fc05	19	0.999	d__Bacteria	p__Chloroflexi	c__Dehalococcoidia	o__SAR202_clade	f__SAR202_clade	g__SAR202_clade	
f72871b2770cbfacc1d095d45ff7b3e4	3032	0.999	d__Bacteria	p__Actinobacteriota	c__Acidimicrobiia	o__Microtrichales	f__Microtrichaceae	g__Sva0996_marine_group	
e2c26552aeb18406f3330992b07b31a7	28	0.999	d__Bacteria	p__Acidobacteriota	c__Vicinamibacteria	o__Subgroup_9	f__Subgroup_9	g__Subgroup_9	s__uncultured_bacterium

b936c98dae871c62c562ca5d09bb1809	10	0.999	d__Bacteria	p__Proteobacteria	c__Alphaproteobacteria	o__Rhodospirillales	f__Magnetospiraceae	g__uncultured	s__uncultured_bacterium
d77f6bd395b7c11f9ba2aa8720131dc9	100	0.999	d__Bacteria	p__Myxococcota	c__bacteriap25	o__bacteriap25	f__bacteriap25	g__bacteriap25	s__uncultured_bacterium
85383c420d3feb0185f0a45a5bb830d2	18	0.999	d__Bacteria	p__Poribacteria	c__Poribacteria	o__Poribacteria	f__Poribacteria	g__Poribacteria	s__Candidatus_Poribacteria
2fb370908d414cb24f17d51fe45aa3ac	5	0.999	d__Bacteria	p__Chloroflexi	c__Anaerolineae	o__Caldilineales	f__Caldilineaceae	g__uncultured	s__uncultured_Chloroflexus
8995f47f6b5c70dad28854d6066cfd50	43	0.999	d__Bacteria	p__Proteobacteria	c__Gammaproteobacteria	o__Pseudomonadales	f__KI89A_clade	g__KI89A_clade	s__uncultured_bacterium
c3a426b285ac1d04ef66f01d59891e1f	81	0.999	d__Bacteria	p__Acidobacteriota	c__Acidobacteriae	o__PAUC26f	f__PAUC26f	g__PAUC26f	s__uncultured_bacterium
aa3d9a6455de34012e8b0a8bd268b556	104	1.000	d__Bacteria	p__Proteobacteria	c__Alphaproteobacteria	o__Defluviicoccales	f__uncultured	g__uncultured	s__uncultured_bacterium
a087e2859a5bca997c5d6595c0ac3280	18	1.000	d__Bacteria	p__Cyanobacteria	c__Cyanobacteriia	o__Synechococcales	f__Cyanobiaceae	g__Synechococcus_CC9902	
f1388a44b9b1abce510aaf7c1e238e2e	4	1.000	d__Bacteria	p__PAUC34f	c__PAUC34f	o__PAUC34f	f__PAUC34f	g__PAUC34f	s__uncultured_bacterium
eb5b65811133836c930139020175f1ee	39	1.000	d__Bacteria	p__Chloroflexi	c__Dehalococcoidia	o__SAR202_clade	f__SAR202_clade	g__SAR202_clade	s__uncultured_deep-sea
a7febd04d2c878e674539ac24d0c5c03	45	1.000	d__Bacteria	p__Chloroflexi	c__Dehalococcoidia	o__SAR202_clade	f__SAR202_clade	g__SAR202_clade	s__uncultured_deep-sea
e9137e128559135e1a3cf6b20f59059b	175	1.000	d__Bacteria	p__Proteobacteria	c__Gammaproteobacteria	o__Pseudomonadales	f__KI89A_clade	g__KI89A_clade	s__uncultured_bacterium
ab6151de482049167acb71a2969446f5	409	1.000	d__Bacteria	p__Proteobacteria	c__Alphaproteobacteria	o__Defluviicoccales	f__uncultured	g__uncultured	s__uncultured_bacterium
308b149b607d495174541fb08ba2c10c	58	1.000	d__Bacteria	p__Proteobacteria	c__Gammaproteobacteria	o__Steroidobacterales	f__Woeseiaceae	g__JTB255_marine_benthic_group	s__uncultured_bacterium

90ea6258ef90afe11ec8675f4356296d	45	1.000	d__Bacteria	p__Proteobacteria	c__Gammaproteobacteria	o__Steroidobacterales	f__Woeseiaceae	g__JTB255_marine_benthic_group	s__uncultured_bacterium
67d32d9ad1b8d9919894a6b554d0742f	31	1.000	d__Bacteria	p__Acidobacteriota	c__Thermoanaerobaculia	o__Thermoanaerobaculales	f__Thermoanaerobaculaceae	g__Subgroup_10	s__uncultured_bacterium
04ead0e7f6466e35395823079cef35f4	201	1.000	d__Bacteria	p__Proteobacteria	c__Gammaproteobacteria	o__Steroidobacterales	f__Woeseiaceae	g__JTB255_marine_benthic_group	s__uncultured_bacterium
f829766bfb6ed4c8e50e44ad3fd7d070	190	1.000	d__Bacteria	p__Nitrospirota	c__Nitrospiria	o__Nitrospirales	f__Nitrospiraceae	g__Nitrospira	s__uncultured_bacterium
5a8c1c9db3c3545353c94d4f478ef15d	435	1.000	d__Bacteria	p__Dadabacteria	c__Dadabacteriia	o__Dadabacteriales	f__Dadabacteriales	g__Dadabacteriales	s__uncultured_delta
0d8f666c087c9f2d3f828cf2f2316539	387	1.000	d__Bacteria	p__Proteobacteria	c__Alphaproteobacteria	o__Puniceispirillales	f__Puniceispirillales_Incertae_Sedis	g__Constrictibacter	s__uncultured_bacterium
aa0bb46f8879fdd1efd50ce3d7859c75	31	1.000	d__Bacteria	p__Proteobacteria	c__Gammaproteobacteria	o__Pseudomonadales	f__KI89A_clade	g__KI89A_clade	s__uncultured_bacterium
72d2ebaa8ad88e767f268b15feaeb3ff	19	1.000	d__Bacteria	p__Proteobacteria	c__Gammaproteobacteria	o__Nitrosococcales	f__Nitrosococcaceae	g__AqS1	s__uncultured_bacterium
0e273566d4831ef207b700781fbe28f1	2	1.000	d__Bacteria	p__PAUC34f	c__PAUC34f	o__PAUC34f	f__PAUC34f	g__PAUC34f	s__uncultured_bacterium
b96b1e589e704b6a643f86890117cfa7	520	1.000	d__Bacteria	p__Nitrospirota	c__Nitrospiria	o__Nitrospirales	f__Nitrospiraceae	g__Nitrospira	s__uncultured_bacterium
35b4dd5114b178277529a8aab246c67a	29	1.000	d__Bacteria	p__PAUC34f	c__PAUC34f	o__PAUC34f	f__PAUC34f	g__PAUC34f	s__uncultured_bacterium
b959053936fe72856bac9724c29645b3	2158	1.000	d__Bacteria	p__Chloroflexi	c__Anaerolineae	o__Caldilineales	f__Caldilineaceae	g__uncultured	s__uncultured_Chloroflexus
d6a79416c0b360cb1c7da2b29c779121	49	1.000	d__Bacteria	p__Verrucomicrobiota	c__Verrucomicrobiae	o__Opitutales	f__Puniceicoccaceae	g__Cerasicoccus	s__uncultured_Verrucomicrobia
e1731bd5d1e591d09317ee220ce0d3e7	59	1.000	d__Bacteria	p__Chloroflexi	c__Dehalococcoidia	o__SAR202_clade	f__SAR202_clade	g__SAR202_clade	

13a39791e7af03689920c11baf639f3f	4	1.000	d__Bacteria	p__Chloroflexi	c__TK17	o__TK17	f__TK17	g__TK17	s__uncultured_Chloroflexi
fc874f067345bcf51a64fcd59a91f979	8	1.000	d__Bacteria	p__Spirochaetota	c__Spirochaetia	o__Spirochaetales	f__Spirochaetaceae	g__Spirochaeta	s__uncultured_bacterium
876e2f7e37b7db45be54a9befdb1a363	29	1.000	d__Bacteria	p__Poribacteria	c__Poribacteria	o__Poribacteria	f__Poribacteria	g__Poribacteria	s__uncultured_Poribacteria
f5b28b630059b6f704b3926f9ac71356	34	1.000	d__Bacteria	p__Proteobacteria	c__Gammaproteobacteria	o__Steroidobacterales	f__Woeseiaceae	g__JTB255_marine_benthic_group	s__uncultured_bacterium
315ca649e9266db51e286539b0cc4318	11	1.000	d__Bacteria	p__Chloroflexi	c__Anaerolineae	o__Caldilineales	f__Caldilineaceae	g__uncultured	s__uncultured_Chloroflexus
8e2b88fa6680528734b9570ffd855be8	2228	1.000	d__Bacteria	p__Chloroflexi	c__Anaerolineae	o__Caldilineales	f__Caldilineaceae	g__uncultured	s__uncultured_Chloroflexus
712869e9bf8bcbe9e2e4d535a1e3db0b	98	1.000	d__Bacteria	p__Proteobacteria	c__Gammaproteobacteria	o__Pseudomonadales	f__KI89A_clade	g__KI89A_clade	
e43520bae8985c61c312f7daa2da201a	5	1.000	d__Bacteria	p__Chloroflexi	c__Dehalococcoidia	o__SAR202_clade	f__SAR202_clade	g__SAR202_clade	
7be2632751b6fce52f846e031c9b99b4	88	1.000	d__Bacteria	p__Acidobacteriota	c__Acidobacteriae	o__PAUC26f	f__PAUC26f	g__PAUC26f	s__uncultured_bacterium
863382f82e439c69fba5f72df01794b8	83	1.000	d__Bacteria	p__Acidobacteriota	c__Vicinamibacteria	o__Vicinamibacterales	f__uncultured	g__uncultured	
964bec32fd04991db6e033573c4667ac	152	1.000	d__Bacteria	p__Chloroflexi	c__TK17	o__TK17	f__TK17	g__TK17	s__uncultured_Chloroflexi
89674a06a0c850641379a5bb3f7de0e9	13	1.000	d__Bacteria	p__Chloroflexi	c__Dehalococcoidia	o__SAR202_clade	f__SAR202_clade	g__SAR202_clade	
ab46399cc898e26437ea05f7fc39510f	343	1.000	d__Bacteria	p__Chloroflexi	c__TK17	o__TK17	f__TK17	g__TK17	s__uncultured_Chloroflexi
59f066cbee429608952707821137649e	50	1.000	d__Bacteria	p__Proteobacteria	c__Gammaproteobacteria	o__Pseudomonadales	f__KI89A_clade	g__KI89A_clade	

85f72f312b6ced96a68e9cce2fdaf2c9	1062	1.000	d__Bacteria	p__Acidobacteriota	c__Acidobacteriae	o__PAUC26f	f__PAUC26f	g__PAUC26f	s__uncultured_bacterium
843112c78dc623e4a22e6445bb28f738	261	1.000	d__Bacteria	p__Chloroflexi	c__Dehalococcoidia	o__SAR202_clade	f__SAR202_clade	g__SAR202_clade	
f92ba592616a54b5cf690fef09b6c03f	23	1.000	d__Bacteria	p__Chloroflexi	c__TK30	o__TK30	f__TK30	g__TK30	s__uncultured_bacterium
f666a59c136d4c02014b23acca2cd2a7	79	1.000	d__Bacteria	p__Chloroflexi	c__TK30	o__TK30	f__TK30	g__TK30	s__uncultured_bacterium
2d77290bdffb556285d2fc8ee564ecb0	202	1.000	d__Bacteria	p__Proteobacteria	c__Gammaproteobacteria	o__Pseudomonadales	f__OM182_clade	g__OM182_clade	
1c9c1ed19c6d51c34fb0ca057f53c4c6	333	1.000	d__Bacteria	p__Chloroflexi	c__Dehalococcoidia	o__SAR202_clade	f__SAR202_clade	g__SAR202_clade	
2eee99740ae0d74520aa4d63d886772b	6	1.000	d__Bacteria	p__Chloroflexi	c__Anaerolineae	o__SBR1031	f__A4b	g__A4b	
374255d4f186035aeeab8644d17324ac	372	1.000	d__Bacteria	p__Poribacteria	c__Poribacteria	o__Poribacteria	f__Poribacteria	g__Poribacteria	s__Candidatus_Poribacteria
5a2e587ac3fc8064fe70bb3592c72f69	46	1.000	d__Bacteria	p__Acidobacteriota	c__Subgroup_11	o__Subgroup_11	f__Subgroup_11	g__Subgroup_11	s__uncultured_bacterium
5af73ce6f1540dcb0ad2ca4b79de56e4	4	1.000	d__Bacteria	p__Gemmatimonadota	c__BD2-11_terrestrial_group	o__BD2-11_terrestrial_group	f__BD2-11_terrestrial_group	g__BD2-11_terrestrial_group	
a3666add35726c9ee275b0e5aa7465f9	89	1.000	d__Bacteria	p__Chloroflexi	c__Anaerolineae	o__SBR1031	f__A4b	g__A4b	

Figure S1. Krona plot representing the most abundant class for each sample of *G. cydonium* under analysis: A) Gcyd_MP (Mar Piccolo), B) Gcyd_N (Gulf of Naples), C) Gcyd_P (Polignano a Mare), D) Gcyd_A (IMTA system).

A)

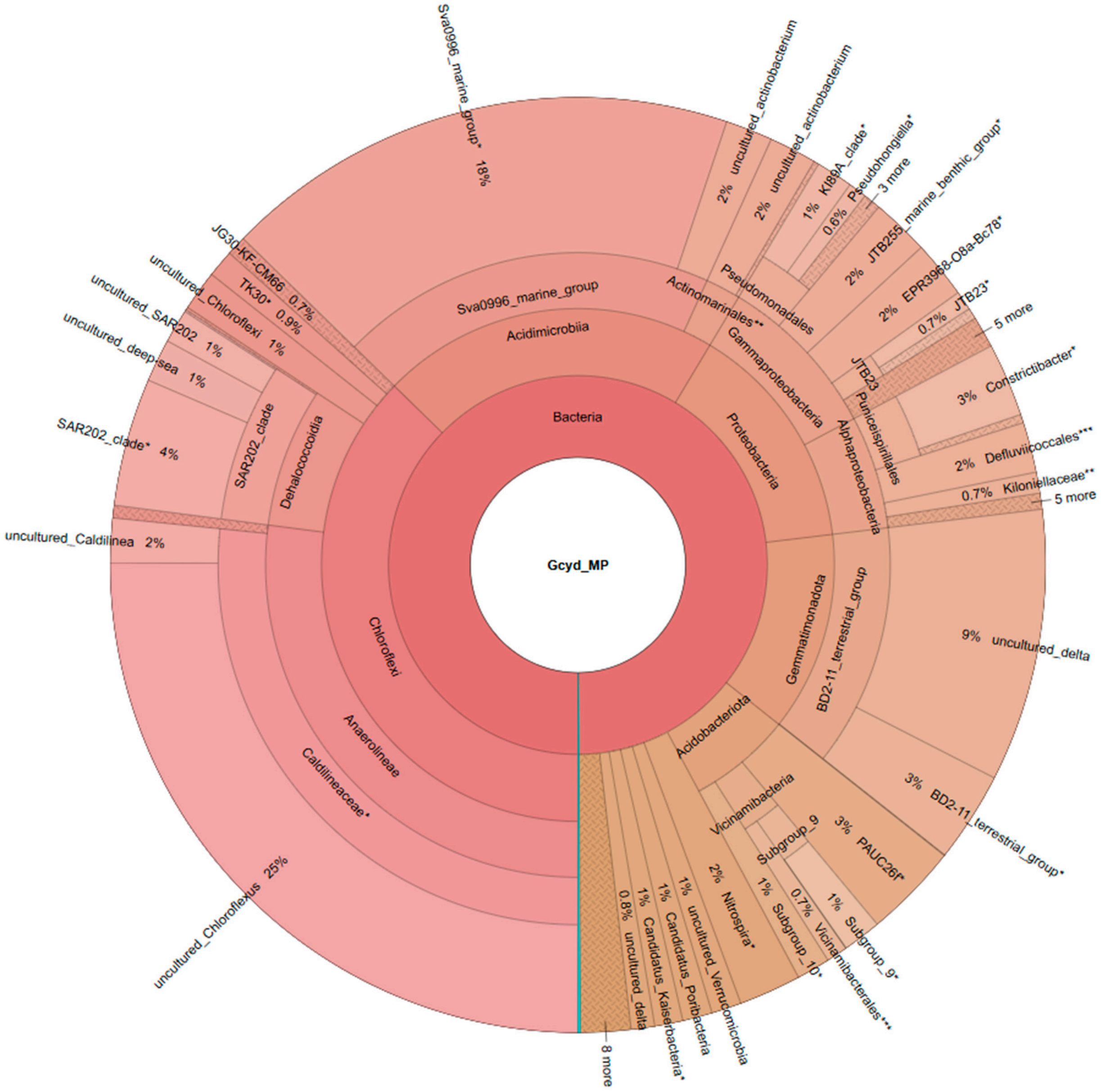

B)

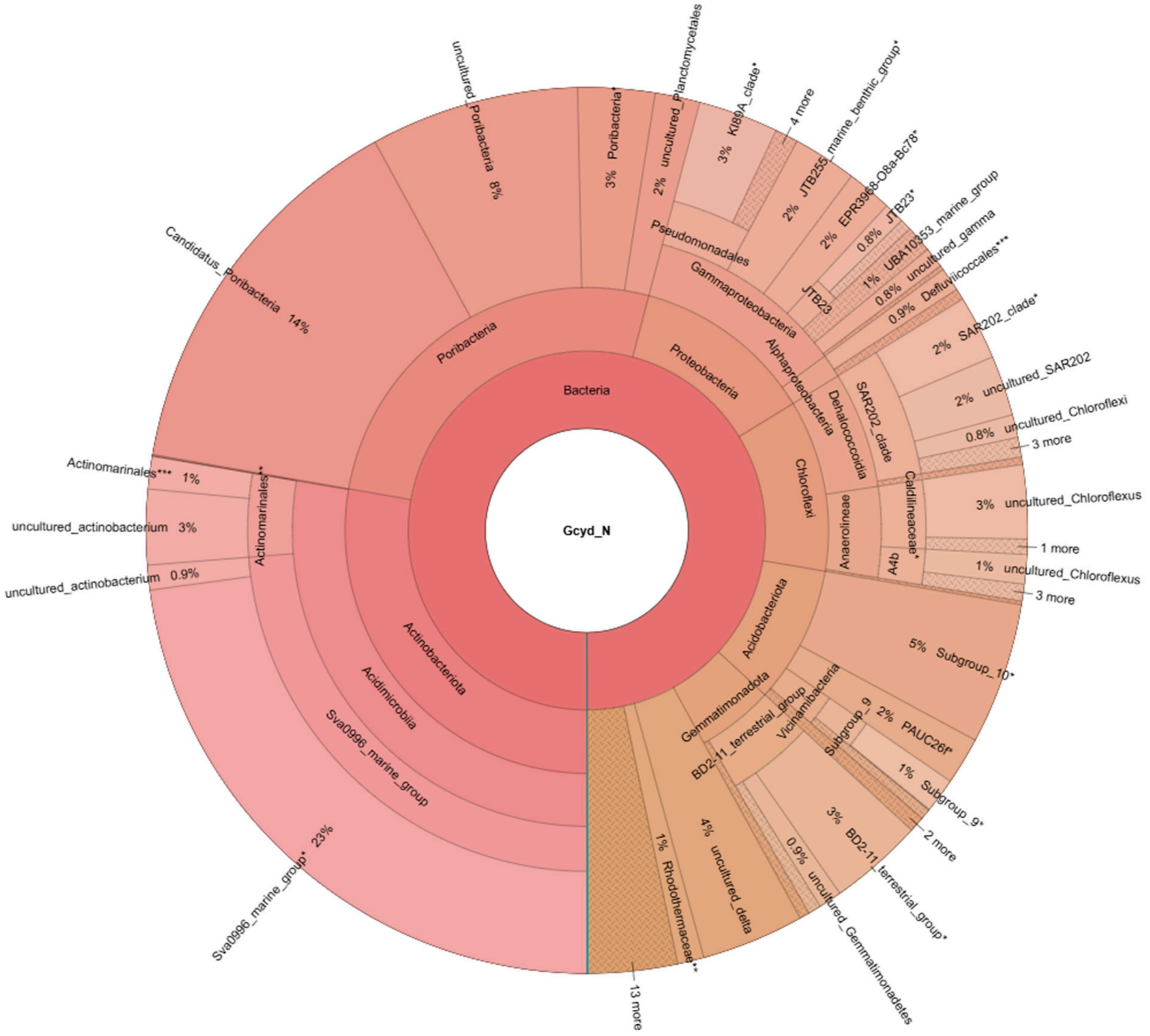

Gcyd_N
Bacteria
Poribacteria
Candidatus_Poribacteria 14%
uncultured_Poribacteria 8%
3% Poribacteria*
2% uncultured_planctomycetales
3% Kl89A_clade*
4 more
Proteobacteria
Gammaproteobacteria
Pseudomonadales
2% JTB255_marine_benthic_group*
2% EPR3968-O8a-Bc78*
JTB23
0.8% JTB23*
1% UBA10353_marine_group
0.8% uncultured_gamma
0.9% Defluviicoccales***
Alphaproteobacteria
Chloroflexi
Dehalococcoidia
SAR202_clade
2% SAR202_clade*
2% uncultured_SAR202
0.8% uncultured_Chloroflexi
3 more
Anaerolineae
Caldilineaceae*
A4b
3% uncultured_Chloroflexus
1 more
1% uncultured_Chloroflexus
3 more
Acidobacteriota
5% Subgroup_10*
Vicinamibacteria
Subgroup_9
2% PAUC26f*
1% Subgroup_9*
2 more
Gemmatimonadota
BD2-11_terrestrial_group
3% BD2-11_terrestrial_group*
0.9% uncultured_Gemmatimonadetes
4% uncultured_delta
1% Rhodothermaceae**
13 more
Actinobacteriota
Acidimicrobiia
Sva0996_marine_group
Sva0996_marine_group* 23%
Actinomarinales**
Actinomarinales*** 1%
uncultured_actinobacterium 3%
uncultured_actinobacterium 0.9%

C)

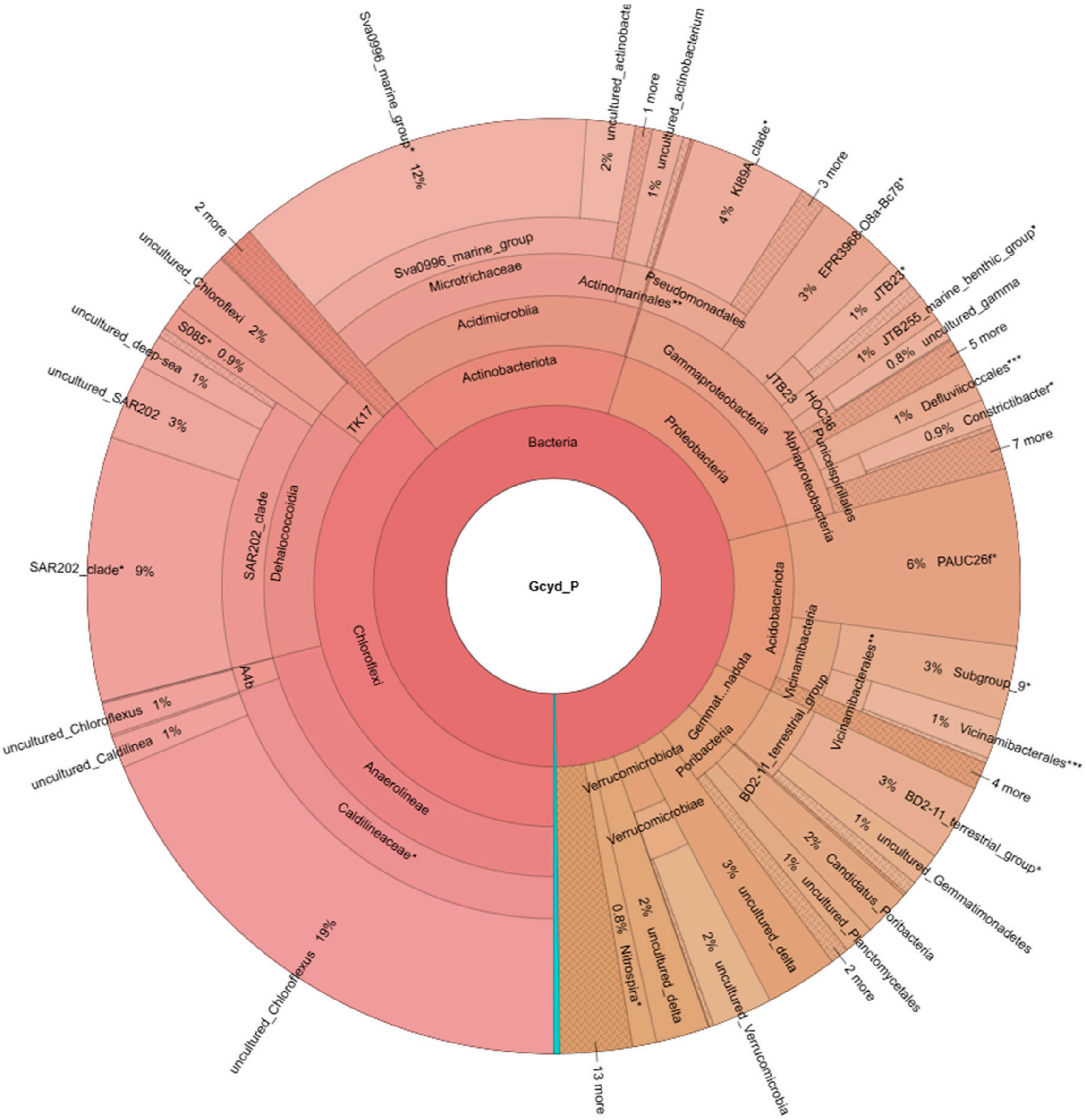

Gcyd_P
Bacteria
Chloroflexi
Actinobacteriota
Proteobacteria
Acidobacteriota
Gemmat...nadota
Verrucomicrobiota
Poribacteria
Acidimicrobiia
Microtrichaceae
Sva0996_marine_group
Actinomarinales**
Gammaproteobacteria
Alphaproteobacteria
Pseudomonadales
JTB23
HOC36
Puniceispirillales
Vicinamibacteria
Vicinamibacterales**
BD2-11_terrestrial_group
Verrucomicrobiae
Dehalococcoidia
SAR202_clade
TK17
A4b
Anaerolineae
Caldilineaceae*
Sva0996_marine_group* 12%
2% uncultured_actinobacte
1 more
1% uncultured_actinobacterium
4% KI89A_clade*
3 more
3% EPR3968-O8a-Bc78*
1% JTB23*
1% JTB255_marine_benthic_group*
0.8% uncultured_gamma
5 more
1% Defluviicoccales***
0.9% Constrictibacter*
7 more
6% PAUC26f*
3% Subgroup_9*
1% Vicinamibacterales***
4 more
3% BD2-11_terrestrial_group*
1% uncultured_Gemmatimonadetes
2% Candidatus_Poribacteria
1% uncultured_Planctomycetales
2 more
3% uncultured_delta
2% uncultured_Verrucomicrobia
2% uncultured_delta
0.8% Nitrospira*
13 more
uncultured_Chloroflexus 19%
uncultured_Caldilinea 1%
uncultured_Chloroflexus 1%
SAR202_clade* 9%
uncultured_SAR202 3%
uncultured_deep-sea 1%
S085* 0.9%
uncultured_Chloroflexi 2%
2 more

D)

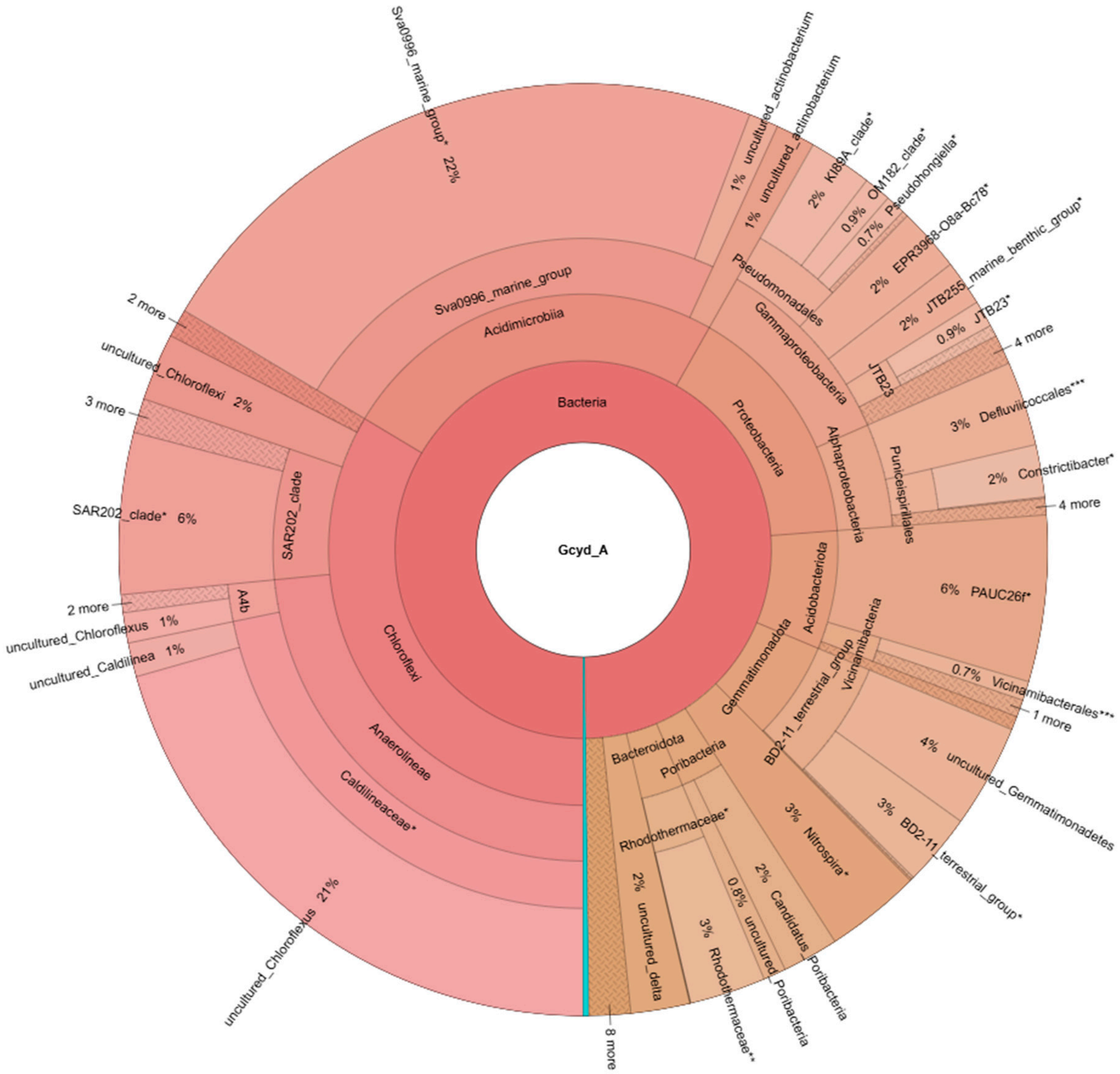
Gcyd_A
Bacteria
Chloroflexi
Anaerolineae
Caldilineaceae*
uncultured_Chloroflexus 21%
Acidimicrobiia
Sva0996_marine_group
Sva0996_marine_group* 22%
1% uncultured_actinobacterium
1% uncultured_actinobacterium
SAR202_clade
SAR202_clade* 6%
A4b
uncultured_Chloroflexi 2%
uncultured_Chloroflexus 1%
uncultured_Caldilinea 1%
2 more
3 more
2 more
Proteobacteria
Gammaproteobacteria
Pseudomonadales
Alphaproteobacteria
Puniceispirillales
2% KI89A_clade*
0.9% OM182_clade*
0.7% Pseudohongiella*
2% EPR3968-O8a-Bc78*
2% JTB255_marine_benthic_group*
0.9% JTB23*
JTB23
4 more
3% Defluviicoccales***
2% Constrictibacter*
4 more
Acidobacteriota
Vicinamibacteria
6% PAUC26f*
0.7% Vicinamibacterales***
1 more
Gemmatimonadota
BD2-11_terrestrial_group
4% uncultured_Gemmatimonadetes
3% BD2-11_terrestrial_group*
3% Nitrospira*
Bacteroidota
Poribacteria
Rhodothermaceae*
2% uncultured_delta
3% Rhodothermaceae**
0.8% uncultured_Poribacteria
2% Candidatus_Poribacteria
8 more

Figure S2. Venn diagram, graphical depiction of the genus taxa classified for each seample using R v4.5.1 (2025-06-13) and venn package v1.12.

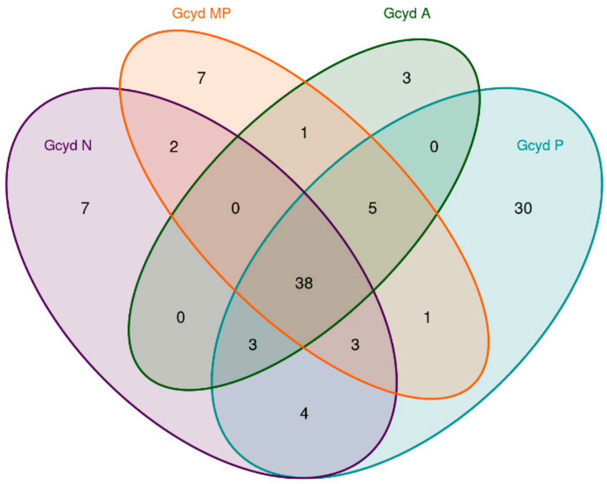

**Sample**				
**A (Gcyd N)**	**B (Gcyd P)**	**C (Gcyd MP)**	**D (Gcyd A)**	
**Row**				
D	C	C:D	B	B:D
B:C	B:C:D	A	A:D	A:C
A:C:D	A:B	A:B:D	A:B:C	A:B:C:D
**intersections**				
**intersections D**				
NS5_marine_group	Pseudomonadales**	Ascidiaceihabitans		
**intersections C**				
Silicimonas	Micavibrionaceae* OM75_clade	Ruegeria	SBR1031	KCM-B-112
Cyanobiaceae*				
**intersections C:D**				
Halieaceae*				
**intersections B**				
OM190	Alphaproteobacteria***	Sandaracinus	Roseibacillus	
Aquibacter	Albidovulum	Filomicrobium	Gaiellales**	
Chitinophagales**	Planctomicrobium	D90	Microtrichaceae*	
Chlamydiales**	UASB-TL25 Bythopirellula	Anderseniella		
Nitrosomonadaceae*	Urania-1B-19_marine_sediment_group	Subgroup_5	Blastopirellula	
Flavobacteriaceae*	Marinicaulis	Caenarcaniphilales	Candidatus_Spechtbacteria	
Rhizobiaceae*	Cyclobacteriaceae*	Ardenticatenaceae*	Planctomycetales**	
P3OB-42	Pirellulaceae*			
**intersections B:C**				
SAR324_clade(Marine_group_B)				
**intersections B:C:D**				
TK30	AT-s3-44	Rhodobacteraceae*	AqS1	P9X2b3D02
**intersections A**				
Lachnospiraceae*	SAR116_clade	Acidimicrobiaceae*	ML602J-51	Pseudovibrio
Peptostreptococcales-Tissierellales**	OM60(NOR5)_clade			
**intersections A:C**				
AEGEAN-169_marine_group Cyanobium_PCC-6307				
**intersections A:B**				
DEV007	Bdellovibrio NB1-j	Truepera		
**intersections A:B:D**				
AncK6	Subgroup_11 Subgroup_21			
**intersections A:B:C**				
S085	Candidatus_Kaiserbacteria	Endozoicomonas		
**intersections A:B:C:D**				
Sva0996_marine_group	A4b	UBA10353_marine_group	SAR202_clade	
Poribacteria	KI89A_clade	PAUC43f_marine_benthic_gr	PAUC26f	
BD2-11_terrestrial_group	Rhodothermaceae*	Subgroup_9	Constrictibacter	
Entotheonellaceae	Magnetospiraceae*	Caldilineaceae*	PAUC34f	
Nitrospira	Dadabacteriales	TK17	pItb-vmat-80	
EPR3968-O8a-Bc78	Actinomarinales**	OM182_clade	Pseudohongiella	
HOC36	Candidatus_Nitrosopumilus	Synechococcus_CC9902	JTB255_marine_benthic_group Kiloniellaceae*	
Spirochaeta	Cerasicoccus	Subgroup_10	Vicinamibacterales**	
bacteriap25	Defluviicoccales**	EF100-94H03		
JG30-KF-CM66				

Figure S3. Phylogenetic tree (reporting the distance between genera) illustrating the presence of bacteria and archaea in four samples of *G. cydonium* collected in IMTA system, Mar Piccolo (MPIC), Secca delle Fumose (Naples, NAP) and Polignano a Mare (POL).

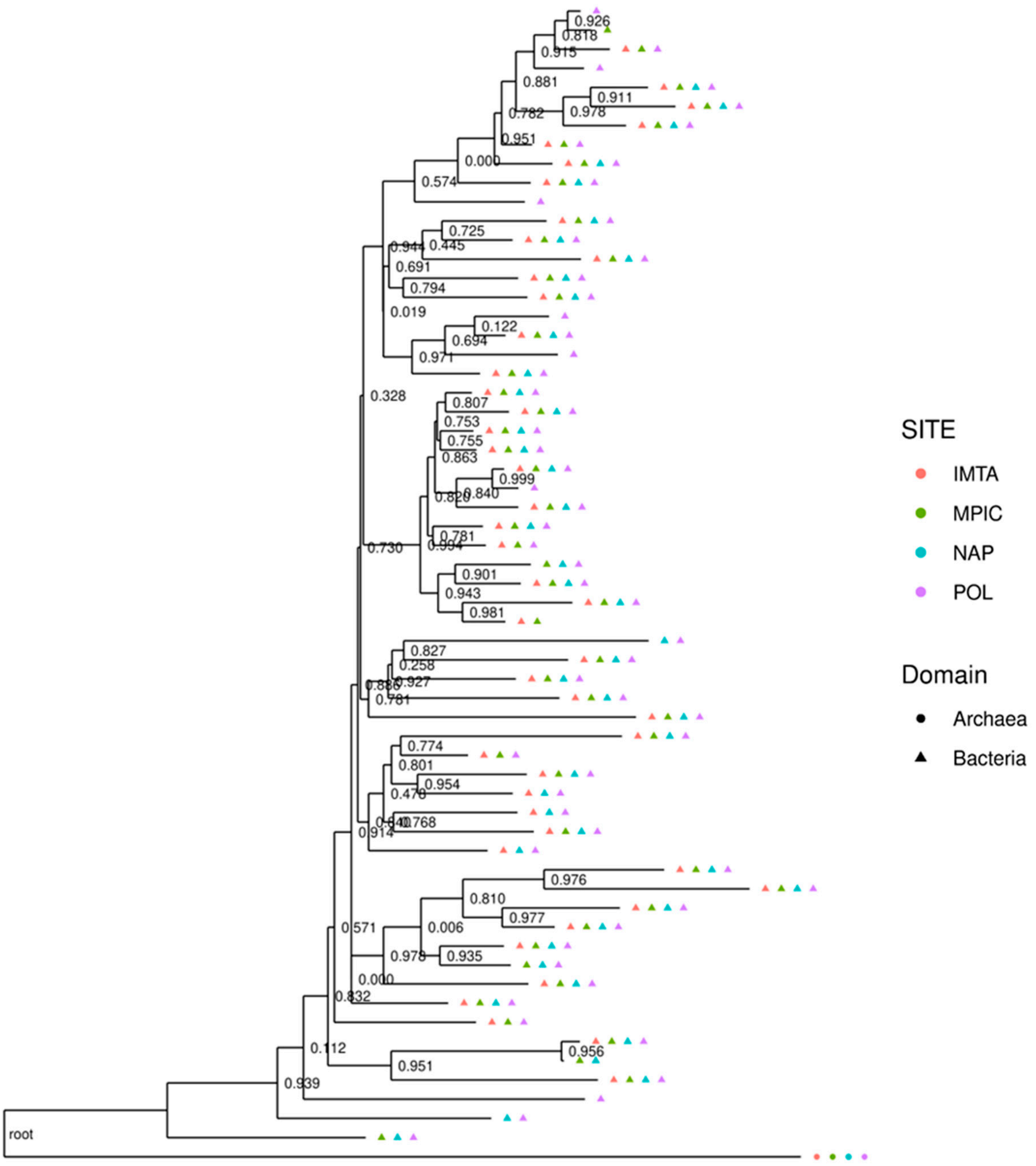

Supplement: Supplementary file 1 [file marinedrugs-24-00002-s001.zip › marinedrugs-3995323-supplementary.pdf]
